# 3D epigenome of glial cell types in developing human cortex

**DOI:** 10.1038/s41586-026-10987-6

**Published:** 2026-09-02

**Authors:** Ian R. Jones, Li Wang, Michael Kosicki, Stephanie L. Battle, Vivek JJ Narayan, Qiuli Bi, Kaila Gemenes, Yuxi Liu, Lingbo Zhou, Mengyi Song, Matthew White, Wendy Olson, Gabriel Beuchat, Diane Dickel, Yun Li, Len A. Pennacchio, R. David Hawkins, Arnold Kriegstein, Yin Shen

**Affiliations:** 1 Institute for Human Genetics, University of California, San Francisco, San Francisco, CA USA; 2 Department of Neurology, University of California, San Francisco, San Francisco, CA USA; 3 The Eli and Edythe Broad Center of Regeneration Medicine and Stem Cell Research, UCSF, San Francisco, CA USA; 4 Environmental Genomics and Systems Biology Division, Lawrence Berkeley National Laboratory, Berkeley, CA USA; 5 Division of Medical Genetics, Department of Medicine, Department of Genome Sciences, Institute for Stem Cell and Regenerative Medicine, University of Washington School of Medicine, Seattle, WA USA; 6 Department of Natural Sciences, College of Arts and Sciences, Bowie State University, Bowie, MD USA; 7 Department of Biostatistics, University of North Carolina at Chapel Hill, Chapel Hill, NC USA; 8 Department of Genetics, University of North Carolina at Chapel Hill, Chapel Hill, NC USA; 9 US Department of Energy Joint Genome Institute, Berkeley, CA USA; 10 Comparative Biochemistry Program, University of California Berkeley, Berkeley, CA USA; 11 Weill Institute for Neurosciences, University of California, San Francisco, San Francisco, CA USA

**Keywords:** Transcriptional regulatory elements, Glial biology

## Abstract

The human cortex is complex and heterogeneous, undergoing extensive expansion during development^1,2^. Our prior study of neurogenesis, including radial glia (RG), intermediate progenitor cells, excitatory neurons and interneurons demonstrated that chromatin looping underlies transcriptional regulation for lineage-specific genes, shedding light on how non-coding genetic variants contribute to neuropsychiatric disorders by means of cell-type-specific gene regulation^3^. RG have a crucial role in generating cellular diversity through both neurogenesis and gliogenesis and can be further classified into ventricular RG (vRG) and outer RG (oRG)^4,5^. Given their significance in cortical development, we conducted a comprehensive three-dimensional (3D) epigenomic analysis of four main glial populations, including vRG, oRG, oligodendrocyte precursor cells and microglia, from the mid-gestational human neocortex. By integrating gene expression, chromatin accessibility, DNA methylation and 3D chromatin interactions, we identified cell-type-specific candidate *cis*-regulatory elements (cCREs) and validated their regulatory function using transgenic mouse embryos. Using machine learning, we prioritized 112 schizophrenia risk variants within glia cCREs and further confirmed the predicted vRG enhancer disruption by the rs4449074 risk allele in vivo. Finally, oRG cCREs are enriched for human accelerated regions compared with other cCREs and a subset of human accelerated regions show activity differences from their chimpanzee orthologues that interact with genes involved in neuronal development. Our findings advance the understanding of human-specific gene regulation during corticogenesis.

## Abstract (web summary)

Integrative 3D epigenomic profiling of four glial cell types from the mid-gestation human cortex reveals cell-type-specific regulatory elements and chromatin interactions that illuminate the roles of non-coding variants in neuropsychiatric disease and human-specific cortical evolution.

## Main

The expansion of the human cortex is an extremely complex process that separates us from other mammals^6,7^. Radial glia (RG), the stem cells of the developing brain, are the driving force behind cortical expansion as they form a scaffold extending from the ventricular surface to the outermost pia that supports neuronal migration^8^. However, during mid-gestation, this scaffold becomes discontinuous as outer RG (oRG) delaminate from the ventricular surface, where ventricular RG (vRG) remain, and migrate towards the outer subventricular zone. In addition to different locations, vRG and oRG differ in the cell fate of their progeny^5^ and cell signalling pathways^7,9^. However, additional epigenomic profiling can provide further insight into mechanisms contributing to RG lineages and human-specific development, as much of our current knowledge is driven by transcriptional analysis. Although single-cell profiling of the developing brain highlights dynamic alterations of cell-type-specific epigenomes and transcriptomes^10^, the lack of integration of the three-dimensional (3D) epigenome with single-cell approaches poses substantial challenges to identifying mechanisms of epigenetic regulation, for example by chromatin loops, at the resolution required to analyse gene regulatory programs^11,12^. In addition, distal interacting regions detected by single-cell high-throughput chromatin conformation capture (Hi-C) tend to be less enriched for functionally validated enhancers compared with other modalities^13^, suggesting that these loops are more likely to represent structural interactions rather than promoter–enhancer loops.

Genome-wide association studies (GWAS) have identified thousands of variants associated with psychiatric disorders residing in non-coding regions, including *cis*-regulatory elements (CREs)^14,15^. Identifying causal variants remains challenging due to the heterogeneity of CREs across cell types^9^ as well as the difficulty of linking variants to their target genes as regulatory effects can span long genomic distances and do not necessarily affect the nearest gene^16,17^. Previously, we characterized cell populations involved in neurogenesis, including RG, intermediate progenitor cells (IPCs), excitatory neurons (eNs) and interneurons (iNs), highlighting how cell-type-specific epigenomic annotation can drive gene expression and prioritize disease-associated variants^3^. However, RG subtypes and additional key glial populations were absent. In this study, we perform a comprehensive analysis using gene expression, chromatin accessibility, DNA methylation and high-resolution 3D chromatin interactions, to identify candidate CREs (cCREs) and their regulatory targets in four main glial populations. We highlight loci containing epigenomic signals specific to vRGs and oRGs, spotlight transcription factors (TFs) that may contribute to lineage specification and detect enrichment of human accelerated regions (HARs) at oRG-specific cCREs. Furthermore, we train machine learning models to prioritize disease-associated variants and HARs in silico and conduct further validation of our predictions. These results provide new insights into gene regulatory control underlying human cortical development, disease and evolution.

## Characterizing cell-type-specific cCREs

**Fig. 1:**
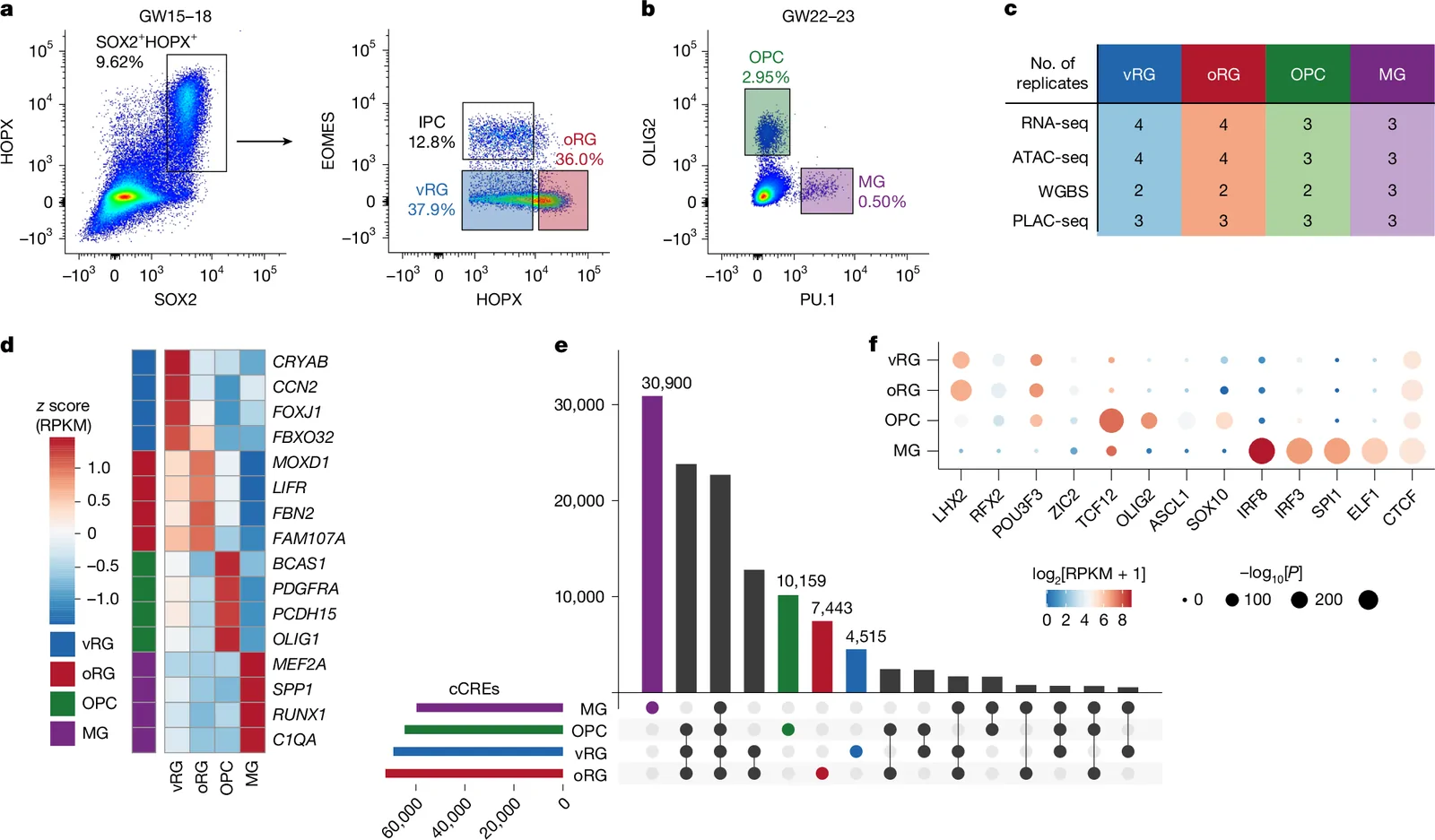
Collecting cell types and annotating cCREs within the developing cortex. **a**, Schematic of the sorting strategy for vRG and oRG. **b**, Schematic of the sorting strategy for OPC and MG. **c**, Table showing the number of replicates across each cell type for each assay. **d**, Heatmap of key marker genes for each cell type. RPKM, reads per kilobase per million mapped reads. **e**, Upset plot of cCREs. **f**, TF enrichment analysis for cell-type-specific cCREs. 4,515, 7,443, 10,159 and 30,900 peaks were used from vRG, oRG, OPC and MG, respectively. Colours represent RPKM expression of the corresponding TFs, and dot sizes represent enrichment *P* values from HOMER (one-sided binomial test).

**Extended Data Fig. 1:**
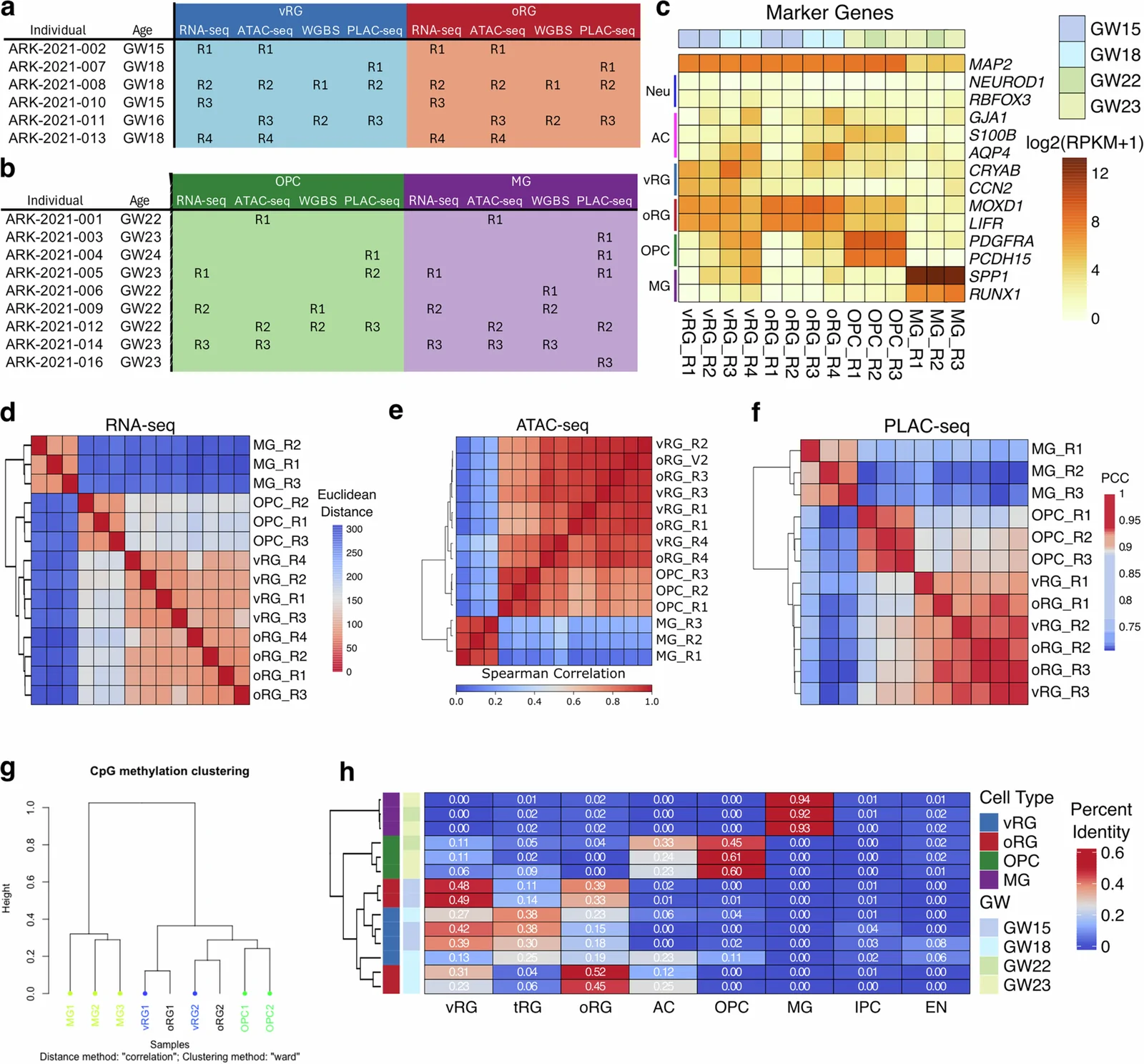
Replicate information and expression QC. **a, b**) Table displaying the number of replicates across each cell type for each assay. Replicate one (R1), replicate two (R2), replicate three (R3), replicate four (R4). **c**) Heatmap of log2(RPKM) values for key marker genes for each replicate. Astrocyte (AC), Neuronal (Neu). **d**) Hierarchical clustering of Euclidean distance for RNA-seq libraries performed post batch correction with DESeq2. **e**) Heatmap of Spearman correlation of ATAC-seq libraries within unified peaks across all four cell types. **f**) Heatmap of Pearson correlation of PLAC-seq libraries obtained with HPRep. **g**) Clustering of CpG methylation. **h**) Heatmap of cell distribution for bulk RNA-seq libraries imputed from scRNA-seq with CIBERSORTx.

To isolate distinct glial cell types, we leveraged cell-type-specific markers and fluorescence-activated cell sorting (FACS) using second trimester human cortex. We obtained vRGs and oRGs from 6 donors spanning gestational weeks (GW) 15 to 18, and microglia (MG) and oligodendrocyte precursor cells (OPCs) from 9 donors spanning GW22 to GW24 when gliogenesis begins^18^. We intentionally chose donors at different gestational age groups due to the fact that these cell types reach peak abundance at different developmental stages. Specifically, EOMES^−^, HOPX^+^ and SOX2^+^ cells were further separated by high HOPX expression for oRGs and low HOPX expression for vRGs^4^ (Fig. 1a). MG and OPCs were isolated as PU.1^+^ and OLIG2^+^ populations, respectively (Fig. 1b). In total we obtained between two and four replicates per cell type for each assay (Fig. 1c and Extended Data Fig. 1a,b). We first confirmed the reliability of our sorting strategies through the expression of marker genes from RNA sequencing (RNA-seq) (Fig. 1d and Extended Data Fig. 1c,d). Further correlation with single-cell RNA-seq (scRNA-seq) from second trimester primary cortical and medial ganglionic eminence samples^9^ corroborated successful cell sorting strategies and highlighted temporal difference within vRG and oRG between GW16 and GW18 consistent with the discontinuous scaffold (Extended Data Fig. 1h).

**Extended Data Fig. 2:**
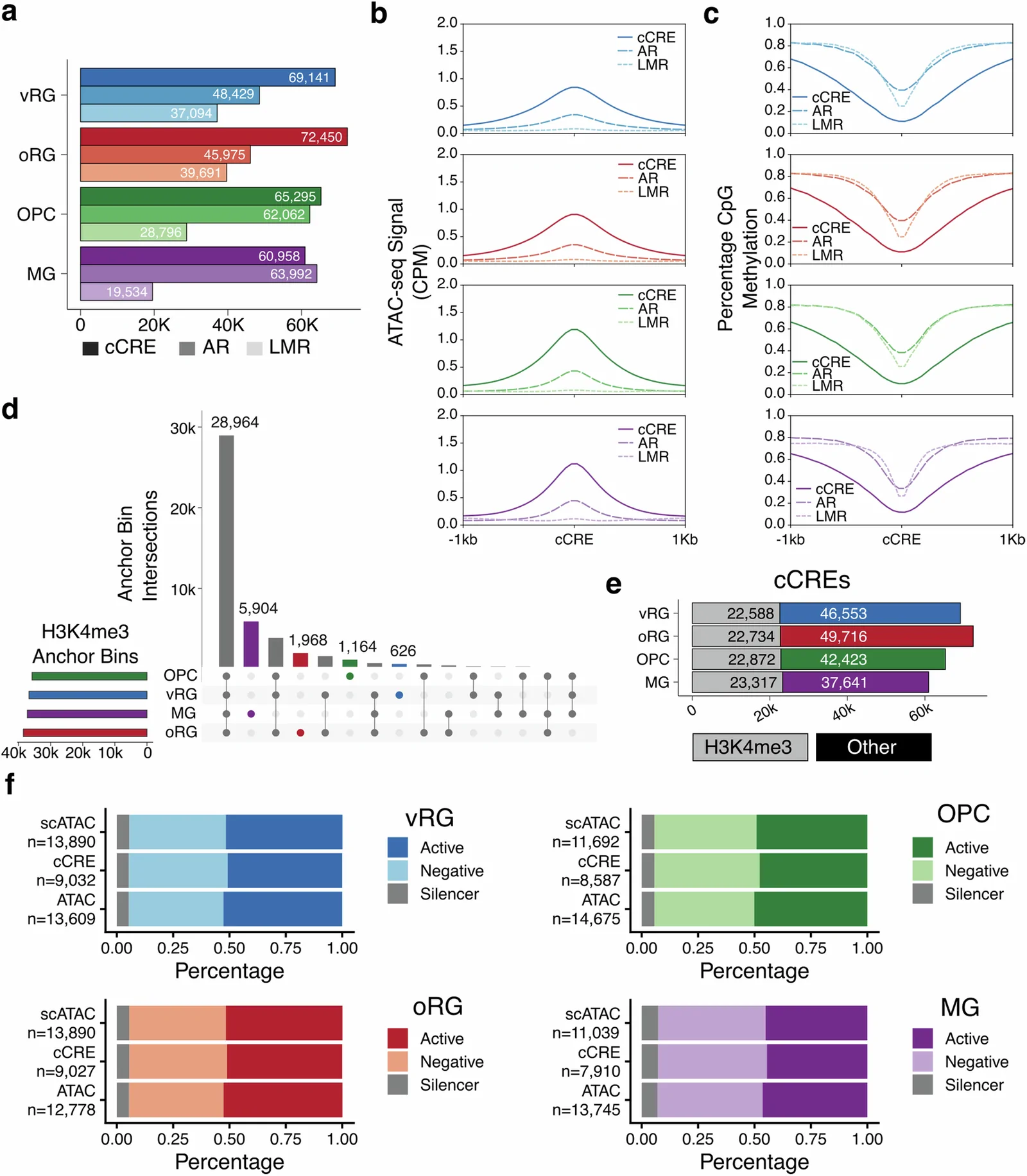
Defining cCREs with ATAC-seq and WGBS. **a**) Barplot of cCREs separated into three groups: lowly methylated accessible regions (cCRE), accessible regions (AR) or lowly methylated regions (LMR) as determined by ATAC-seq and WGBS. **b**) Average ATAC-seq signal centered on each cCRE. **c**) Average CpG methylation percentage centered on each cCRE. **d**) Upset plot of 2 kb anchor bins containing H3K4me3. **e**) Barplot of cCREs for each cell type. Grey indicates cCREs overlapping H3K4me3 bins and colored bar indicates other cCREs. **f**) Barplot of MPRA in mid-gestation human cortical cells that overlap cell type-specific peaks identified by scATAC^21^, cCREs, and ATAC-seq peaks. For scATAC-seq, microglia, radial glia, and astrocyte/oligodendrocyte accessible peaks were used.

Next, we performed assays for transposase-accessible chromatin with sequencing (ATAC-seq)^19^, whole-genome bisulfite sequencing (WGBS) and proximity ligation-assisted chromatin immunoprecipitation (ChIP) combined with sequencing (PLAC-seq)^20^ with H3K4me3 on sorted cell populations (Extended Data Fig. 1e–g and Supplementary Table 1). We defined cCREs as lowly methylated accessible regions. Those regions that are exclusively accessible (AR) or low methylated (LMR) are defined as cCREs^AR^ and cCREs^LMR^, respectively (Extended Data Fig. 2a). For downstream analyses, we focused on cCREs as they are more accessible and less methylated than either cCREs^LMR^ or cCREs^AR^ (Extended Data Fig. 2b,c). In total 69,141, 72,450, 65,295 and 60,958 cCREs were identified in vRG, oRG, OPC and MG, respectively, with more than 60% of cCREs residing in non-promoter regions, defined as not overlapping with H3K4me3 signals (Fig. 1e and Extended Data Fig. 2d,e). We performed TF binding motif enrichment analysis in cell-type-specific cCREs and identified known lineage-specific TF motifs in corresponding cell types, providing extra support for the success of our cell sorting strategy. For example, binding motifs for LHX2, SOX10 and interferon regulatory factors were enriched in RG, OPC and MG, respectively (Fig. 1f and Supplementary Table 2). Furthermore, a total of 9,032, 9,027, 8,587 and 7,910 cCREs from vRG, oRG, OPC and MG, respectively, were previously tested using a massively parallel reporter assay in mid-gestation human cortical cells and cerebral organoids^21^, representing 12.4–13.1% of cCREs in our study. Across all four cell types in our study, the fraction of cCREs showing enhancer activity in the massively parallel reporter assay, 44.5–51.1%, was comparable to that reported in the original study, which selected elements either overlapping H3K27ac signal or chromatin interactions (Extended Data Fig. 2f).

**Fig. 2:**
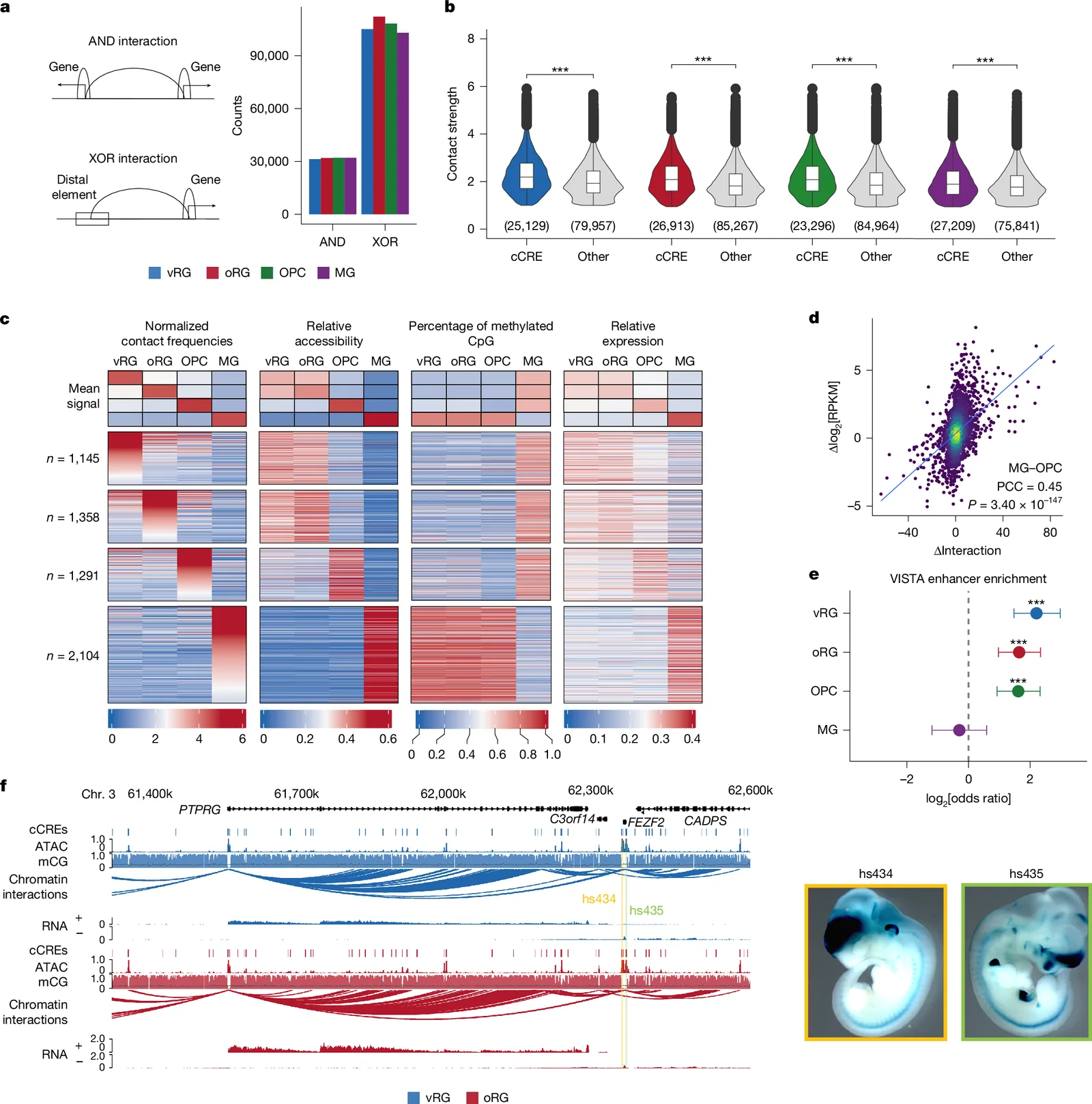
cCREs are associated with transcriptional regulation and enhancer activity. **a**, Left, diagram of AND interactions between two H3K4me3 bins and XOR interactions between H3K4me3 bins and distal bins. Right, bar plot of the number of interactions. **b**, Violin plots of the contact strength log_2_[observed/expected counts] in XOR interactions with cCREs and without cCREs in the distal bin (two-sided *t*-test, *P < 0.05, **P < 0.01, ***P < 0.001, 9.44 × 10^−150^ for MG; *P* < 1 × 10^−300^ for vRG, oRG and OPC). **c**, Heatmap showing normalized contact frequencies, chromatin accessibility, CpG methylation percentage and target gene expression of cell-type-specific XOR interactions with cCREs in the distal bin. **d**, Scatter plot showing the correlation between the difference in expression and number of interactions containing cCREs between OPC and MG (two-sided PCC = 0.45, and *P* = 3.4 × 10^−147^). **e**, Forest plot showing the association of cCREs with positive and negative neuronal VISTA elements for each cell type: vRG (*n* = 530 regions, *P* = 4.62 × 10^−7^), oRG (*n *= 519 regions, *P* = 4.66 × 10^−7^), OPC (*n *= 476 regions, *P* = 9.39 × 10^−7^), MG (*n* = 201 regions, *P* = 0.5577). Each point represents the log_2_ odds ratio, with the error bars indicating 95% confidence intervals (two-sided Fisher’s exact test, *P < 0.05, **P < 0.01, ***P < 0.001). **f**, Browser session of VISTA element hs434 (light yellow highlight) and hs435 (light green highlight) shown to interact with *PTPRG* in both vRG (blue) and oRG (red).

**Extended Data Fig. 3:**
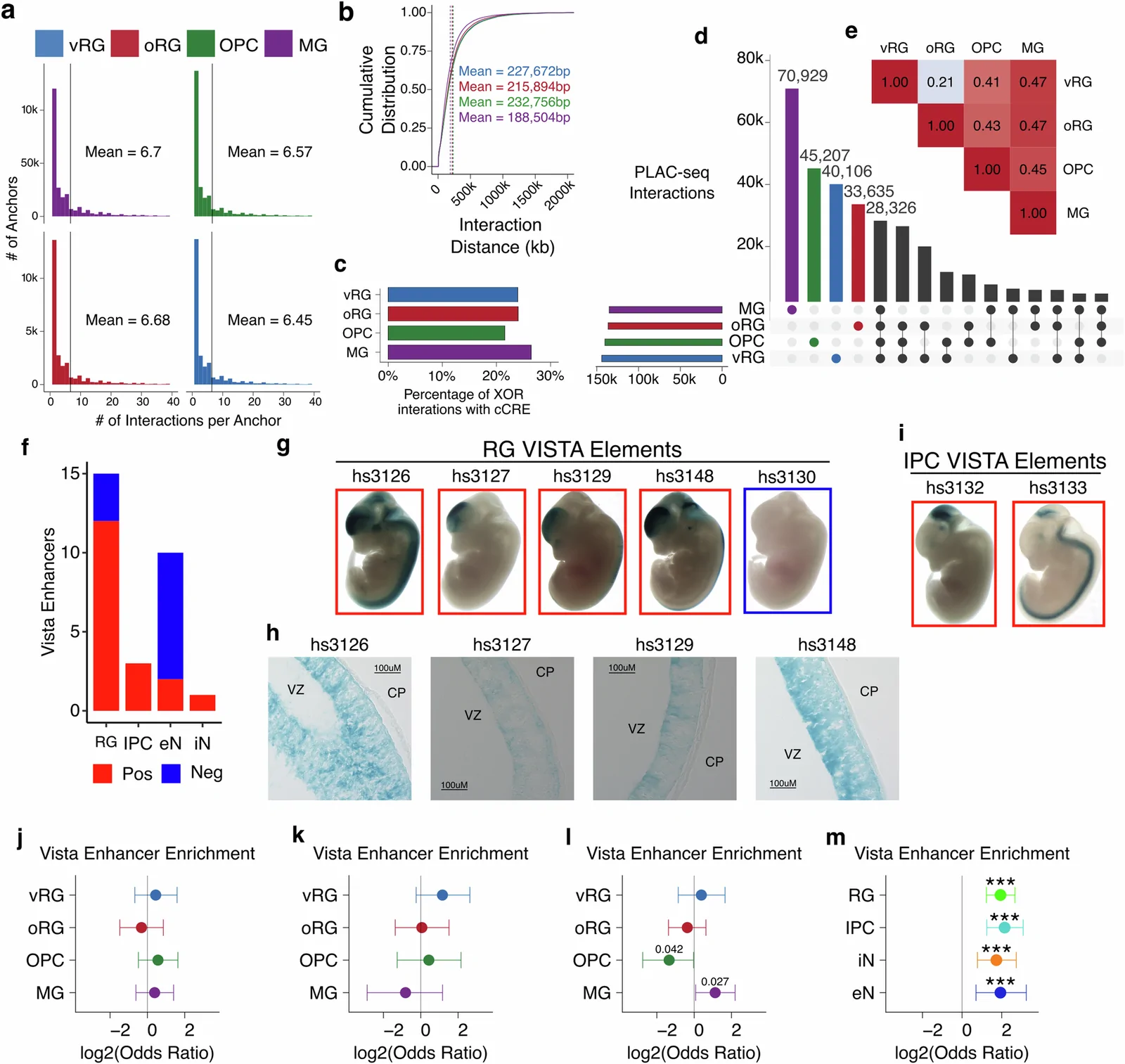
cCREs and VISTA enhancers involved in the 3D epigenome of the developing brain. **a**) Barplot of the number interactions per anchor. Average plotted as a vertical line. **b**) Cumulative distribution plot of distances of significant interactions. The mean distance is plotted as a dotted line. **c**) Barplot of the classification of all significant interactions. **d**) Upset plot of both AND and XOR PLAC-seq interactions. **e**) Heatmap of Pearson correlation between change in expression and interactions with cCREs not overlapping H3K4me3 anchors. **f**) Bar plot of VISTA results. **g**) VISTA results for select RG peaks at e12.5. **h**) Brain sections, two embryos per element visualized, of RG VISTA elements. (VZ) ventricular zone. (CP) cortical plate. **i**) VISTA results for select IPC peaks at e12.5. (**j-l**) Forest plot showing the association of (**j**) cCREs^AR^, (**k**) cCREs^LMR^, and (**l**) distal interacting bins lacking accessibility or low methylation with positive neural VISTA elements or negative neuronal VISTA elements. Each point represents the log2 odds ratio, with the error bars indicating 95% confidence intervals. 44–156 total genomic regions tested. **m**) Forest plot showing the association of ATAC-seq peaks with positive neural VISTA elements or negative neuronal VISTA elements. Each point represents the log2 odds ratio, with the error bars indicating 95% confidence intervals. 449, 321, 198, and 235 total genomic regions tested in RG (*P* = 1.28 × 10^−08^), IPC (*P* = 3.16 × 10^−07^), eN (*P* = 1.19 × 10^−03^), and iN (*P* = 1.60 × 10^−4^) respectively.

Our high-quality H3K4me3-mediated PLAC-seq datasets led to the identification of 136,389, 144,089, 140,297 and 135,123 significant chromatin interactions from H3K4me3 marked promoters at a resolution of 2 kb in vRG, oRG, OPC and MG, respectively (Fig. 2a), fourfold more interactions than previous PLAC-seq libraries that studied neurogenesis with 5 kb resolution^3^. As a result, our datasets are much better at defining gene regulatory chromatin loops at higher resolution compared with single-cell Hi-C studies^10,22^. Most significant interactions (roughly 80%) are between a promoter and a distal region (XOR interactions) and the rest are between two H3K4me3 marked promoters (AND interactions). On average, a 2-kb bin with H3K4me3 mark participates in 6.45–6.7 interactions with a mean interaction distance between 188,000 bp and 233,000 bp (Extended Data Fig. 3a,b). Of the XOR interactions, 21.5–26.4% contained cCREs in the 2-kb distal bin averaging a higher contact frequency (observed/expected contacts) with H3K4me3 marked promoters than those without cCREs (Fig. 2b and Extended Data Fig. 3c). This suggests that cCREs have a role in the 3D architecture of the genome.

Consistent with previous findings that chromatin interactions are associated with cell-type-specific gene-expression changes during neurogenesis^3^, XOR interactions unique to one cell type involving cCREs within the distal bin from the matching cell type are positively correlated with chromatin accessibility and gene expression, and showed a negative correlation with CpG (5′–C–phosphate–G–3′) methylation (Fig. 2c and Extended Data Fig. 3d). Notably CpG methylation shows a lower correlation, specifically between vRGs and oRGs, consistent with the finding that RG subtypes are only identified by chromatin conformation signatures and not by DNA methylation signatures based on single-cell multi-omic data^10^. Genome-wide, the differences between promoter-interacting cCREs and gene expression across different cell types result in a significantly positive correlation (Fig. 2d, Extended Data Fig. 3e and Supplementary Table 3), further confirming the regulatory relationship between cCREs and their interacting genes.

## cCREs show enhancer activity in vivo

We next performed transgenic enhancer–reporter assays in mice to validate cCREs activity. We initially selected 15 elements annotated in RGs, along with 3 IPC, 10 eN and 1 iN cCREs based on our previous work^3^ (Methods). Of RG and IPC elements, 77.8% (14 out of 18) showed enhancer activity in the embryonic brain, confirming their role in gene regulation in vivo (Extended Data Fig. 3f–i). Moreover, of the 11 positive RG enhancers, 10 were identified as cCREs in both vRGs and oRGs revealing that cCREs could be ideal markers for function (Supplementary Table 4). Meanwhile, only 27.3% (3 out of 11) of eN and iN cCREs showed positive neural signals (Extended Data Fig. 3f), as expected, given that the embryonic stage (e12.5) used in this assay is at the peak of abundance of progenitor cells with a relative paucity of neurons^23^. Notably, four strongly positive RG elements showed positive signal in the ventricular zone as expected (Extended Data Fig. 3h). More signal can be found near the cortical plate in hs3126 and hs3127, suggesting that elements can be active in other cell types as well.

Next, we assessed enrichment of 1,233 functional neural enhancers relative to 1,868 negative elements annotated in the VISTA Enhancer Browser^24,25^ among promoter-interacting cCREs identified in our study, compared with distance-matched controls (Methods). Neural enhancers are enriched in interacting cCREs in vRG, oRG and OPC, but not MG (Fig. 2e). Comparatively, they were not associated with cCREs^AR^ and cCREs^LMR^, further highlighting the enhancer activity of cCREs (Extended Data Fig. 3j,k). Distal interacting bins lacking accessibility or low methylation, which account for roughly 75% of XOR interactions, showed no or weak enrichment for VISTA enhancers, indicating minimal enhancer-like activity in these regions (Extended Data Fig. 3l). Furthermore, ATAC-seq peaks from RG, IPC, eN and iN^3^ also showed strong association with VISTA neural enhancers (Extended Data Fig. 3m), with RG and IPCs having the greatest association consistent with transgenic enhancer–reporter assay results (Extended Data Fig. 3f).

We leveraged our 3D epigenomic data to annotate potential target genes of VISTA neural enhancers. For example, significant chromatin interactions link the VISTA elements hs434 in vRG and oRG and hs435 in vRG to the protein tyrosine phosphatase receptor type G gene (*PTPRG*) that is primarily expressed within the nervous system^26^. Both hs434 and hs435 show forebrain activity and are positioned more than 800 kb from *PTPRG* (Fig. 2f). Gene ontology analysis of 589 genes interacting with cCREs overlapping VISTA validated neural enhancers are enriched for biological processes, including ‘nervous system development’, ‘forebrain development’ and ‘neural precursor cell proliferation’ (Supplementary Table 4).

## Regulatory landscapes of vRG and oRG

**Fig. 3:**
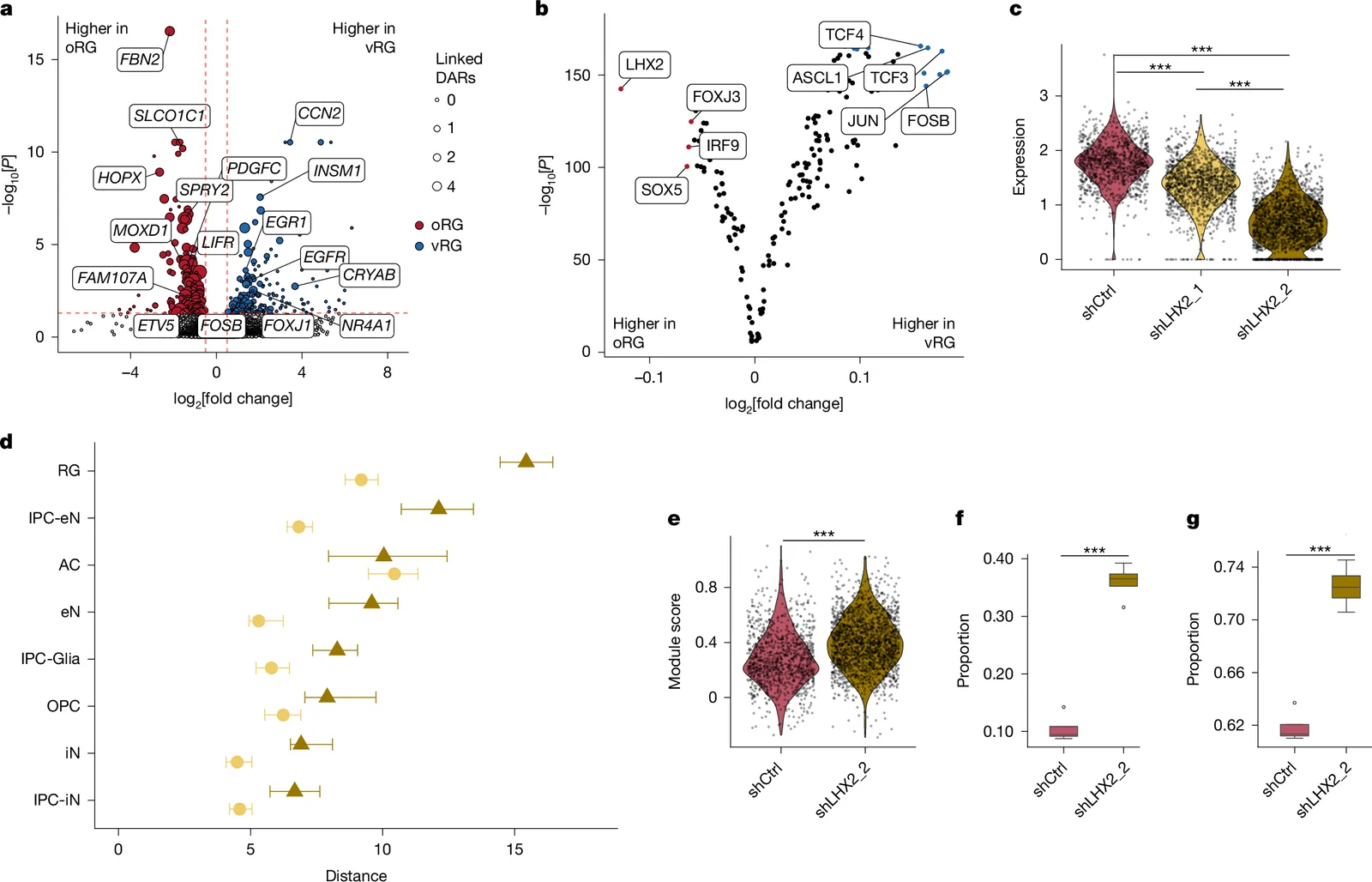
Identifying TFs driving epigenomic changes between vRG and oRG. **a**, Volcano plot of DEGs. The size of each dot is determined by the amount of DARs overlapping the TSS or interacting with each gene. *P* values were calculated using DESeq2 and adjusted (adj) for multiple testing using the Benjamini–Hochberg false discovery rate correction. **b**, Volcano plot of motif binding difference between vRG and oRG, determined by TOBIAS BINDetect. *P* values were calculated using a two-sided one-sample *t*-test (TOBIAS BINDetect default). **c**, Violin plot of *LHX2* expression within the oRG cluster (two-sided Wilcoxon rank sum test, *adj-*P* < 0.05, **adj-*P* < 0.01, ***adj-*P* < 0.001, shCtrl versus shLHX2_1 adj-*P* = 1.11 × 10^−105^, shCtrl versus shLHX2_2 adj-*P* < 1 × 10^−300^, shLHX2_1 versus shLHX2_2 adj-*P* = 3.67 × 10^−284^). **d**, Distance score obtained by scDist for each cell type. Data presented as distance score ±95% confidence interval (*n* = 4 independent experiments). **e**, Violin plot of the module score of the 16 DEGs targets of the LHX2 motif within the oRG cluster (Wilcoxon rank sum test, *P* = 1.55 × 10^−56^). **f**, Box plot showing the proportion of oRG cells among all captured cell types, analysed using scCODA (false discovery rate less than 0.001). Box boundaries represent the Q1 and Q3 quartiles, with the median indicated by the central line. Whiskers extend to the minimum and maximum values, and outliers are shown as individual points (*n* = 4 independent experiments). **g**, Box plot showing the proportion of cells in the G1 state across all clusters, analysed using a two-sided paired *t*-test (*P* = 7.6 × 10^−^^5^). Box boundaries represent Q1 and Q3 quartiles, with the median indicated by the central line. Whiskers extend to the minimum and maximum values, and outliers are shown as individual points (*n* = 4 independent experiments).

**Extended Data Fig. 4:**
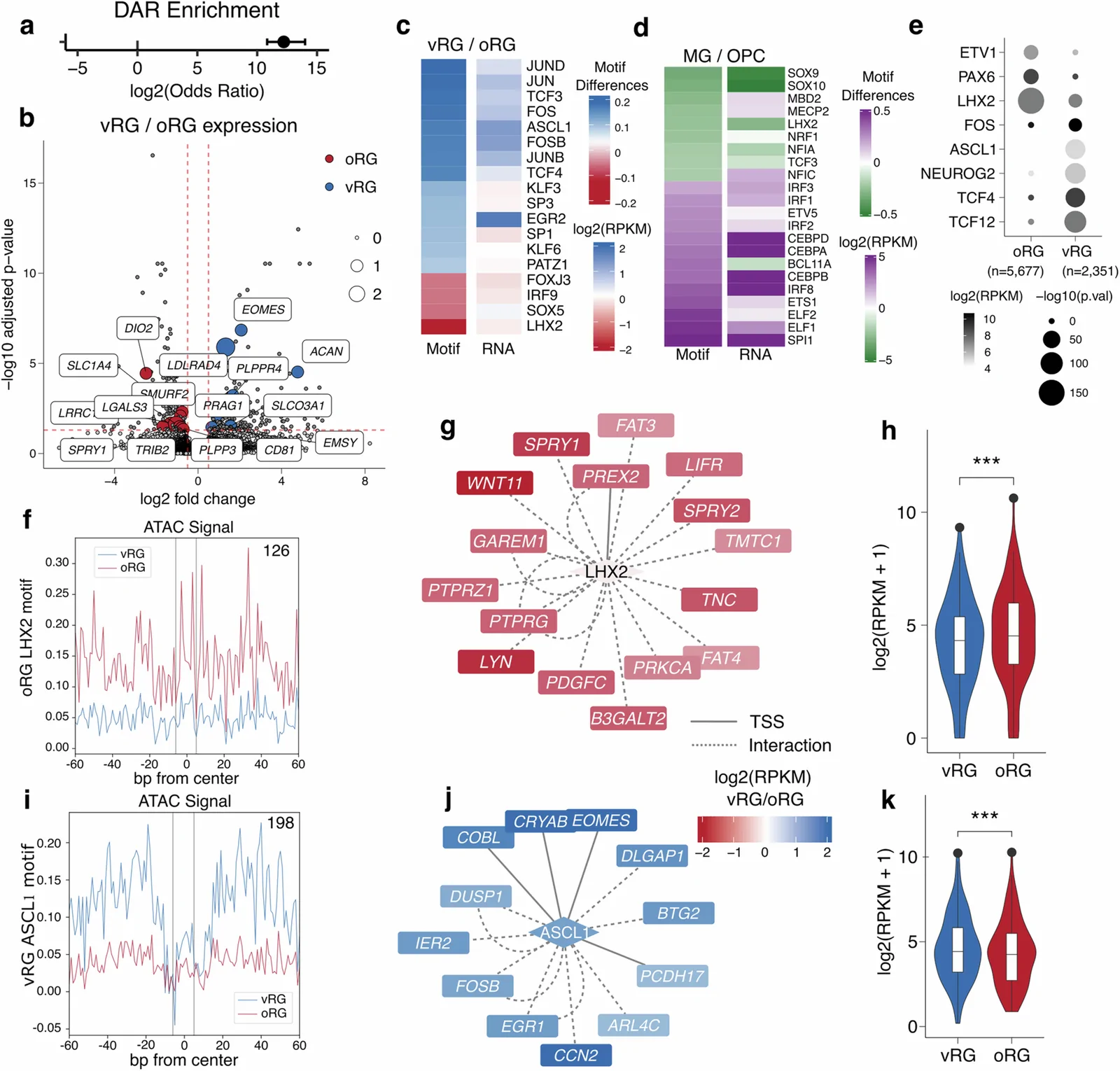
Identifying differential chromatin accessibility, methylation, and motif enrichment and its association with transcription. **a**) Forest plot showing the association of vRG or oRG DARs compared to DARs of scATAC-seq from primary human forebrain at midgestation. Each point represents the log2 odds ratio, with the error bars indicating 95% confidence intervals (n = 1,883 total genomic regions tested). **b**) Volcano plot of differentially expressed genes. The size of each dot is determined by the number of DMRs overlapping the TSS or interacting with each gene. *P* values were calculated using DESeq2 and adjusted for multiple testing using the Benjamini-Hochberg false discovery rate (FDR) correction. **c**) Heatmap of predicted transcription factor binding difference (Motif) and RPKM difference (RNA) between vRG and oRG. **d**) Heatmap of predicted transcription factor binding difference (Motif) and RPKM difference (RNA) between OPC and MG. **e**) TF motif enrichment analysis for vRG and oRG DARs. Sizes represent enrichment scores based on the p-values from HOMER (one-sided binomial test). Colors represent the gene expression of the corresponding TFs. **f**) Aggregated footprints of the LHX2 motif predicted to be more greatly bound in oRG compared with vRG (n = 126). **g**) Gene network of DEGs interacting with (dotted line) or have promoters overlapping (solid line) LHX2 motifs predicted to be bound specifically in oRG (n = 127). **h**) RPKM expression of all genes (n = 87) interacting with or overlapping LHX2 TFBS predicted to be bound specifically in oRG (two-sided paired t-test, *P* = 4.02 × 10^−7^). Box boundaries represent the Q1 and Q3 quartiles with the median indicated by the central line. Whiskers extend to the minimum and maximum values, and outliers are shown as individual points. **i**) Aggregated footprints of the ASCL1 motif predicted to be more strongly bound in vRG compared with oRG (n = 198).** j**) Gene network of DEGs interacting with (dotted line) or have promoters overlapping (solid line) ASCL1 TFBSs predicted to be bound specifically in vRG (n = 198). **k**) RPKM expression of all genes (n = 116) interacting with or overlapping ASCL1 TFBSs predicted to be bound specifically in vRG (two-sided paired t-test, *P* = 4.68 × 10^−6^). Box boundaries represent the Q1 and Q3 quartiles, with the median indicated by the central line. Whiskers extend to the minimum and maximum values, and outliers are shown as individual points.

Neural progenitors, vRG and oRG, are more closely related than either OPC or MG, requiring more precise techniques to dissect epigenetic changes associated with either lineage. oRG are a key class of neural stem cells in the outer subventricular zone that have undergone significant expansion in the primate lineage and are rare or absent in rodents^27,28^. As oRG are thought to be an important cell type driving cortical expansion, systematic epigenomic comparison between vRG and oRG could improve our understanding of the uniqueness of human cortical development. To achieve this, we called 7,941 differentially accessible regions (DARs) and 756 differentially methylated regions (DMRs) (Supplementary Table 5). Notably, tenfold more DARs were identified than DMRs, consistent with previous results that DNA methylation poorly separates RG subtypes (Fig. 2c). Moreover, DARs significantly overlap previous differential peaks identified with pseudo bulk scATAC-seq from primary human forebrain at mid-gestation^29^ (Fisher’s exact test, odds ratio = 5,108, *P *< 2.2 × 10^−16^) (Extended Data Fig. 4a). We next integrated DARs and DMRs with differentially expressed genes (DEGs), resulting in 26% and 63% of vRG and oRG DEGs having DARs overlapping at transcription start sites (TSSs) or interacting with distal DARs, compared with only 3.8% and 5.6% with DMRs (Fig. 3a and Extended Data Fig. 4b).

To examine which TFs could cause epigenomic changes, we first used TOBIAS^30^, a footprinting framework that can predict TF binding sites (TFBS) based on decreased signal at motifs as well as identify global TFBS differences between two cell types. ASCL1, TCF4 and TCF12 TFBS are associated with vRG open chromatin, whereas LHX2 TFBS are associated with oRG open chromatin (Fig. 3b). In addition to their role in proliferation, these TFs have been implicated in various neuropsychiatric disorders^31–33^. The aforementioned motif binding differences are also significantly correlated with corresponding TF expression differences (Pearson correlation coefficient (PCC) = 0.54, *P* = 0.009) (Extended Data Fig. 4c). A similar analysis comparing OPC and MG identified differences in predicted binding at motifs for known lineage TFs SOX10 and SPI1 in OPC and MG, respectively, as well as a positive correlation between TFBS and expression changes (PCC = 0.66, *P* = 0.00086) (Extended Data Fig. 4d). Second, motif enrichment within vRG and oRG DARs with HOMER^34^ recapitulated the association of ASCL1 and LHX2 TFs with vRGs and oRGs, respectively (Extended Data Fig. 4e and Supplementary Table 5).

To understand the influence of TFs on gene networks, we investigated the gene targets interacting with LHX2 and ASCL1 TFBS. The LHX2 motif has 126 predicted TFBS specific to oRGs (Extended Data Fig. 4f, Supplementary Table 6 and Methods). By incorporating oRG chromatin interactions, 87 target genes of TFBS more highly expressed in oRGs were elucidated, including 18.4% (*n *= 16) oRG DEGs such as *PDGFC*, *SPRY1* and *WNT11* (two-sided paired *t*-test, *P* = 3.811 × 10^−^^7^) (Extended Data Fig. 4g,h). The ASCL1 motif has 198 TFBS specific to vRGs targeting 116 genes that are on average more highly expressed, including 10.3% (*n* = 12) vRG DEGs (Extended Data Fig. 4i–k and Supplementary Table 6) (two-sided paired *t*-test, *P* = 4.679 × 10^−6^).

**Extended Data Fig. 5:**
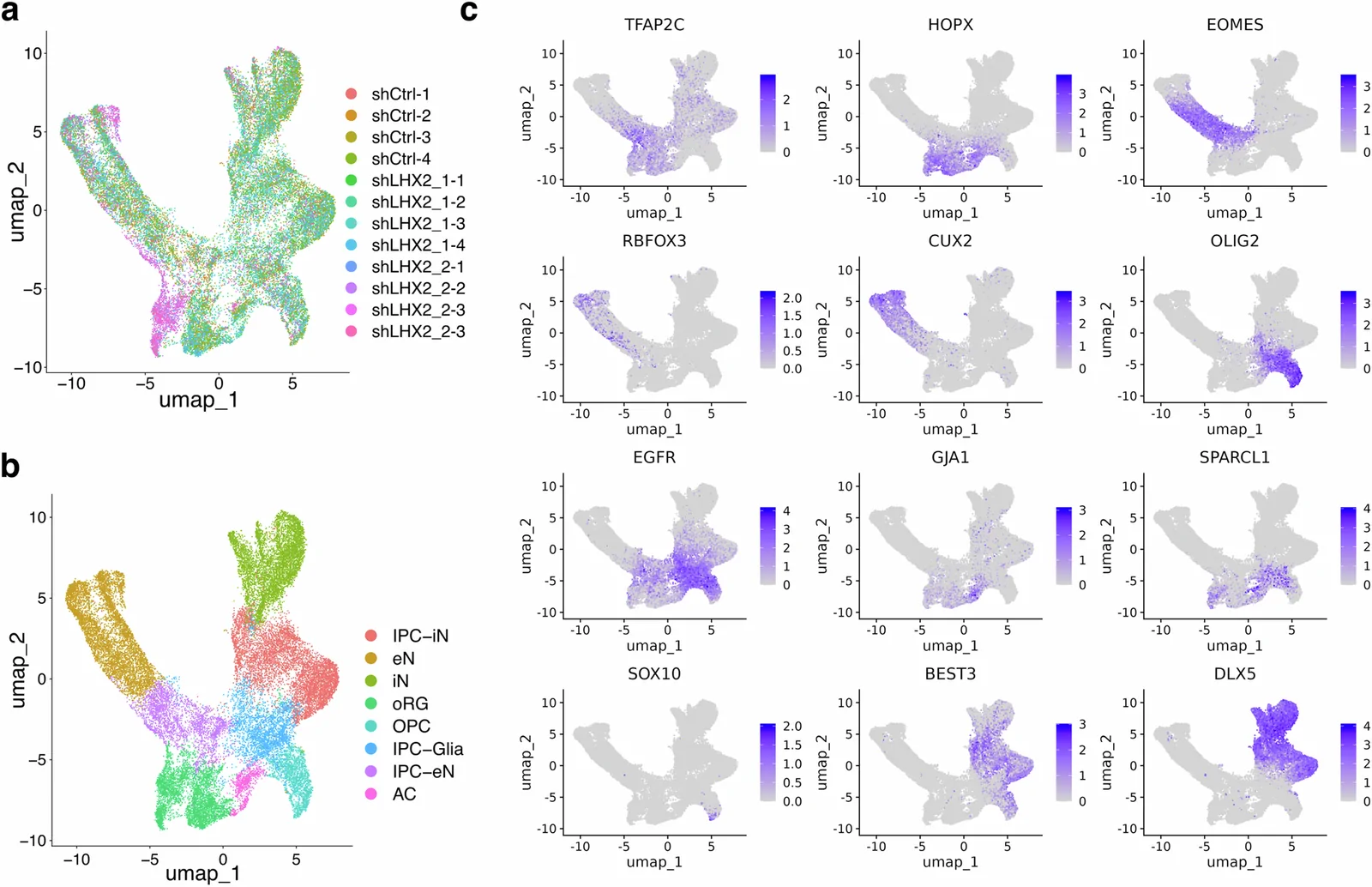
scRNA-seq Clustering. **a, b**) UMAP plots of oRGs after 7 days of in vitro differentiation based on scRNA-seq colored by (a) replicate, and (b) cell types. **c**) Scatterplot displaying the feature importance for each gene determined by scDist. Top 3 genes are highlighted.

**Extended Data Fig. 6:**
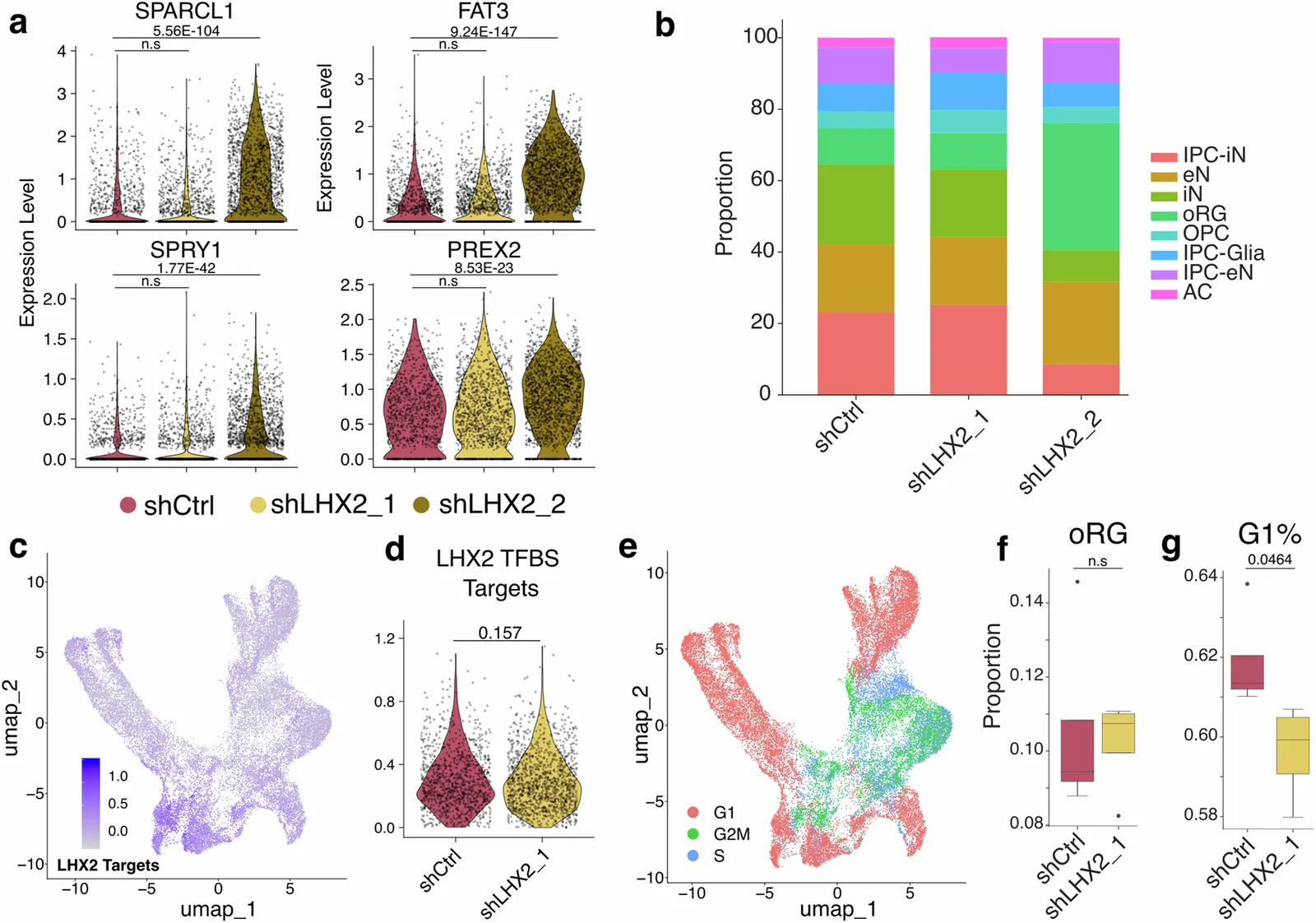
scRNA-seq analysis of LHX2 perturbation. **a**) Violin plots of the expression within the oRG cluster of genes perturbed by LHX2 knock down (two-sided Wilcoxon Rank Sum test). **b**) Barplots of the average proportion of cell types across four replicates. **c**) UMAP plots of oRGs after 7 days of in vitro differentiation based on scRNA-seq colors by module score of the 16 DEGs targets of the LHX2 motif within the oRG cluster **d**) Violin plot of the module score of the 16 DEGs targets of the LHX2 motif within the oRG cluster (two-sided Wilcoxon Rank Sum test). **e**) UMAP plots of oRGs after 7 days of in vitro differentiation based on scRNA-seq colors by cell cycle phase. **f**) Boxplot of the proportion of oRG (scCODA, n.s: not significant). Box boundaries represent Q1 and Q3 quartiles, with the median indicated by the central line. Whiskers extend to the minimum and maximum values, and outliers are shown as individual points (n = 4 independent experiments). **g**) Boxplot of the proportion of cells at the G1 state within all clusters (two-sided paired T-test). Box boundaries represent Q1 and Q3 quartiles, with the median indicated by the central line. Whiskers extend to the minimum and maximum values, and outliers are shown as individual points (n = 4 independent experiments).

To assess the role of LHX2 in oRG, we performed short hairpin RNA (shRNA)-mediated knockdown followed by scRNA-seq to examine its impact on transcription and neuronal differentiation. Uniform manifold approximation and projection (UMAP) clustering identified eight distinct cell types, with minimal batch effects (Extended Data Fig. 5a–c). Successful knockdown of LHX2 was confirmed within the oRG cluster, with shLHX2_2 producing a more pronounced reduction than shLHX2_1 (Fig. 3c). To identify cell types that showed transcriptomic dysregulation on LHX2 knockdown, we applied scDist^35^, a statistically rigorous method to fairly rank transcriptomic changes on the basis of the Euclidean distance. Both shRNAs produced concordant directional transcriptomic changes, but the effect was substantially greater with shLHX2_2, consistent with its stronger knockdown efficiency (Fig. 3d). Subsequent analyses therefore focused on shLHX2_2. The oRG and astrocyte clusters were the most perturbed, with the astrocyte marker *SPARCL1* being of high feature importance (Extended Data Fig. 6a), in agreement with previous work suggesting that LHX2 is required to repress astrogenesis^36^. In addition to the increase of *SPARCL1*, we observed a general upregulation of the 16 DEGs predicted as targets of LHX2 TFBS, including *FAT3*, *SPRY1*, *PRKCA* and *PREX2* (Fig. 3e and Extended Data Fig. 6a,c). These results support a model in which LHX2 regulates astrocyte-associated genes and downstream differentiation trajectories. Furthermore, the relative abundance of oRG cells increased significantly following shLHX2_2 knockdown at the expense of differentiated neurons (Fig. 3f and Extended Data Fig. 6b). Despite this relative expansion, shLHX2_2 showed a higher proportion of cells in G1 phase (Fig. 3g and Extended Data Fig. 6e), indicating reduced proliferative activity and the onset of a transition towards differentiation or quiescence. Taken together, loss of LHX2 reduces self-renewal and shifts oRG towards a more differentiated, astrocyte-like state. Consistent with a weaker perturbation, shLHX2_1 showed a similar transcriptomic trend but did not produce comparable compositional or cell-cycle changes, probably due to incomplete depletion of LHX2 (Extended Data Fig. 6d,f,g).

## Neuropsychiatric risk across cell types

**Fig. 4:**
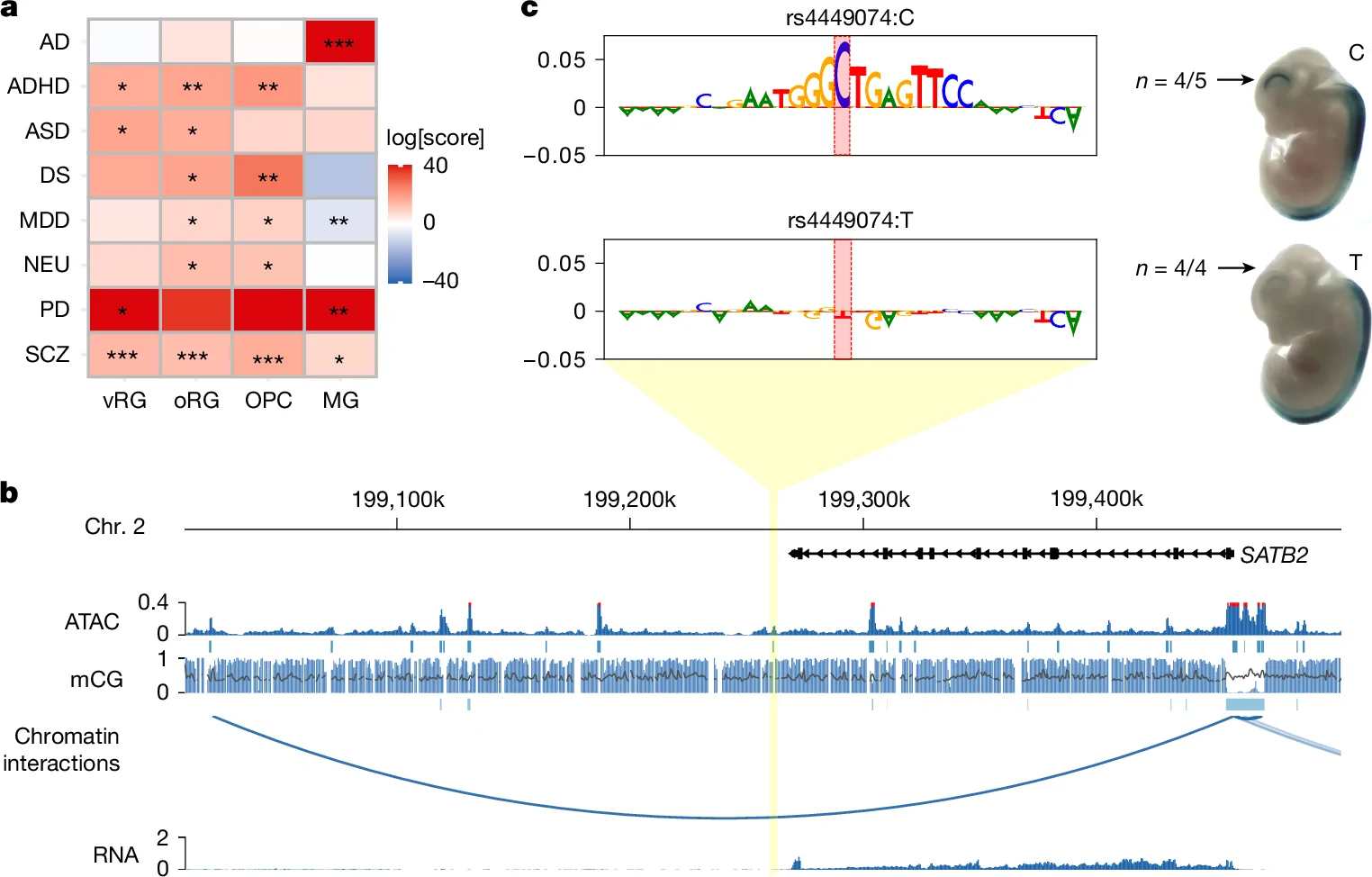
Prioritizing neuropsychiatric disorder variants within the developing brain. **a**, Heatmap of LDSC score for interacting cCRE. Red, the disorder is positively enriched. Blue, the disorder is negatively enriched (two-sided LDSC enrichment *P* values *P < 0.05, **P < 0.01, ***P < 0.001). AD, Alzheimer’s disease; ADHD, attention deficit hyperactivity disorder; DS, depressive symptoms; MDD, major depressive disorder; NEU, neuroticism; PD, Parkinson’s disease. **b**, WashU browser of the *SATB2* locus for vRG. Accessible chromatin containing rs4449074 is highlighted in yellow. **c**, Left, GkmExplain importance scores for each base pair surrounding rs4449074 reference or non-risk allele C (top) and the alternative or risk allele T (bottom) highlighted in red. Right, VISTA results at e12.5 for rs4449074 allele C (top) and for rs4449074 risk allele T (bottom).

**Extended Data Fig. 7:**
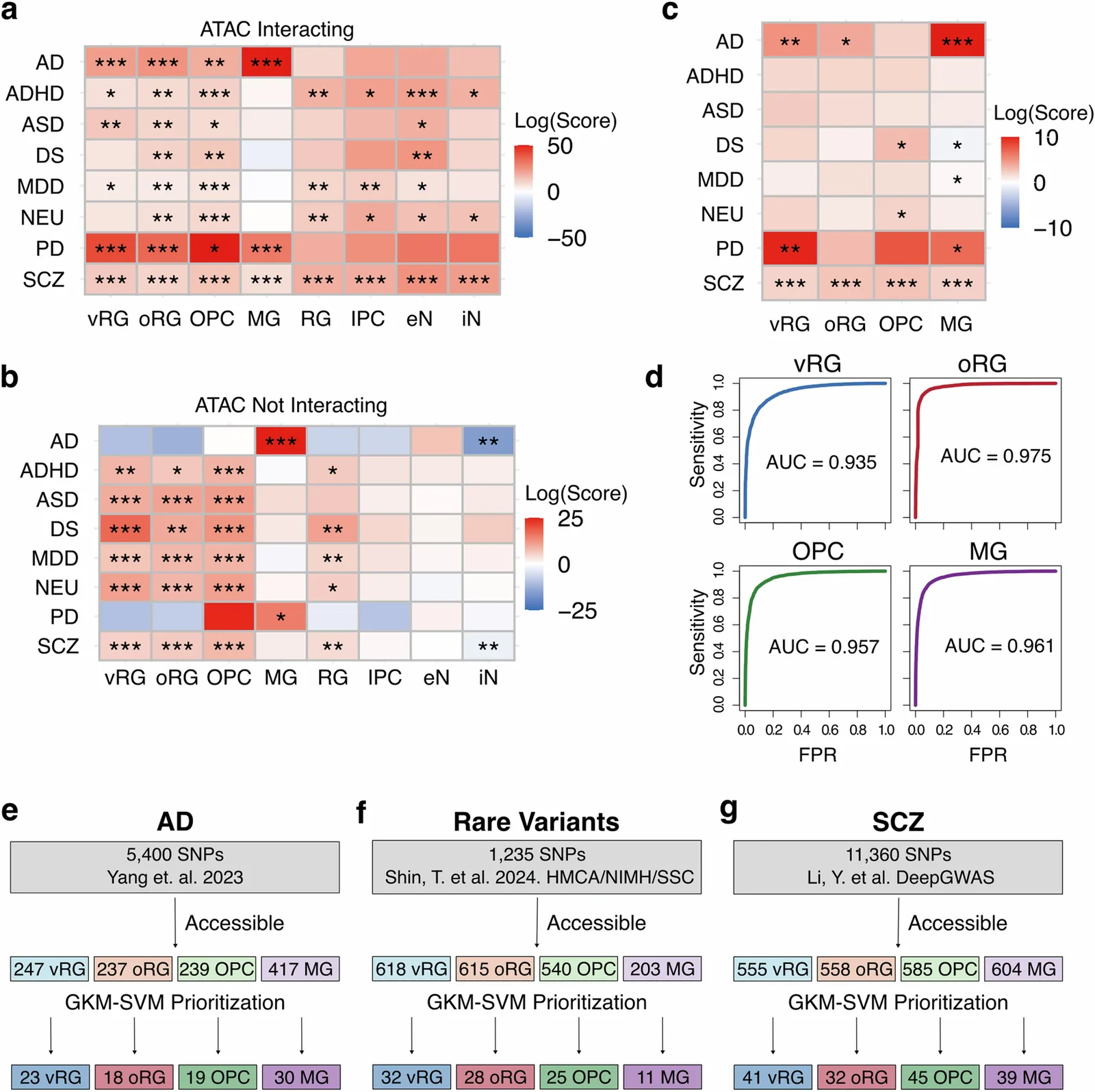
gkm-SVM model predicts accessibility and evaluates neuropsychiatric variants. **a-c**) Heatmap of LDSC score for (a) ATAC-seq peaks involved in 3D interaction (b) ATAC-seq peaks not involved in 3D interactions, (c) distal interacting 2 kb bins. Red, the disorder is positively enriched. Blue, the disorder is negatively enriched (two-sided LDSC enrichment p-values *: *P* < 0.05, **: *P* < 0.01, ***: *P* < 0.001). **d**) Area under the receiver operator characteristic curve (AUC) results of gkm-SVM training. **e-g**) Schematic of variant prioritization for (e) AD (f) Rare Variants (g) SCZ. 1) Credible SNPs accessible in each cell type. 2) SNPs predicted to significantly disrupt accessibility compared to shuffled controls.

To assist with interpreting the heritability of GWAS variants and parse cell type contribution to disease, we performed linkage disequilibrium score regression (LDSC) on interacting cCREs with the GWAS summary statistics for Alzheimer’s disease^37^, attention deficit hyperactivity disorder^38^, autism spectrum disorder (ASD)^39^, depressive symptoms^40^, major depressive disorder^41^, neuroticism^39^, Parkinson’s disease^42^ and schizophrenia (SCZ)^43^ (Fig. 4a). MG cCREs within the developing brain are the only cell type enriched for Alzheimer’s disease heritability, consistent with Alzheimer’s disease heritability enrichment at cCRE of MG from adult tissues^44^. Moreover, vRG, oRG and OPC cCREs are enriched in attention deficit hyperactivity disorder and SCZ, whereas only vRG and oRG were enriched for ASD. LDSC analysis on all interacting 2-kb bins rather than interacting cCREs revealed lower enrichment for the heritability of neuropsychiatric disorders, highlighting the importance of using epigenomic signals for enrichment analysis (Extended Data Fig. 7c). We further expanded this analysis by including chromatin accessible regions from this study along with those from neurons^3^. Accessible regions participating in 3D interactions were enriched for neuropsychiatric traits in both glial and neuronal cell types (Extended Data Fig. 7a), whereas non-interacting accessible regions were exclusively enriched for neuropsychiatric traits in glial cell types, albeit at a lower signal (Extended Data Fig. 7b).

**Extended Data Fig. 8:**
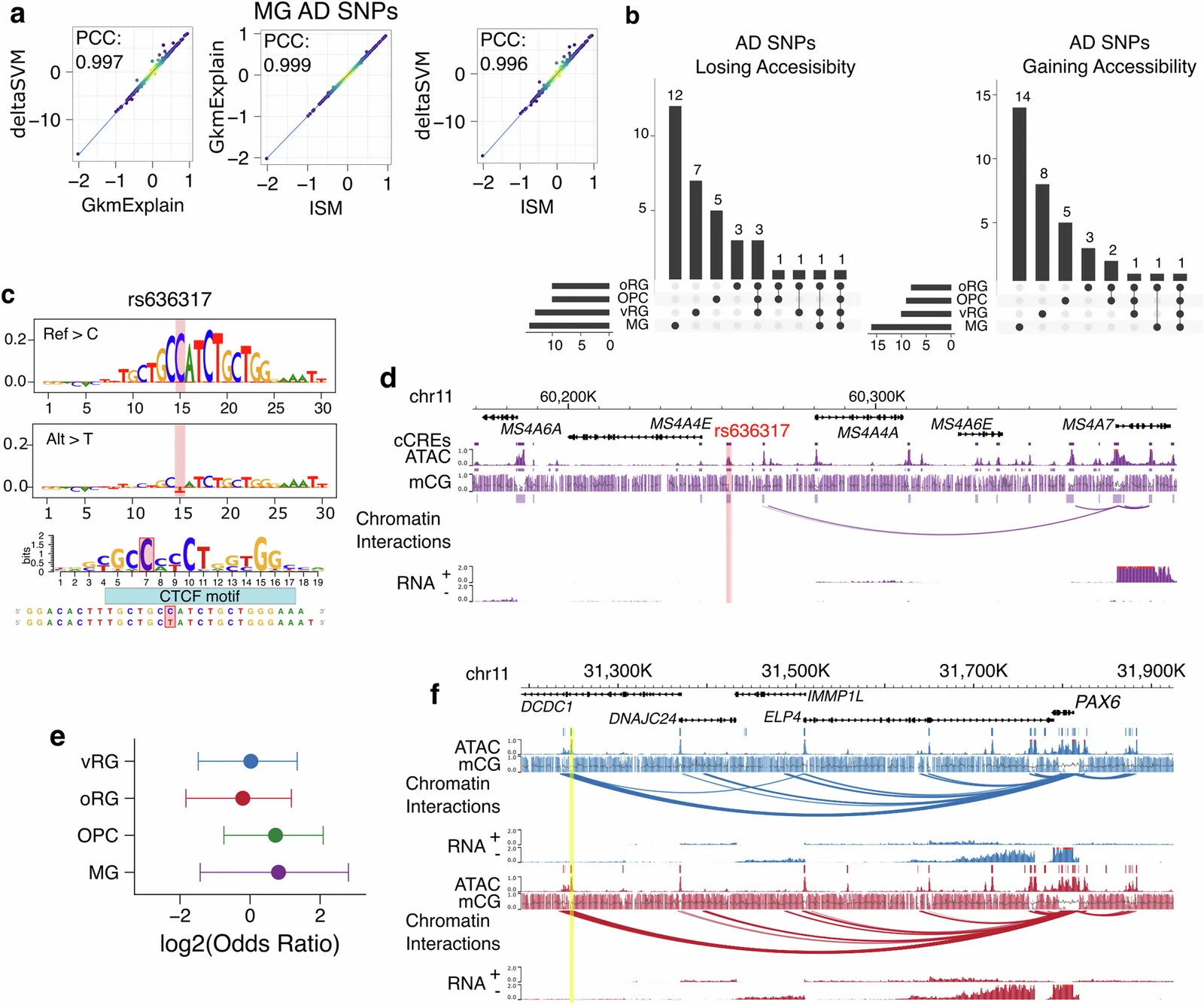
Prioritized AD and rare variants. **a**) Scatterplots showing the Pearson correlation coefficient of deltaSVM, ISM, and GkmExplain MG predictions for AD SNPs. **b**) (Left) Upset plot of AD SNPs predicted to lose accessibility in silico*.* (Right) Upset plot of AD SNPs predicted to gain accessibility in silico. **c**) Top, GkmExplain scores with the MG model for rs636317 SNP with the non-risk C allele. Middle, GkmExplain scores with the MG model for rs636317 SNP with the risk T allele. Bottom, motifbreakR prediction of the disruption of the CTCF motif. **d**) WashU browser of the locus containing rs636317 SNP, ATAC peak highlighted in red. MG tracks shown. **e**) Forest plot showing the association of rare variants with ASD cases. Each point represents the log2 odds ratio, with the error bars indicating 95% confidence intervals (n = 676 total genomic regions tested). **f**) WashU browser of the locus containing HAR0366.

Determining which GWAS single nucleotide polymorphisms (SNPs) are functional is challenging as SNPs largely reside in the non-coding genome and show cell type heterogeneity. To overcome these challenges and prioritize SNPs, we evaluated in silico perturbations of chromatin accessibility resulting from credible SNP variants for each cell type. First, we trained a gapped *k*-mer support vector model (GKM-SVM) to predict cell-type-specific accessibility by DNA sequence (Extended Data Fig. 7d, Supplementary Table 8 and Methods). Second, accessible variants were further assessed for their potential to perturb chromatin accessibility using three techniques, deltaSVM^45^, in silico mutagenesis (ISM) and GkmExplain^46^. Out of 5,400 credible Alzheimer’s disease variants^47^, 565 were accessible in at least 1 cell type, among which 68 were predicted to perturb accessibility, with MG having the most variants (Extended Data Figs. 7e and 8a,b and Supplementary Table 9). The top variant across all four cell types, rs636317, was predicted to decrease accessibility when the risk allele was present (Extended Data Fig. 8c,d). Correspondingly, the risk allele of rs636317 has been proposed to disrupt CTCF binding^48,49^ and is a strong eQTL for *MS4A6A* in monocytes in which it increases *MS4A6A* expression^49^.

We performed a similar analysis with previously identified non-coding rare variants from several ASD cohorts overlapping with HARs, VISTA enhancers or conserved regions predicted to act as neural enhancers^50^. Out of 1,235 rare variants, 723 were accessible in at least 1 of the cell types, with 61 predicted to perturb accessibility (Extended Data Fig. 7f). The variants were found in cases and controls (167 case-specific, 509 control-specific and 47 shared) and were predicted to affect accessibility to a similar extent across all cell types, indicating no global difference in regulatory impact between cases and controls (Extended Data Fig. 8e). Instead, this analysis resolves case-associated variants with cell-type-specific effects, such as a case variant in HAR0366 (chromosome (chr.) 11:31246972:C>T) predicted to disrupt accessibility in vRG and oRG. HAR0366 also is linked through chromatin interactions with the ASD-associated TF PAX6^51^ (Extended Data Fig. 8f).

**Extended Data Fig. 9:**
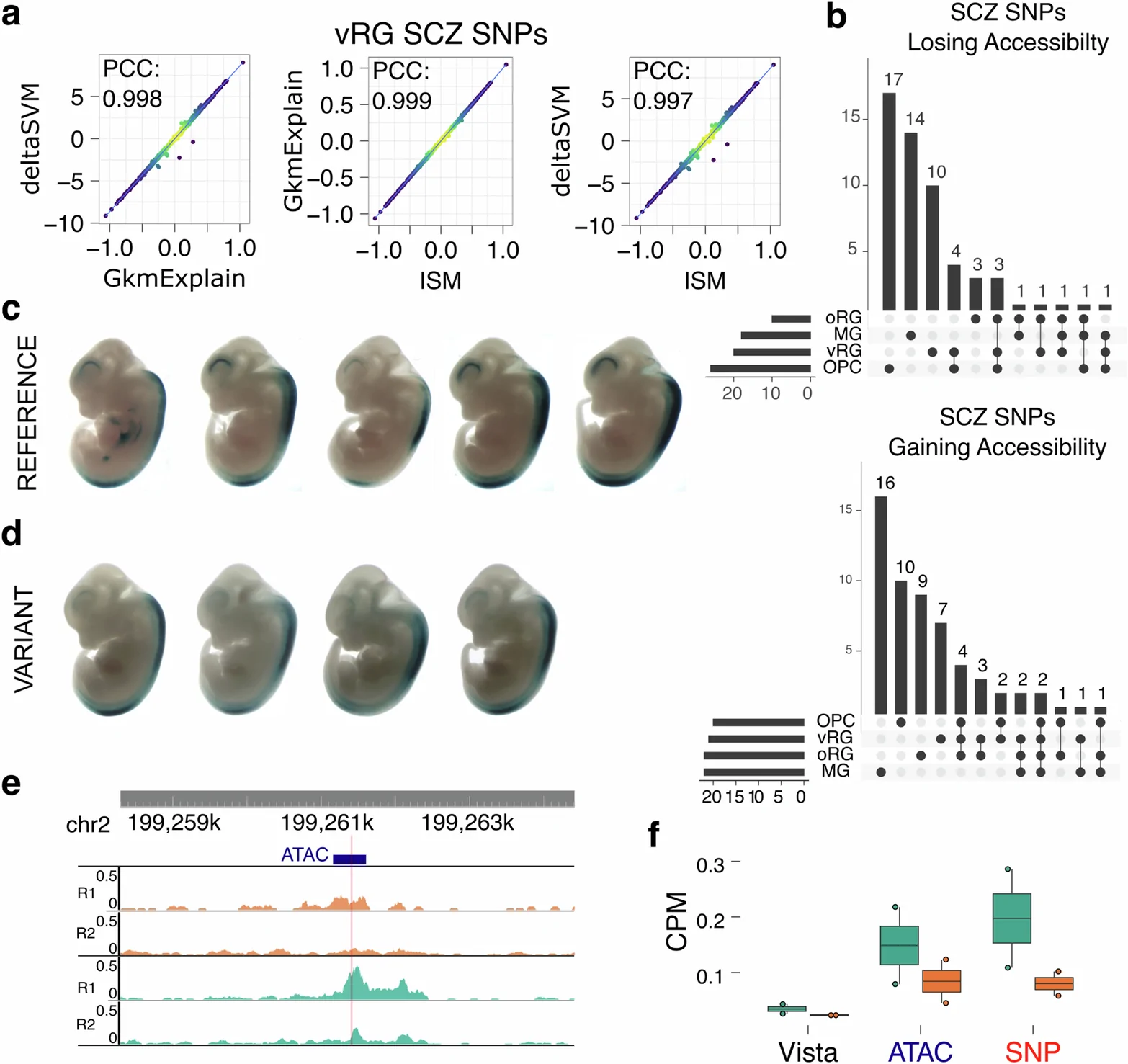
Prioritized variants for SCZ. **a**) Scatterplots showing the Pearson correlation coefficient of deltaSVM, ISM, and GkmExplain vRG predictions for SCZ SNPs. **b**) (Top) Upset plot of SCZ SNPs predicted to lose accessibility in silico. (Bottom) Upset plot of SCZ SNPs predicted to gain accessibility in silico. **c**) All VISTA results at e12.5 for rs4449074 wild-type allele with 4/5 displaying forebrain activity **d**) All VISTA results at e12.5 for rs4449074 risk allele with 4/4 lower signal in the forebrain. **e**) WashU browser of the VISTA element (chr2:199258295-199264414) containing rs4449074 showing vRG ATAC-seq signal for each replicate (C/T = 2, C/C = 2). **f**) Box plot of average atac-seq signal across the total VISTA element chr2:199258295-199264414 (black), ATAC-seq peak chr2:199261161-199261604 (blue), and rs4449074 (red). Light blue is homozygous C/C. Orange is heterozygous (C/T). Box boundaries represent the Q1 and Q3 quartiles with the median indicated by the central line. Whiskers extend to the minimum and maximum values, and outliers are shown as individual points (n = 2 per genotype).

We further performed in silico testing on SCZ variants, because previous studies have highlighted SCZ risk genes associated with lineage-specific gene signatures in the second trimester cortex^52^. Of the 11,360 SCZ variants prioritized in DeepGWAS^53^, 929 were accessible and 112 were predicted to perturb accessibility in at least 1 cell type (Extended Data Fig. 7g and Extended Data Fig. 9a,b). One SNP, rs4449074, overlaps with a VISTA enhancer, hs3134, that was previously reported to have neural activity by the transgenic mouse assay and is predicted to lose accessibility with the risk allele (Fig. 4b,c). Accordingly, vRG chromatin accessibility was lower in two samples heterozygous at rs4449074 compared with two individuals homozygous for the non-risk allele, although the results were not statistically significant (Extended Data Fig. 9e,f). Furthermore, the hs3134 activity of the risk allele T in the forebrain was reduced compared with the non-risk allele C in transgenic mouse assay in vivo (Fig. 4c and Extended Data Fig. 9c,d).

## oRG cCREs are enriched for HARs

**Fig. 5:**
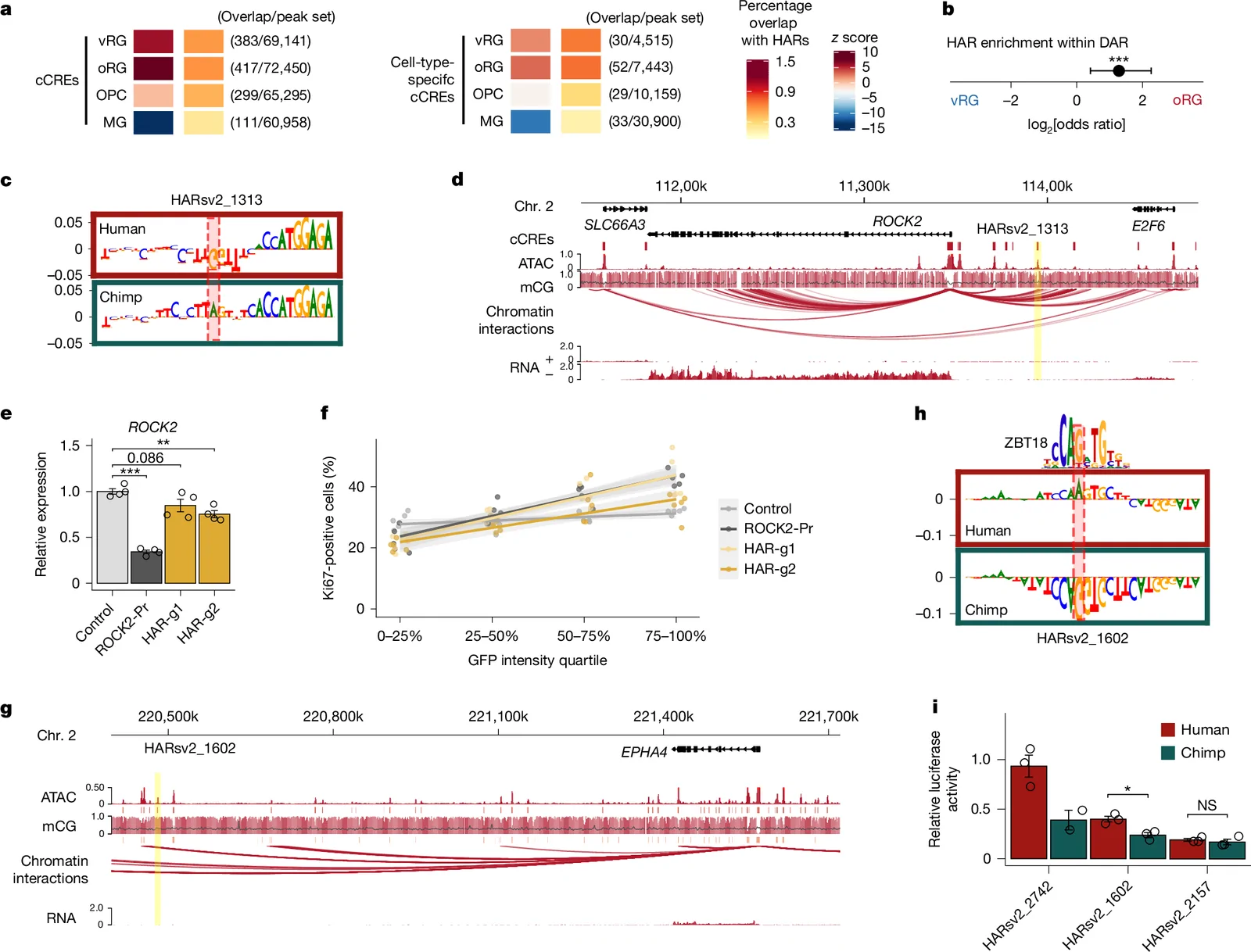
oRG cCREs are enriched for HARs. **a**, Heatmap showing the *z* score (left) for overlap of either cCREs or cell-type-specific cCREs compared with a randomly sampled empirical null distribution as well as the percentage of HARs overlapping the data (right). **b**, Forest plot showing the association of vRG (*n* = 5,321) or oRG DARs (*n* = 2,320) with HARs. The point represents the log_2_ odds ratio, with the error bars indicating 95% confidence intervals (two-sided Fisher’s exact test, ***P < 0.001).** c**, Overview of HARsv2_1313. Top, GkmExplain importance scores for each base pair surrounding the human G allele. Bottom, GkmExplain importance scores for each base pair surrounding the chimpanzee ancestral A allele. **d**, WashU browser of the *ROCK2* locus. HARsv2_1313 highlighted in yellow. **e**, Expression of *ROCK2* in NPC based on quantitative PCR with reverse transcription. Four independent differentiations per condition were used (two-sided *t*-test, *P < 0.05, **P < 0.01, ***P < 0.001). Data are presented as mean values ± s.e.m. **f**, Percentage of cells positive for Ki67^+^ in NPC on the basis of immunocytochemistry stratified into quartiles by GFP intensity. Linear regression shows the mean with 95% confidence intervals. **g**, WashU browser of the *EPHA4* locus. HARsv2_1602 highlighted in yellow. **h**, Overview of HARsv2_1602. Top, ZBT18 motif that is predicted to be perturbed. Middle, GkmExplain importance scores for each base pair surrounding the human A allele. Bottom, GkmExplain importance scores for each base pair surrounding the chimpanzee ancestral G allele. **i**, Relative luciferase activities compared with minimum promoter of HARs predicted to be more accessible than chimp orthologues (two-sided *t*-test, NS, not significant, *P < 0.05). Data are presented as mean ± s.e.m. (error bars) from *n* = 3 independent experiments, except HARsv2_2742 chimpanzee sequence (*n* = 2).

**Extended Data Fig. 10:**
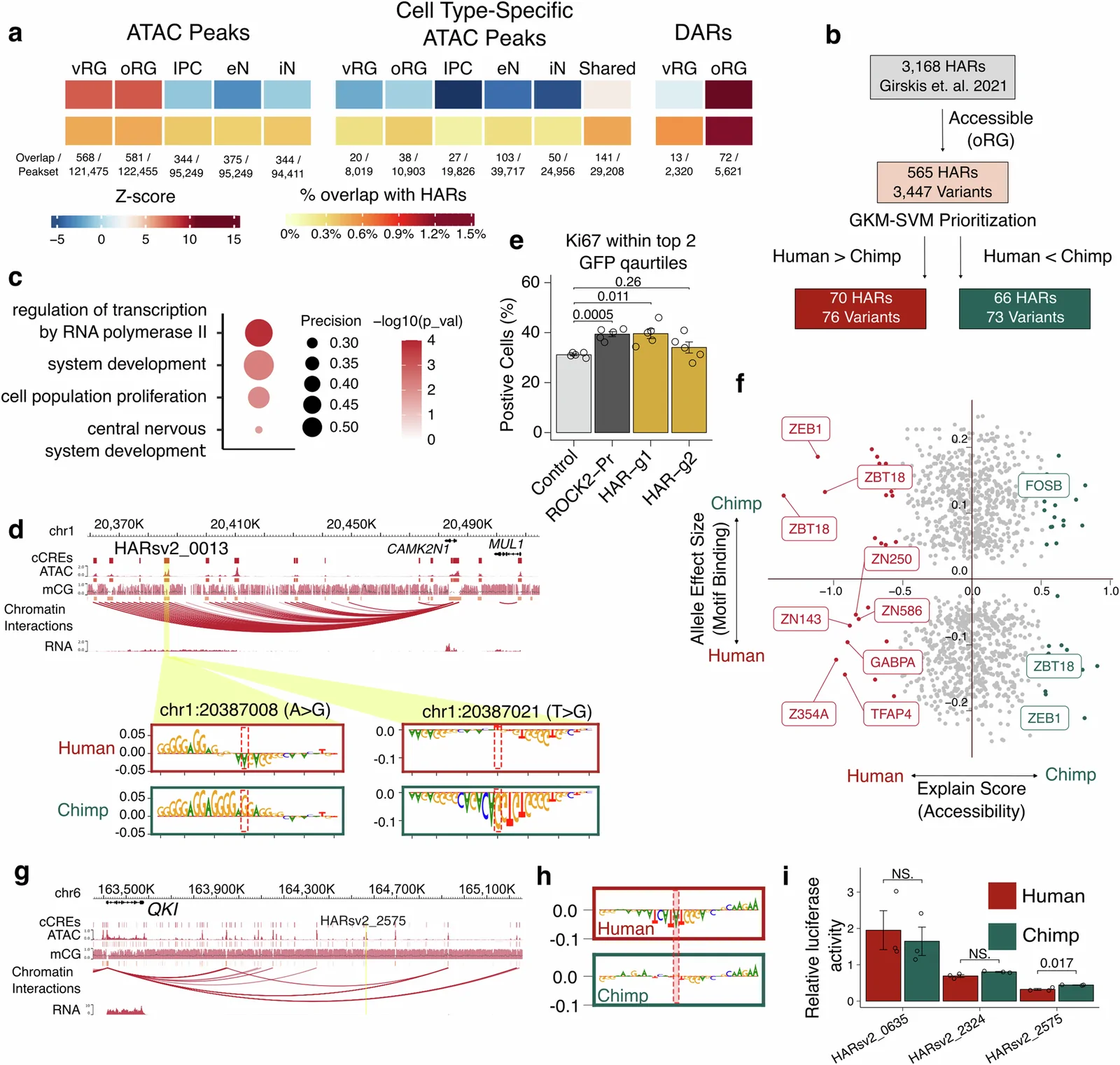
Enrichment of HARs within the developing cortex and GKM prediction of HAR variants between human and chimpanzee. **a**) Heatmap displaying the z-score for overlap of either ATAC peaks, cell type-specific ATAC Peaks, or vRG and oRG DARs compared with a randomly sampled empirical null distribution as well as the percentage of HARs overlapping the data. **b**) Schematic of prioritization of HAR variants. 1) HARs accessible in oRG. 2) Variants predicted to significantly disrupt accessibility compared to shuffled controls. **c**) Gene ontology results of HARs (n = 45) predicted to alter accessibility. **d**) Top, WashU browser of the locus containing HARsv2_0013. Bottom, GkmExplain scores with the oRG model for chr1:20387008:A:G and chr1:20387021:T:G. **e**) Percentage of cells positive for Ki67+ in NPC based on immunocytochemistry. Five independent differentiations per condition were used (two-sided t-test). Data are presented as mean values ± s.e.m. **f**) Scatter plot of HAR variants between human and chimpanzee. X-axis, GkmExplain score changes, with negative scores meaning more accessible in humans and positive scores more accessible in chimpanzees. Red dots are significant compared to matched null control (*P* < = 0.05); gray dots are not significant. Y-axis, allele scores difference obtained from motifbreakR, with negative scores meaning more likely bound in humans and positive more likely bound in chimpanzees. **g**) WashU browser of the *QKI* locus. HARv2_2575 highlighted in yellow. **h**) Overview of HARsv_2575. Top, GkmExplain importance scores for each base pair surrounding the human A allele. Bottom, GkmExplain importance scores for each base pair surrounding the chimpanzee ancestral G allele. **i**) Relative luciferase activities compared to minimal promoter of HARs predicted to be less accessible than chimp orthologs (two-sided t-test). Data are presented as mean values ± s.e.m. (n = 3 independent experiments).

HARs can act as neurodevelopmental enhancers^54–56^ and recent reports highlight oRG as a potential cell type in which HARs could be functional on the basis of expression of assigned target genes^57^. We found both vRG and oRG cCREs enriched for 3,168 annotated HARs^58^ relative to randomly sampled cCREs compiled across all glial cell types (Fig. 5a). In addition, RG DARs further highlighted that oRG cCREs are associated with HARs, because 72 (1.35%) oRG DARs overlap HARs compared with 13 (0.56%) vRG DARs (odds ratio 2.43, *P* = 0.00187) (Fig. 5b). On expanding this analysis with ATAC-seq peaks in IPC, eN and iN^3^ to include most cell types within this developmental stage, oRG cCREs enrichment of HARs remains (Extended Data Fig. 10a).

To understand whether HARs could alter enhancer function compared with their chimpanzee orthologues, we leveraged the previous GKM-SVM model trained on oRG accessibility to identify variations leading to the greatest in silico changes. In total, we evaluated 565 accessible HARs in oRG, containing 3,447 variants relative to chimpanzees. Within 70 HARs, 76 variants were predicted to increase chromatin accessibility in HARs, whereas 73 variants within 66 HARs were predicted to be more accessible in chimpanzee orthologues (Extended Data Fig. 10b and Supplementary Table 10). Gene ontology analysis of target genes for prioritized HARs included terms such as ‘system development’ and ‘cell population proliferation’, suggesting their potential roles in human-specific cortical expansion (Extended Data Fig. 10c). Consistent with previous models that discovered diverging variants within the same HAR^58^, we identify eight HARs with variations affecting chromatin accessibility in opposing directions. For example, in HARsv2_0013 the human allele A at chr. 1:20387007 was predicted to reduce chromatin accessibility compared with the ancestral G allele, whereas the human allele T at chr. 1:20387020 was predicted to increase chromatin accessibility relative to the ancestral G allele (Extended Data Fig. 10d).

We further investigated the function of HARsv2_1313, for which the human-specific G allele at chr. 2:11,391,810 is predicted to reduce chromatin accessibility relative to the ancestral A allele in oRGs (Fig. 5c). HARsv2_1313 interacts with the promoter of *ROCK2* (Fig. 5d). CRISPR interference (CRISPRi) using paired guide RNAs (gRNAs) (Supplementary Table 10) in human induced pluripotent stem (iPS) cell-derived neuronal precursor cells (NPCs) demonstrated decreased *ROCK2* expression, confirming that HARsv2_1313 functions as an enhancer of *ROCK2* (Fig. 5e). Consistent with previous evidence that ROCK inhibition promotes stem-cell proliferation and viability^59^, we observed a significant increase in Ki67-positive cells in EMX + NPC population following knockdown of the *ROCK2* promoter and HARsv2_1313 with one pair of gRNAs, whereas the second pair showed a similar trend towards increased proliferation (Extended Data Fig. 10e). Moreover, the percentage of Ki67-positive cells was positively correlated with the gRNA-associated green fluorescent protein (GFP) signal in all conditions relative to control (slope difference 3.48–5.86, *P* < 0.001) (Fig. 5f). We note that technical considerations, including incomplete transduction efficiency and potential indirect effects on other pathways, may contribute to variability in the observed relationship between *ROCK2* mRNA levels and proliferation. Nevertheless, our findings support HARsv2_1313 as a regulator of *ROCK2* expression and suggest that perturbation of this regulatory element is associated with increased proliferation, although the precise mechanism linking these observations remains to be fully established. These observations are also consistent with previous findings that CRISPR activation of this HAR increased *ROCK2* expression in iPS cell-derived neurons^60^ and that *ROCK2* expression is higher in chimpanzee organoids compared with human organoids^7^.

As TF binding often alters chromatin accessibility, we integrated motif disruption predictions from motifbreakR^61^ and found that disruption in known activator and repressor TF motifs correlates with corresponding accessibility changes (Extended Data Fig. 10f). For example, predicted binding of repressors ZEB1 and ZBT18^62,63^ due to variants within HARs are associated with congruent predictions of decreased accessibility, whereas binding of activators ZNF143 and FOSB correlates with increased accessibility^64^. Of the 69 HARs overlapping oRG DARs, 11 HARs contain variants that are predicted to perturb chromatin accessibility, 9 of which are also predicted to disrupt binding motifs. One example that predicted the greatest change in accessibility, HARsv2_1602, is located within a gene desert greater than 1 Mb away from the *EPHA4* promoter, consists of a nucleotide change of the human-specific allele A from the ancestral G, leading to the predicted loss of the repressor ZBT18 TFBS and increased chromatin accessibility (Fig. 5g,h). EPHA4, a receptor tyrosine kinase, has a key role in neuronal development^65^, regulates the size of the neonatal cortex in mice^66^ and is more highly expressed in human RG compared with macaque RG^7^.

To further validate our in silico predictions, we tested the regulatory activities of six regions surrounding prioritized HAR variants predicted to alter TFBS and that overlapped oRG DARs in primary RG isolated from the second trimester cortical tissues. Five of the regions had regulatory activities less than the minimum promoter, consistent with the predicted repressive TFBS such as ZBT18, ZEB1 and SMCA5. Two of the three regions surrounding variants that were predicted to be more accessible than their chimpanzee orthologues, in HARsv2_1602 and HARsv2_2742, demonstrated at least a 50% increase in regulatory activity on relative luciferase reporter expression (Fig. 5g–i). The other region in HARsV2_2157 showed the expected trend of increased activity, but only modestly, with an 18.28% increase (Fig. 5i and Supplementary Table 11). Among the remaining three HAR variants (within HARsv2_0635, HARsv2_2575 and HARsv2_2324) predicted to be less accessible than their chimpanzee orthologues, the chimpanzee orthologue of HARsv2_2575 showed a significant increase in relative luciferase activity and the chimpanzee orthologue of HARsv2_2324 has 15.69% more relative luciferase activity than HARsv2_2324, approaching significance (*P* < 0.1) (Extended Data Fig. 10i). *QKI*, the gene neighbouring HARsv2_2575 (Extended Data Fig. 10g,h), is upregulated in IPC and eN within chimpanzee organoids^7^ as well as chimpanzee iPS cell-derived eNs^56^ compared with their human counterparts. Moreover, HARsv2_2575 is accessible in chimpanzee iPS cell-derived eNs, but not human iPS cell-derived eNs^55^, consistent with our prediction of greater accessibility in the chimpanzee orthologue of HARsv2_2575. Overall, by leveraging epigenomic data, we show that oRG cCREs are enriched for HARs and provide putative target genes of prioritized HARs.

## Discussion

In this study, we extensively characterized four glial cell types in developing human cortex and identified more than 60,000 cCREs for each cell type. Moreover, we leveraged 3D interactions to assign target genes to cCREs at a resolution higher than either previous bulk or single-cell 3D epigenomic assays^3,10^. We confirmed enhancer activity in 11 out of 15 RG cCREs with mouse transgenic assays and further associated cCREs with neural enhancer activity when expanding to all VISTA enhancers^24,25^. We systematically compared two subtypes of neuronal progenitors, vRGs and oRGs, known to contribute to cortical expansion and identified LHX2 and ASCL1 as key TFs enriched in cell-type-specific cCREs. Notably, LHX2 can inhibit astrogenesis within the hippocampus, suggesting that it may promote proliferation and neurogenesis within oRGs^36^, whereas ASCL1 is implicated in driving neuronal lineages as well as modulating the number and distribution of derived glial cells^67,68^. Although TFBS were filtered for highly expressed TFs, it is still difficult to distinguish between motif family members such as TCF4 and TCF12.

Our high-resolution datasets also facilitated the prioritization of disease-associated SNPs with machine learning models, allowing us to successfully identify the known Alzheimer’s disease SNP rs636317 (ref. ^49^), highlight cell-type-specific effects of rare non-coding variants^50^ and further prioritize the SCZ variant rs4449074. We validated the perturbation of enhancer activity by the rs4449074 risk allele using transgenic mouse assays, underlining how high-resolution epigenomic annotation combined with machine learning can assist in the identification of functional variants from thousands of GWAS-identified variants. This technique has also been successfully deployed in adult brain tissues^69^ and follicle development^70^. Whereas in vitro systems are an extremely valuable resource, building models to predict variant function on the basis of data from primary tissue is essential to understanding the impact of variation given differences between iPS cell-derived systems, organoids and primary tissue^7,71^. However, further validation and mechanistic studies are still necessary to confirm model predictions and interpret the role of variants within diseases.

Although previous studies proposed that HARs are associated with neurodevelopment^54–57^, we recapitulate the enrichment of HARs within oRGs from the second trimester cortex and provide further regulatory targets of HARs with our 3D epigenomic data. In contrast to previous work leveraging massively parallel reporter assay^60^ or epigenomic characterization within neuronal progenitor cells^72^, we incorporated oRG chromatin accessibility to train our model and predict functional HARs in silico. Validation of one prediction, HARsv2_1313, indicates that this element regulates *ROCK2* expression, potentially linking this element to the control of cell proliferation, as reflected by Ki67 staining. Furthermore, we find that TFBS disruption by HAR variants complements our GKM-SVM model as there are many cases in which predictions of lower accessibility correspond with the insertion of a ZEB1 TFBS, a repressor known to influence neuronal differentiation^62^. As oRGs largely reside within large mammalian cortices, prioritized HARs may contribute to human-specific cortical expansion as suggested by proliferative terms associated with HAR target genes.

In conclusion, by conducting bulk assays of cell types isolated by FACS, we obtained high-quality data with improved resolution compared with previous results^11,12^. However, we are limited to analysing average signals across dynamic cell populations, and additional rare cell subtypes identified by single-cell omic techniques such as Tri-IPCs are not characterized^2^. In the future, as single-cell approaches continue to improve, our annotation can provide direction for cCREs, lineage TFs and genes of interest to be accessed in spatial^73^ or lineage^74^ contexts to unravel cortical development in organoids or tissues.

## Methods

### Tissue dissociation, sample fixation and storage

Cells were isolated from the developing human cortex between GW15 and GW24 using a method similar to that previously described in ref. ^3^. Dissociated cells were washed twice in PBS and fixed in 2% PFA for 10 min at room temperature. Fixation was quenched by adding glycine to a final concentration of 200 mM, followed by incubation for 5 min at room temperature. From this point onwards, all procedures were performed either on ice or at 4 °C. Fixed cells were pelleted by centrifugation at 1,000*g*, washed once with PBS, filtered through a 70-μm nylon mesh and washed once again with PBS. Finally, the cells were pelleted and stored at −80 °C.

### FACS

All procedures were performed either on ice or at 4 °C. About 1.5 × 10^8^ fixed cells were thawed and permeabilized by incubating in 1 ml of PBS containing 0.1% Triton X-100 for 15 min. Bovine serum albumin (BSA) was added to a final concentration of 1% and cells were pelleted by centrifugation at 1,000*g* for 8 min. Cells were washed once in staining buffers (PBS with 1% BSA) and resuspended in 100 µl of staining buffer. Cells were blocked by FcR Blocking Reagent (Miltenyi Biotech, 1:20) for 10 min, followed by antibody incubation for 30 min. The antibodies used for FACS included PerCP-Cy5.5 anti-SOX2 (BD Biosciences, 561506, for RG), PE-Cy7 anti-EOMES (Invitrogen, 25-4877-42, for RG), unconjugated anti-HOPX (Proteintech, 11419-1-AP, for RG), Alexa Fluor 647 anti-OLIG2 (Abcam, ab225100, for OPC/MG) and PE anti-PU.1 (Cell Signaling Technology, 81886, for OPC/MG). The HOPX antibody was used at 1:250 dilution, whereas all other antibodies were used at 1:20 dilution. After incubation, cells were washed twice in staining buffer, resuspended in 300 µl of staining buffer and incubated with the Alexa Fluor 647 donkey anti-rabbit secondary antibody (Invitrogen, A-31573, for RG FACS only) at 1:300 dilution. Cells were sorted using BD FACSAria II sorters into collection buffer (PBS with 5% BSA) (Supplementary Figs. 1 and 2). Sorted cells were pelleted by centrifugation at 1,000*g* for 10 min, snap-frozen on dry ice and stored at −80 °C before further processing. For FACS performed for RNA-seq, 1% RiboLock Rnase Inhibitor (Thermo Scientific, EO0384) was included in all buffers.

### RNA-seq library creation and analysis

We extracted total RNA from the sorted cell populations using the RNA FFPE kit (Qiagen 73504) starting with 3 × 10^5^ to 1.8 × 10^6^ cells. The quality of the extracted RNA was checked by determining the percentage of RNA fragments with size larger than 200 bp (DV200) from the Agilent 2100 Bioanalyzer, with DV200 ≥ 30% used for library construction. Samples were then depleted of ribosomal RNA using the KAPA RNA HyperPrep Kit with RiboErase (HMR KK8560) and we performed first and second strand synthesis, dA-tailing and sequencing adapter ligation. Last, sequencing adapters were added by means of PCR amplification and libraries were sent for paired-end sequencing on the NovaSeq S4 instrument (100 bp paired-end reads).

Raw reads were trimmed to 100 bp using fastp^75^ (v.0.22.0) and then aligned to hg38 using STAR (v.2.7.10a) running the standard ENCODE parameters. Strand-specific quantification was performed using RSEM (v.1.2.28) with the GENCODE 38 annotation. Library quality was further evaluated with median TIN^76^ score and shown to be greater than 60 across all samples. TMM-normalized reads per kilobase per million mapped reads (RPKM) values for each gene were obtained by use of the edgeR^77^ (v.3.32.1) package. The mean values across all replicates were used for all downstream analyses. DEGs were identified with DESeq2^78^ using a multi-factor design including the genotype or individual to identify differences between vRG and oRG. Clustering was performed on regularized log transformation data and hierarchical clustering based on sample distances after removing batch effects from genotype or individual with the removeBatchEffect command from limma^79^ (v.3.46.0).

### Characterizing bulk RNA-seq with scRNA-seq

We leveraged CIBERSORTx^80^ to characterize cell composition with matching scRNA-seq^9^. eN, iN and IPC subclusters were first combined into one cluster before creating the reference matrix. Final counts consisted of eN, iN, IPC, vRG, oRG, truncated RG, OPC and MG cluster. Raw counts of bulk vRG, oRG, OPC and MG libraries from this study were obtained with tximport() from the rsem output. Finally, CIBERSORTx was used to impute cell fractions with the following parameters, batch correction B-mode, relative run-mode and 100 permutations.

### ATAC-seq library creation and analysis

ATAC-seq was conducted in a similar way to that previously described in ref. ^3^. In brief, 50,000–100,000 formaldehyde fixed and sorted cells were resuspended in nuclei extraction buffer (10 mM Tris-HCl pH 7.5, 10 mM NaCl, 3 mM MgCl_2_, 0.1% Igepal CA630 and 1× protease inhibitor) at 4 °C for 5 min. Next, cells were resuspended in 50 μl of 1× TD buffer from Nextera DNA Library Prep Kit (Illumina FC-121–1030) and incubated with 2.5 μl of TDE1 enzyme for 45 min at 37 °C with 4,500 rpm. Afterwards, 150 μl of reverse crosslinking solution (50 μl of 1 M Tris pH 8.0, 100 μl of 10% SDS, 2 μl of 0.5 M EDTA, 10 μl of 5 M NaCl, 800 μl of water and 2.5 μl of 20 mg ml^−1^ proteinase K) was added and incubated at 65 °C overnight. DNA was purified using Qiagen MinElute kit (28004), PCR amplified and last size-selected for fragments between 300 bp and 1,000 bp with AMPure beads (Beckman Coulter, wsr-450437). Libraries were sequenced on the NovaSeq S4 instrument (100 bp paired-end reads). Raw reads were trimmed to 100 bp using fastp^75^ (v.0.22.0), mapped to hg38 and processed using the ENCODE pipeline (https://github.com/kundajelab/atac_dnase_pipelines) running the default settings. We achieve transcriptional start site enrichment scores greater than 7 (18.54–27.69) and fractions of reads within peaks greater than 0.3 (0.32–0.53) for all replicates. For each cell type, optimal overlapping peaks were used for all downstream analysis. DARs of vRG and oRG were obtained with DiffBind^81^ (v.3.4.11) using DESeq2 with a design as previously described to obtain DEGs and a cut-off at a false discovery rate of less than 0.01, after obtaining a consensus peak set and data normalization. ATAC-seq clustering was performed with a Spearman correlation of reads within a merge peak set across all four cell types using the multiBamSummary from deepTools^82^ (v.3.5.1).

### WGBS library creation and analysis

DNA was isolated from sorted cell populations using the MagMAX FFPE DNA/RNA Ultra kit (Applied Biosciences, A31879) starting with 2.9 × 10^5^ to 1.5 × 10^6^ cells. Isolated genomic DNA was sonicated to 300–600 bp using a Covaris M220 and size confirmed using the Agilent BioAnalyzer. 0.5% of unmethylated lambda DNA was added to each sample to control for bisulfite conversion. Bisulfite conversion was performed using the EZ DNA Methylation Direct kit (Zymo, D5020). WGBS libraries were constructed using the Accel-NGS Methyl-seq Combinatorial Dual Indexing kit (Swift Biosciences, 38096). Library size and adapter removal were confirmed using the Agilent BioAnalyzer. Libraries were paired-end sequenced on the NovaSeq instrument (100 bp and 150 bp paired-end reads).

Fastq files for paired-end WGBS samples were trimmed for Illumina adapter sequences with an extra 15 bases removed from the 5′ end of read 2 and the 3′ end of read 1 using TrimGalore v.0.6.6 (https://github.com/FelixKrueger/TrimGalore). We used FastQC to check the quality of the raw and trimmed FASTQ files and ensure methylation biases from end repair were removed. Trimmed FASTQ files were mapped using Bismark^83^ v.0.16.2 with Bowtie2 (ref. ^84^) v.2.4.1 to genome build hg38. We used the deduplicate_bismark tool to remove duplicate reads from each sample before merging replicates of the same cell type. Unmethylated lambda DNA was spiked-in to each sample before bisulfite treatment and library prep. We mapped reads to lambda DNA genome to confirm greater than 99% bisulfite conversion efficiency of each sample. After merging replicates, methylation calls for each C context were determined using bismark_methylation_extractor. Only Cs with at least ten times coverage were used for downstream analysis.

We used methylKit^85^ to identify DMRs in a pairwise manner. DMRs were considered differentially methylated if there was at least a 25% methylation difference. We used MethylSeekR^86^ to identify unmethylated regions, LMRs and partially methylated domains.

### PLAC-seq library creation and analysis

PLAC-seq was performed using the Arima-HiC+ Kit. Briefly, 2 to 4 million cells fixed with 2% formaldehyde (F79-500) were used to prepare each library. Following digestion and ligation the chromatin was sonicated with the following parameters using the Covaris S220 instrument: setpoint temperature was 4 °C, peak power was 105 W, duty factor was 5%, cycles per burst were 200 and treatment time was 300 s. Immunoprecipitation was performed using 2.5 μl of the H3K4me3 antibody (Millipore, 04-745). Sequencing adapters were added with Swift Biosciences Accel-NGS 2S plus DNA library kit and amplified with KAPA HiFi HotStart ReadyMix. Libraries were sent for paired-end sequencing on the NovaSeq S4 instruments (100 bp paired-end reads). Raw reads were trimmed to 100 bp using fastp^75^ (version 0.22.0).

We used the MAPS^87^ pipeline to call significant H3K4me3-mediated chromatin interactions at a resolution of 2 kb and range of 2 Mb on the basis of our PLAC-seq data. First, BWA-MEM was used to map raw reads to hg38. Unmapped reads and reads with low mapping quality were discarded, and the resulting read pairs were processed as previously reported. After mapping, read pairs were classified as AND, XOR or NOT interactions on the basis of whether both, one or neither of the pairs overlapped the universal anchor. To obtain the universal anchor bins, we first identified H3K4me3 peaks for each cell type with MACS2 using the options ‘-g hs --broad --nolambda --broad-cutoff 0.01’ for roughly 30 million read pairs with interaction distance shorter than 1 kb in each cell type. This resulted in 19,826 (37,241), 18,198 (35,783), 20,107 (38,480) and 18,730 (35,361) peaks (2-kb bins) in MG, OPC, oRG and vRG, respectively. Bedtools merge was subsequently performed to create a universal H3K4me3 anchor set of 24,167 (47,412 2-kb bins) peaks (Extended Data Fig. 2b). HPRep^88^ was used to confirm the reproducibility of biological replicates. For each sample we took roughly 10 million usable reads to avoid differences caused by sequencing depth. We found a greater than 0.9 Pearson correlation between all biological replicates. In our final analysis, merged cell types were down sampled to roughly 60 million AND and XOR usable reads to maintain a consistent depth for downstream analysis. Significant interactions were identified using a Poisson regression-based approach.

### Defining cCREs

cCREs were subdivided into cCREs, cCREs^AR^ and cCREs^LMR^ with bedtools. Any base pair overlap between cCREs^AR^ and cCREs^LMR^ would be classified as cCREs. Comparatively, cCREs^AR^ and cCREs^LMR^ were exclusively accessible (AR) or LMR, respectively, and obtained with the ‘-v’ flag to indicate the absence of the corresponding feature. Chr. X and chr. Y were not included in the analysis. Intervene^89^ was used to generate an upset of cCREs and other upset plots.

### TF motif enrichment analysis

Motif enrichment analysis for both cell-type-specific cCREs and DARs were conducted with HOMER^34^ using the findMotifsGenome.pl command. Default parameters were used except ‘-size given’ was set. TFs of significant motifs were filtered for RPKM expression greater than 10 in the matching cell type.

### Heatmap of cell-type-specific cCREs

First, we determined XOR interactions specific to one cell type within our datasets containing a cCRE within the distal bin. Then the normalized contact score (observed/expected counts) for each bin-to-bin pair was determined by the observed count between bin pairs over the expected count generated by MAPs. Next, average ATAC-seq signal within each distal bin was obtained with bigWigAverageOverBed with counts per million (CPM) normalized signal. The heatmap shows the percentage of individual cell types divided by the sum of all cell types. If several peaks occur within the same distal bin, the average signal from each peak will be added together. ATAC-seq counts were further corrected for depth by quantile normalization. A similar technique was used for the transcriptome, but with RPKM of summed gene expression within the H3K4me3 anchor bin instead. The methylation percentage of distal bins were obtained by getting the average CpG methylation of ATAC peaks within the distal bin. Last, the unique interactions for each cell type were filtered to be overlapped with at least 50% of cCREs from the matching cell type.

### Mouse enhancer transgenic assay

Candidate elements for VISTA mouse transgenic assays were initially selected from ATAC-seq peaks identified in RG, IPC, eN and iN^3^. First, ATAC-seq reads were obtained within a merge peak set and subsequently quantile normalized. Second, peaks overlapping TSSs defined by cap analysis gene expression sequencing (CAGE-seq) were removed, and remaining regions were required to participate in a 3D chromatin interaction and overlap the top 15,000 accessible orthologous regions in mouse embryonic brain at E11.5 (refs. ^90,91^). Last, peaks were further filtered for strong ATAC-seq signal (greater than 0.8 CPM) and cell type specificity enrichment (greater than 0.5 CPM difference), yielding 61 candidate regions, which were further manually selected to 29 elements that largely overlapped with cCRE annotations (20 out of 29) or were chromatin interacting regions (20 out of 29, of which 16 are cCREs) identified in one of the vRG, oRG, OPC and MG datasets generated in this study (Supplementary Table 4). Transgenic mouse embryos were generated as described previously in ref. ^92^ with the exception that mouse embryos were collected at E12.5. Transgenic mouse assays were performed in *Mus musculus* FVB (friend leukaemia virus B) strain mice. Dark–light cycle was 12 h on, 12 h off (light on 6:00–18:00), temperature 20.6–23.9 °C (69–75 °F) and humidity 30–70%.

### VISTA elements enrichment

To determine which cCREs are associated with functional VISTA elements^24,25^, both neural VISTA elements (*n* = 1,233), defined as having neural tube, forebrain, midbrain, hindbrain, dorsal root ganglion, cranial nerve and trigeminal nerve, and negative controls (*n* = 1,868), defined as elements completely negative for signal, were overlapped with cCREs participating in 3D interactions and then compared to distance match control elements generated as previously described in ref. ^87^. Next, we compared the percentage of neural or negative elements overlapping cCREs and controls with the Fisher exact test.

To obtain potential targets of VISTA elements, positive VISTA neural elements determined to be both accessible and LMR were linked with target genes using bedtools. Target genes were further filtered for RPKM > 1 within the matching cell type.

### TOBIAS footprinting analysis and network analysis

Footprinting analysis was performed on merged BAM files from several sequencing runs per cell type. TF motifs were downloaded from HOCOMOCO v.11 database^93^. TOBIAS^30^ was run using standard parameters and workflow to identify TF footprints in each cell type individually. The list of ENCODE blacklist sites was taken with the TOBIAS ATACorrect tools when correcting for Tn5 insertion bias. Samples were processed in a pairwise manner to identify differential binding. Only TFs with RPKM > 10 were plotted on the volcano plot and included in the Pearson correlation with expression.

Motif binding predictions were classified as cell-type-specific if the motif was predicted to be bound in the corresponding cell type and if the absolute log_2_[fold change] was greater than one between two cell types. Target genes were subsequently identified as previously described. When visualizing each TF as a network with Cytoscape, only DEGs were visualized.

### Isolation and in vitro culture of oRG

The ventricular zone, inner subventricular zone and outer subventricular zone of a primary human cortical tissue sample at GW20 was dissected and dissociated using the Papain Dissociation System (Worthington Biochemical). Cells were infected with lentiviruses expressing GFP and shRNAs of either the scrambled control (shCTRL) or targeting *LHX2* (shLHX2_1 and shLHX2_2) (Supplementary Table 7). After 72 h, cells were collected and blocked by FcR Blocking Reagent (Miltenyi Biotech, 1:20) for 10 min, followed by antibody incubation for 30 min. Antibodies used for FACS include LIFR (leukaemia inhibitory factor receptor)-conjugated APC (R&D systems FAB249A) and PE-Cy7 anti-ITGA2 (BioLegend, 359314). GFP, ITGA2 and LIFR triple positive oRGs were collected. Then 50,000 cells per condition were seeded into 4 wells in a 24-well plate and cultured for 7 days in RG differentiation medium (DMEM/F12, 2 mM GlutaMAX, 2% B27 without vitamin A, 1% N2 and 1× penicillin–streptomycin (Pen/Strep)). Samples were subsequently processed using the 10× GEM-X Universal 3′ 4-plex on-chip multiplexing assay, targeting a range of 1,300–5,000 cells per replicate. Libraries of individual samples were pooled and sequenced on an Illumina NovaSeq-X plus sequencer.

### scRNA-seq analysis of oRG

The Cell Ranger (v.9.0.1) multi pipeline was implemented for cell barcode calling, read alignment and quality assessment using a custom human reference genome (GRCh38, GENCODE v.32/Ensembl98) with an enhanced GFP (eGFP) added according to the protocols described by 10X Genomics. Ambient RNA was removed from pooled gel bead-in-emulsions before downstream analysis with the CellBender^94^ (v.0.3.2) remove-background command. Next, we filtered for high-quality cells with the following criteria: (1) the number of detected genes (nFeature_RNA) was greater than 1,000; (2) less than 5% of all reads mapped to mitochondrial genes and (3) a doublet score, identified by scDblFinder^95^ (v.1.23.4) less than 0.3. A summary of the data quality is listed in Supplementary Table 7. The log-normalization with a size factor of 10,000, data scaling and cell-cycle regression of G2/M and S phase markers were performed in Seurat^96^ (v.5.4.0). Next, a nearest-neighbour graph was constructed with the first 30 principal components and clusters were identified with the Louvain algorithm. Clusters with low unique molecular identifier counts, most probably of low-quality cells, were removed and the clustering was repeated. Clusters were labelled as cell types on the basis of known marker genes (Extended Data Fig. 8c).

To identify which cell type showed the greatest difference between LHX2 knockdown and controls we used scDist^35^ (v.1.1.5), an R package that estimates sample difference in high-dimensional gene-expression space. For this analysis we used SCTransform^97^ (v.0.4.3) to normalize and scale the data as recommended. To identify changes in cell type distribution scCODA^98^ (v.0.1.9) was implemented with default settings.

### LDSC regression

We performed LDSC for each complex neuropsychiatric disorder by leveraging joint models incorporating either cCREs participating in H3K4me3-mediated interactions or distal 2,000 bp bins targeting anchor bins overlapping H3K4me3 signal across all cell types as well as a baseline model^99^ in Fig. 4a and Extended Data Fig. 8c, respectively. Whereas in Extended Data Fig. 8a,b we generated a joint model incorporating baseline, data from this study and datasets of RG, IPC, eN and iN ATAC-seq peaks either participating in 3D interactions or not interacting.

### Training the GKM model

Inspired by previously published studies^69,70^, we trained a GKM-SVM classifier to predict accessibility. For each cell type, the top 75,000 peaks were obtained for training on the basis of the MACS2 peak score. All peaks containing N bases were removed. To unify the training data, each peak was set to 1,000 bp by extending 500 bp from the summit. Negative training data were obtained with the genNullSeqs to obtain repeat and GC matched controls. To evaluate the model, we trained the model on all chromosomes except chr. 2, which was the test dataset. The ‘gkmtrain’ function of the LS-GKM package was used for training with default parameters including the wgkm kernel (*t* = 4). The performance was assessed on the test dataset using the ‘PRROC’ R package. Once the model was deemed successful, it was retrained using the complete dataset. To run deltaSVM, all 11-mers were generated with the ‘nrkmers.py’ python script and then evaluated by gkmpredict.

### In silico testing of variants

Both reference and alternative alleles of variants were evaluated in silico similar to previous methods^69^. Briefly Alzheimer’s^47^, rare non-coding^50^ and SCZ^53^ variants were filtered with bedtools as accessible in at least 1 cell type and then extended 100 bp up and downstream to a final length of 200 bp. Next, three techniques, deltaSVM^45^, ISM and GkmExplain^46^, were used to identify candidate active SNPs. For GkmExplain, only the difference between the central 50-bp region was used. Each method performed comparably, although some outliers occurred between techniques (PCC > 0.99) (Extended Data Figs. 8a and 9a). Significant SNPs were selected if the GkmExplain, ISM and deltaSVM scores resided outside the 95% confidence interval of null *t*-distributions. Furthermore, prominence and magnitude scores derived from seq-lets that potentially match TF motifs were used to help provide confidence of SNPs as has previously been done in ref. ^69^. Unlike previous approaches, we obtained prominence and magnitude scores for both positive and negative contributions because we reasoned that the model could also learn motifs of TFs that repressive chromatin accessibility. These scores can be found within Supplementary Tables 9 and 10.

### HAR enrichment testing

To test whether groups of cCREs are enriched for 3,168 annotated HARs^58^, we compared the overlap of cCREs of interest to the distribution of overlap with the same number of randomly sampled cCREs. For example, we observed that 4,515 unique vRG cCREs overlap with 30 HARs. Next, we randomly sampled 1,000 times. Each time, we sampled 4,515 cCREs from 121,317 cCREs obtained by merging vRG, oRG, OPC and MG cCREs and recorded the number of overlapping HARs. We thus obtained the empirical null distribution from 1,000 random samples. Last, we calculated the *z* score by comparing the observed 30 with the empirical null distribution. For Extended Data Fig. 9, 241,128 cCREs consisting of the union of vRG and oRG cCREs and IPC, iN and eN ATAC-seq peaks were sampled.

### Obtaining HAR variants

Variants of HARs were obtained similarly to the method previously described in ref. ^60^. In brief, we first obtained all alignments for HARs accessible in oRG using ‘mafsInRegion’. Next, ‘msa_view’ was used to convert the file into a multiple sequence alignment file in which only hg38 and pantro4 alignments were retained. Afterwards, the ‘snp-sites’ command was used to convert the MSA format into VCF format. Similar to above, each variant was extended 100 bp up and downstream to a final length of 200 bp and then evaluated by deltaSVM, ISM and GkmExplain.

### Obtaining predicted motif binding changes

To predict binding changes between human and chimpanzee we performed motifbreakR^61^ with the following data source, HOCOMOCOv11-core-A, HOCOMOCOv11-core-B and HOCOMOCOv11-core-C. Default parameters were used including a threshold of 1 × 10^−4^ and the ‘ic’ method. Only strong effects were retained and the motifs of TFs with more than 5 RPKM were retained for downstream analysis.

### CRISPRi knockdown of HARsv2_1313

The CROP-seq-opti-eGFP vector, which enables co-expression of puromycin resistance (PuroR), eGFP and dual gRNAs, was generated based on the CROP-seq-opti backbone (Addgene, 106280). eGFP was inserted downstream of the PuroR coding sequence and linked through a P2A self-cleaving peptide. Two pairs of gRNAs targeting HARsv2_1313 and one pair targeting the *ROCK2* promoter were designed using CHOPCHOP^100^ (Supplementary Table 10). Dual gRNAs were cloned into the CROP-seq-opti-eGFP vector according to a previously described protocol^101^.

For lentiviral production, gRNA plasmids (7.5 μg per T-75 flask) were cotransfected with pMD2.G (1.5 μg; Addgene, 12259) and psPAX2 (4.5 μg; Addgene, 12260) into 293T-LentiX cells (Takara Bio, 632180) using PolyJet transfection reagent (SignaGen, SL100688). Culture medium was replaced 18 h posttransfection and viral supernatants were collected daily for 3 consecutive days. Lentivirus was filtered using a 0.45-μm syringe filter and concentrated using Amicon Ultra-15 Centrifugal Filters (Millipore, UFC901024).

### iPS cell differentiation to NPCs

Human WTC11 iPS cells stably expressing dCas9-KRAB^47^ were maintained in mTeSR medium (STEMCELL Technologies, 100-0274) and tested regularly for mycoplasma. iPS cells were dissociated using Accutase and seeded onto Matrigel-coated plates at a density of 2.5 × 10^5^ cells per cm^2^ in basal medium consisting of DMEM/F12 (Thermo Fisher, 10565018), 1× N2, 1× B27 without vitamin A, 100 μM non-essential amino acids, 0.5 mg ml^−1^ BSA, 1× Pen/Strep and 100 μM 2-mercaptoethanol, supplemented with 20 ng ml^−1^ FGF2 and 10 μM Y-27632.

When cultures reached roughly 90% confluency (day 0), the medium was replaced with neural induction medium, composed of basal medium supplemented with 10 μM SB431542, 100 nM LDN193189 and 1 μM cyclopamine. From day 1 to day 11, fresh neural induction medium was changed daily. On day 12, NPCs were dissociated with Accutase and replated onto Matrigel-coated plates at 4.5 × 10^5^ cells per cm^2^ in neural stem-cell medium (NSCM) (Thermo Fisher, A10509-01) supplemented with 20 ng ml^−1^ FGF2, 20 ng ml^−1^ epidermal growth factor, 1× GlutaMAX, 30 μg ml^−1^ heparin, 0.2 mM ascorbic acid and 1× Pen/Strep. For the first passage, NSCM was also supplemented with 10 μM Y-27632.

Beginning on day 13, NSCM was replaced every other day until cultures became super confluent and ready for the next passage (roughly 5 days). NPCs were maintained in NSCM and used for downstream experiments between days 26 and 30.

### Quantitative PCR with reverse transcription

NPCs were transduced with lentivirus on day 21. Three days posttransduction, infected cells were enriched by puromycin selection for 3 days, followed by a 3-day recovery period. Total RNA was isolated using the AllPrep DNA/RNA Mini Kit (Qiagen, 80204). For complementary DNA (cDNA) synthesis, 200 ng of RNA was reverse transcribed using the iScript cDNA Synthesis Kit (Bio-Rad, 1708891). Quantitative PCR was performed using NEBNext Ultra II Q5 Master Mix (NEB, M0544) supplemented with 1× SYBR Green. The expression levels of *ROCK2* were normalized to GAPDH.

### Immunocytochemistry

NPCs were transduced with lentivirus on day 21, replated into 96-well plates on day 27 and fixed on day 29 with 4% paraformaldehyde. Fixed cells were blocked in PBS containing 0.1% Triton X-100 and 5% horse serum. Primary antibodies diluted in blocking solution were applied overnight at 4 °C, followed by incubation with secondary antibodies for 1 h at room temperature. The following antibodies were used: rat anti-Ki67-Alexa Fluor 647 (BioLegend, 151206; 1:200), rabbit anti-EMX2 (GeneTex, GTX17164; 1:400) and donkey anti-rabbit-Alexa Fluor 546 (Invitrogen, A10040; 1:500).

Stained NPCs were imaged using the Opera Phenix Plus high-content imaging system (Revvity) with a ×20 water-immersion objective. Image analysis was performed using the built-in Harmony software. Nuclei were identified based on basal Ki67 signal intensity (common threshold 0.30), and condensed Ki67 puncta were detected as high-intensity spots (relative spot intensity greater than 0.105). Cells were classified as Ki67-positive (Ki67^+^) if at least one condensed Ki67 punctum was detected within the nucleus. Mean GFP intensity was measured for each cell.

All downstream analyses were performed in R. GFP intensity values were log_10_-transformed for thresholding purposes. Cells with log_10_[GFP intensity] greater than 2.4 were defined as GFP-positive (GFP^+^) cells and included in subsequent analyses. This threshold was chosen on the basis of the distribution of GFP intensity in non-transduced control cells to distinguish background signal from true GFP expression. GFP^+^ cells from all treatment conditions were pooled and ranked according to raw GFP intensity. Cells were divided into quartiles (0–25%, 25–50%, 50–75% and 75–100%) based on the overall distribution. Quartile assignment was then mapped back to individual treatment groups. For each biological replicate in each condition, the percentage of Ki67^+^ cells was calculated within each GFP quartile. Ki67^+^ percentages in the top two quartiles, in which the CRISPRi knockdown effect became saturated, were compared between conditions. Bar plots represent mean ± s.e.m. A *P* value less than 0.05 was considered statistically significant.

To assess whether Ki67 positivity changed across increasing GFP quartiles within each treatment condition, simple linear regression was performed with quartile (coded numerically from 1 to 4) as the independent variable and Ki67^+^ percentage as the dependent variable. The slope and associated *P* value were extracted to evaluate the direction and statistical significance of the trend. Linear regression lines are shown with 95% confidence intervals. To compare trends between control and other treatment groups, linear regression models including an interaction term between quartile and treatment were used. The statistical significance of the interaction term was used to determine whether the slope of Ki67^+^ percentage across quartiles differed between treatment conditions.

### Luciferase experiments

The Dual-Luciferase Reporter Assay System (Promega, E1910) was used to test activity difference between chimpanzee and human variants within HARs. HAR sequences were amplified either with the human WTC11 iPS cell genomic DNA or chimpanzee C3649 (ref. ^102^) genomic DNA with NEBNext Ultra II Q5 (NEB, M0544L) using the same primers for each genome (Supplementary Table 11). Next, after Xho I and Nco I digestion of the pGL4.13 vector (Promega, E6681), we cloned HAR amplified DNA elements and a synthesized minimal promoter by means of Gibson assembly (NEB, E2621L), and validated this by Sanger sequencing.

The ventricular zone, inner subventricular zone and outer subventricular zone of primary human cortical tissue samples between GW17 and GW20 were dissected and dissociated using the Papain Dissociation System (Worthington Biochemical). The isolated cells were plated into 24-well plates precoated with poly-d-lysine at a density of 1.5 × 10^6^ per well. The culture medium was composed of 1× B27 without vitamin A, 1× N2, 0.1 mM 2-mercaptoethanol, 1× non-essential amino acids, 20 ng ml^−1^ FGF2, 20 ng ml^−1^ brain-derived neurotrophic factor, 20 ng ml^−1^ pleiotrophin, 20 ng ml^−1^ platelet-derived growth factor DD and 1× Pen/Strep in DMEM/F12 medium supplemented with GlutaMAX. After 24 h, the cells were cotransfected in triplicate with roughly 495 ng of the HAR vector and pRL-CMV-Renilla luciferase vector (Promega, E2261) at a 10:1 ratio by lipofectamine 3000 (L3000001). After 48 h, cell lysates were obtained with passive lysis. Briefly, each well was washed with 500 μl of PBS, then 100 μl of passive lysis buffer was added and the plates rotated at room temperature for 15 min. Finally, luciferase signals were obtained with the GloMax plate reader. For analysis, background signal was removed by subtracting the signal obtained from non-transfected controls and then the relative firefly luciferase activity was normalized to the average minimal promoter signal within each sample.

### Ethics statement

Human prenatal tissue samples were collected as de-identified specimens with previous informed consent, in strict accordance with applicable legal, institutional and ethical regulations. Acquisition, collection and use of these samples were approved by the Human Gamete, Embryo and Stem Cell Research Committee and Institutional Review Board at the University of California, San Francisco, USA. The Human Gamete, Embryo and Stem Cell Research Committee number is 10-05113. All experiments were performed in accordance with the approved protocol guidelines. All animal work was reviewed and approved by the Lawrence Berkeley National Laboratory Animal Welfare and Research Committee. Animals were inspected weekly by the Chair of the Animal Welfare and Research Committee and the head of the animal facility in consultation with the veterinary staff. The LBNL ACF is accredited by the American Association for the Accreditation of Laboratory Animal Care International.

### Reporting summary

Further information on research design is available in the Nature Portfolio Reporting Summary linked to this article.

## Online content

Any methods, additional references, Nature Portfolio reporting summaries, source data, extended data, supplementary information, acknowledgements, peer review information; details of author contributions and competing interests; and statements of data and code availability are available at https://doi.org/10.1038/s41586-026-10987-6.

## Supplementary information

Supplementary Fig. 1 Complete FACS gating strategy for vRG and oRG.

Reporting Summary

Supplementary Fig. 2 Complete FACS gating strategy for OPC and MG.

Supplementary Tables 1–11 Supplementary Table 1. Sample metadata and metrics. Supplementary Table 2. cCREs overview. Supplementary Table 3. Correlation between interactions and expression. Supplementary Table 4. Mouse transgenic assay validation. Supplementary Table 5. Epigenomic differences between vRG and oRG. Supplementary Table 6. LHX2 and ASCL1 binding. Supplementary Table 7. scRNA-seq metadata. Supplementary Table 8. GKM model training. Supplementary Table 9. In silico variant testing. Supplementary Table 10. Model prediction on HARs. Supplementary Table 11. Luciferase primers and results.

Peer Review File

## Data Availability

All raw datasets used in this study are under controlled access to protect donor privacy. These data can be requested from the Neuroscience Multi-Omic Archive (NeMO Archive) (NeMO ID nemo:dat-poyuhj7) for health/medical/biomedical purposes with local Institutional Review Board approval. Details for this process are available through the NeMO Archive at https://nemoarchive.org/resources/accessing-controlled-access-data#nda-approval-process. Processed data, including chromatin interactions, open chromatin peaks, methylation and gene-expression profiles for each cell type, as well as scRNA-seq datasets, are available through the NeMO Archive at https://assets.nemoarchive.org/dat-poyuhj7 and through the 4D Nucleome Data Portal at https://data.4dnucleome.org/. Single-cell data used with CIBERSORTx are available through UCSC cell browser at https://cells.ucsc.edu/cortex-dev/exprMatrix.tsv.gz. RG, IPC, eN and iN datasets are available through NeMO at https://assets.nemoarchive.org/dat-uioqy8b. Data can be visualized through the WashU Epigenome Gateway at https://epigenomegateway.wustl.edu/browser2022/?genome=hg38&sessionFile=https://shen-ijones.s3.us-west-1.amazonaws.com/NH_Final_Brower/eg-session-w0V39y_qR-fedd6750-1956-11f0-b3b4-fffa1c45cca7.json.

## Code availability

Data are analysed using published pipelines with parameters described in the Methods. Specific code used is available from GitHub at https://github.com/enrach21/3D-Epigenome-of-Glial-Cell-Types-in-Developing-Human-Cortex-Scripts.
