# Supplementary material for: 3D epigenome of glial cell types in developing human cortex
Source: Nature. 2026 Sep 2;658(8136):795–804. doi: 10.1038/s41586-026-10987-6 (PMC13668669; doi:10.1038/s41586-026-10987-6)
Supplement: Supplementary file 1 [file 41586_2026_10987_MOESM2_ESM.pdf]

nature portfolio

Corresponding author(s): Yin Shen, Arnold Kriegstein, R. David Hawkins

Last updated by author(s): Jun 24, 2026

Reporting Summary

Nature Portfolio wishes to improve the reproducibility of the work that we publish. This form provides structure for consistency and transparency in reporting. For further information on Nature Portfolio policies, see our Editorial Policies and the Editorial Policy Checklist.

Statistics

For all statistical analyses, confirm that the following items are present in the figure legend, table legend, main text, or Methods section.

n/a	Confirmed	
☐	☒	The exact sample size (*n*) for each experimental group/condition, given as a discrete number and unit of measurement
☐	☒	A statement on whether measurements were taken from distinct samples or whether the same sample was measured repeatedly
☐	☒	The statistical test(s) used AND whether they are one- or two-sided<br>*Only common tests should be described solely by name; describe more complex techniques in the Methods section.*
☒	☐	A description of all covariates tested
☐	☒	A description of any assumptions or corrections, such as tests of normality and adjustment for multiple comparisons
☐	☒	A full description of the statistical parameters including central tendency (e.g. means) or other basic estimates (e.g. regression coefficient) AND variation (e.g. standard deviation) or associated estimates of uncertainty (e.g. confidence intervals)
☐	☒	For null hypothesis testing, the test statistic (e.g. *F*, *t*, *r*) with confidence intervals, effect sizes, degrees of freedom and *P* value noted<br>*Give P values as exact values whenever suitable.*
☒	☐	For Bayesian analysis, information on the choice of priors and Markov chain Monte Carlo settings
☒	☐	For hierarchical and complex designs, identification of the appropriate level for tests and full reporting of outcomes
☐	☒	Estimates of effect sizes (e.g. Cohen's *d*, Pearson's *r*), indicating how they were calculated

Our web collection on statistics for biologists contains articles on many of the points above.

Software and code

Policy information about availability of computer code

Data collection	Sequencing data was obtained from the NovaSeq 54 instruments (Illumina).
Data analysis	Specific code used can be found on github(https://github.com/enrach21/3D-Epigenome-of-Glial-Cell-Types-in-Developing-Human-Cortex-Scripts). All software and code used in the text are now reported in the text along with their version numbers. We used the following software: STAR (v2.7.10a),<br>RSEM (vl.2.28),<br>fastp (v0.22.0),<br>edgeR (v3.32.1),<br>limma(v3.46.0),<br>deepTools(v3.5.1),<br>DiffBind(v3.4.11),<br>ENCODE pipeline (https://github.com/kundajelab/atac_dnase_pipelines),<br>TrimGalore v0.6.6,<br>Bismark v0.16.2,<br>Bowite2 v2.4.1,<br>MAPS (1.0),<br>DESeq2 (vl.30.1),<br>LDSC (vl.0.1),<br>Gprofiler (v0.2.1), R (v4.0.5),<br>Python (v3.8.10),<br>HPRep (Rosen et al. 2021), bedtools (v2.30.0),

Cytoscape (v3.10.3),
Intervene (v 0.5.5),
GKM-explain (Shrikumas et al. 2019) motifbreakR (v2.12.3)
HOMER (v4.11),
TOBIAS (0.8.0),
scDist (1.1.5),

For manuscripts utilizing custom algorithms or software that are central to the research but not yet described in published literature, software must be made available to editors and reviewers. We strongly encourage code deposition in a community repository (e.g. GitHub). See the Nature Portfolio guidelines for submitting code & software for further information.

Data

Policy information about availability of data

All manuscripts must include a data availability statement. This statement should provide the following information, where applicable:

- Accession codes, unique identifiers, or web links for publicly available datasets
- A description of any restrictions on data availability
- For clinical datasets or third party data, please ensure that the statement adheres to our policy

All raw datasets used in this study are under controlled access to protect donor privacy. These data can be requested from the Neuroscience Multi-Omic Archive (NeMO Archive) (NeMO ID: nemo:dat-poyuhj7) for health/medical/biomedical purposes with local IRB approval. Details for this process are available at https://nemoarchive.org/resources/accessing-controlled-access-data#nda-approval-process.

Processed data, including chromatin interactions, open chromatin peaks, methylation, and gene expression profiles for each cell type, as well as scRNA-seq datasets, can be downloaded from the NeMO Archive at https://assets.nemoarchive.org/dat-poyuhj7 and from the 4D Nucleome Data Portal (https://data.4dnucleome.org/).

Single-cell data used with CIBERSORTx can be obtained at https://cells.ucsc.edu/cortex-dev/exprMatrix.tsv.gz. RG, IPC, eN and iN datasets can be accessed at https://assets.nemoarchive.org/dat-uioqy8b.

Data can be visualized at the following link: https://epigenomegateway.wustl.edu/browser2022/?genome=hg38&sessionFile=https://shen-ijones.s3.us-west-1.amazonaws.com/NH_Final_Brower/eg-session-w0V39y_qR-fedd6750-1956-11f0-b3b4-fffa1c45cca7.json

Research involving human participants, their data, or biological material

Policy information about studies with human participants or human data. See also policy information about sex, gender (identity/presentation), and sexual orientation and race, ethnicity and racism.

Reporting on sex and gender	Sex and gender were not considered in this study. Analysis was performed on autosomes.
Reporting on race, ethnicity, or other socially relevant groupings	N/A.
Population characteristics	No population characteristics were collected.
Recruitment	N/A.
Ethics oversight	*Identify the organization(s) that approved the study protocol.*

Note that full information on the approval of the study protocol must also be provided in the manuscript.

Field-specific reporting

Please select the one below that is the best fit for your research. If you are not sure, read the appropriate sections before making your selection.

☒ Life sciences ☐ Behavioural & social sciences ☐ Ecological, evolutionary & environmental sciences

For a reference copy of the document with all sections, see nature.com/documents/nr-reporting-summary-flat.pdf

Life sciences study design

All studies must disclose on these points even when the disclosure is negative.

Sample size	No statistical methods were used to predetermine sample size. We chose sample size based on experimental and budget constraints. Although we didn't choose sample sizes based on power calculations, we applied multiple testing to ensure all significant findings are statistically valid including clustering and comparison with other datasets.
Data exclusions	No data were excluded from the analyses.

Replication	All attempts at replication were successful and are described in the text. A minimum of two replicates were generated for all omic assays, with the majority having three or more.
Randomization	There was no randomization for all samples. We adjusted covariates whenever appropriate. For differential analysis between vRG and oRG they date the samples the processed was used.
Blinding	Blinding is not relevant to our study because

Reporting for specific materials, systems and methods

We require information from authors about some types of materials, experimental systems and methods used in many studies. Here, indicate whether each material, system or method listed is relevant to your study. If you are not sure if a list item applies to your research, read the appropriate section before selecting a response.

Materials & experimental systems

n/a	Involved in the study	
☐	☒	Antibodies
☐	☒	Eukaryotic cell lines
☒	☐	Palaeontology and archaeology
☐	☒	Animals and other organisms
☒	☐	Clinical data
☒	☐	Dual use research of concern
☒	☐	Plants

Methods

n/a	Involved in the study	
☒	☐	ChIP-seq
☐	☒	Flow cytometry
☒	☐	MRI-based neuroimaging

Antibodies

Antibodies used	The antibodies used for FACS included PerCP-Cy5.5 anti-SOX2 (BD Biosciences, 561506), PE-Cy7 anti-EOMES (lnviteogen, 25-4877-42), unconjugated anti-HOPX (Proteintech, 11419-1-AP), Alexa Fluor 647 anti-OLIG2 (Abeam, ab225100), PE anti-PU.l (Cell Signaling Technology, 81886), Human LIFR alpha APC-conjugated Antibody (R&D systems FAB249A), and PE/Cyanine7 anti-human CD49b Antibody (BioLegend, 359314). The HOPX antibody was used at 1:250 dilution, while all other antibodies were used at 1:20 dilution.<br>Antibody used for PLAC-seq was Anti-trimethyl-Histone H3 (Lys4) Antibody, clone MC315, rabbit monoclonal (Millipore 04-745)
Validation	PerCP-Cy5.5 anti-SOX2 (BD Biosciences, 561506): Manufacture routinely tested for intracellular staining (flow cytometry)<br>PE-Cy7 anti-EOMES (lnviteogen, 25-4877-42): From manufacture, "Applications Tested: This WD1928 antibody has been pre-titrated and tested by intracellular staining and flow cytometric analysis of normal human peripheral blood cells using the Foxp3/ Transcription Factor Staining Buffer Set (Product# 00-5523-00) and protocol. Please refer to BestProtocols®: Protocol B: One step protocol for (nuclear) intracellular proteins located under the Resources Tab online.This can be used at 5 µL (0.06 µg) per test. A test is defined as the amount (µg) of antibody that will stain a cell sample in a final volume of 100 µL. Cell number should be determined empirically but can range from 10A5 to 10A8 cells/test."<br>unconjugated anti-HOPX (Proteintech, 11419-1-AP): Manufacture positively detected western blot in mouse lung tissue, mouse small intestine tissue, and rat lung tissue as well as immunohistochemistry in human placenta tissue and mouse lung tissue.<br>Alexa Fluor 647 anti-OLIG2 (Abeam, ab225100):): Manufacture tested immunohistochemistry in mouse and rat and predicted to work in human.<br>PE anti-PU.l (Cell Signaling Technology, 81886): From manufacture. "This Cell Signaling Technology antibody is conjugated to phycoerythrin (PE) and tested in-house for direct flow cytometry analysis in human cells. This antibody is expected to exhibit the same species cross-reactivity as the unconjugated PU.l (9G7) Rabbit mAb #2258."<br>Human LIFR alpha APC-conjugated Antibody (R&D systems FAB249A): Manufacture validated for Flow Cytometry in humans.<br>PE/Cyanine7 anti-human CD49b Antibody (BioLegend, 359314): From manufacture. "Each lot of this antibody is quality control tested by immunofluorescent staining with flow cytometric analysis."<br>Anti-trimethyl-Histone H3 (Lys4) Antibody, clone MC315, rabbit monoclonal (Millipore 04-745): From manufacture. "Anti-trimethyl-Histone H3 (Lys4) Antibody, clone MC315 is a rabbit monoclonal antibody for detection of trimethyl-Histone H3 (Lys4) also known as H3K4me3, Histone H3 (tri methyl K4) & has been validated in WB, ChIP, DB, Mplex, ChIP-seq."

Eukaryotic cell lines

Policy information about cell lines and Sex and Gender in Research

Cell line source(s)	Human WTC11 iPSCs stably expressing dCas9-Krab (Yang et al., 2023 Nature Genetics PMID 37735198). Lenti-X 293T Cell Line (Takara, 632180) is a subclone of the transformed human embryonic kidney cell line, HEK293, which is highly transfectable and supports high levels of viral portien expression.
Authentication	No additional authentication was used as these cell lines and differentiation were previously characterized.
Mycoplasma contamination	All cells used in the present study were verified as mycoplasma contamination free.

Commonly misidentified lines (See ICLAC register)	None of the cell lines used are commonly misidentified lines.

Animals and other research organisms

Policy information about studies involving animals; ARRIVE guidelines recommended for reporting animal research, and Sex and Gender in Research

Laboratory animals	Transgenic mouse assays were performed in Mus musculus FVB strain mice at embryonic day Ell.5. Dark light cycle: 12 hrs on, 12 hrs off (light on 6am-6pm), Temperature: 69-75F, Humidity: 30-70%.
Wild animals	N/A
Reporting on sex	Animals of both sexes were used for analysis. We observed no variability consistent with potential sex-specific expression of the enhancers in questions.
Field-collected samples	Not collected.
Ethics oversight	All animal work was reviewed and approved by the Lawrence Berkeley National Laboratory Animal Welfare and Research Committee. Animals were inspected weekly by the Chair of the Animal Welfare and Research Committee and the head of the animal facility in consultation with the veterinary staff. The LBNL ACF is accredited by the American Association for the Accreditation of Laboratory Animal Care International (AAALAC).

Note that full information on the approval of the study protocol must also be provided in the manuscript.

Plants

Seed stocks	N/A
Novel plant genotypes	N/A
Authentication	N/A

Flow Cytometry

Plots

Confirm that:

- [x] The axis labels state the marker and fluorochrome used (e.g. CD4-FITC).
- [x] The axis scales are clearly visible. Include numbers along axes only for bottom left plot of group (a 'group' is an analysis of identical markers).
- [x] All plots are contour plots with outliers or pseudocolor plots.
- [x] A numerical value for number of cells or percentage (with statistics) is provided.

Methodology

Sample preparation	Cells were isolated from the developing human cortex between gestational weeks (GW) 15 and 24 similarly as previously described3. Dissociated cells were washed twice in PBS and fixed in 2% PFA for 10 min at room temperature. Fixation was quenched by adding glycine to a final concentration of 200 mM, followed by incubation for 5 min at room temperature. From this point onward, all procedures were performed either on ice or at 4 ºC. Fixed cells were pelleted by centrifugation at 1,000×g, washed once with PBS, filtered through a 70-µm nylon mesh, and washed once again with PBS. Finally, the cells were pelleted and stored at -80 ºC. All procedures were performed either on ice or at 4 ºC. About 1.5×10^8 fixed cells were thawed and permeabilized by incubating in 1 mL PBS containing 0.1% Triton X-100 for 15 min. BSA was added to a final concentration of 1% and cells were pelleted by centrifugation at 1,000×g for 8 min. Cells were washed once in staining buffers (PBS with 1% BSA) and resuspended in a 100 mL staining buffer. Cells were blocked by FcR Blocking Reagent (Miltenyi Biotech, 1:20) for 10 min, followed by antibody incubation for 30 min.
Instrument	Cells were sorted using BD FACSAria II sorters into collection buffer (PBS with 5% BSA).
Software	Data collection was performed using FACsDiva. Representative figures are generated by FlowJo.

Cell population abundance

Ventricular radial glia and outer radial glia represent ~3.5% of singlets from GW16-GW18. Oligodendrocyte precursor cell and microglia represent ~0.5% and ~3% of singlets from GW22-GW24 respectively.

Gating strategy

The gating strategy is described in Fig. 1a,b. Gates for positive and negative populations were defined based on unstained controls and the separation between positive and negative subpopulations within the stained sample. To distinguish vRG and oRG populations, HOPX high and HOPX low gates were established relative to the HOPX staining intensity of IPCs. Cells with HOPX signal higher than the IPC population were classified as oRGs, while those with comparable levels were classified as vRGs.

☒ Tick this box to confirm that a figure exemplifying the gating strategy is provided in the Supplementary Information.
