# Supplementary material for: 3D epigenome of glial cell types in developing human cortex
Source: Nature. 2026 Sep 2;658(8136):795–804. doi: 10.1038/s41586-026-10987-6 (PMC13668669; doi:10.1038/s41586-026-10987-6)
Supplement: Supplementary file 2 [file 41586_2026_10987_MOESM5_ESM.pdf]

nature portfolio

Peer Review File

3D Epigenome of Glial Cell Types in Developing Human Cortex

Corresponding Author: Dr Yin Shen

Any redactions in this file are there to maintain patient confidentiality, the confidentiality of unpublished data, or to remove third-party material.

This file contains all reviewer reports in order by version, followed by all author rebuttals in order by version.

Version 0:

Reviewer comments:

Referee #1

(Remarks to the Author)
The manuscript by Jones et al., has generated a comprehensive dataset for the investigation of genomic and epignomic regulation in glial cell types in the mid-gestational developing human brain. In particular, the study applied a cell sorting scheme that can effectively separate the related vRG and oRG. Using the enriched cell populations, the authors generated three types of epigenomic profiles including ATAC-seq, WGBS, and PLAC-seq (or HiChIP). The data resource is complementary to published single cell datasets for two main reasons: 1) the published single cell HiC works have limited ability in distinguishing oRG from vRG. 2) the PLAC-seq data allows chromatin loops to be identified at a 2kb resolution, providing a greater spatial resolution than the single cell HiC approaches. The study took advantage of the VISTA enhancer resource in developing mouse brain, in addition to some new transgenic mice experiments to demonstrate that the identified cCREs are functional regulatory elements. The main weakness of the current manuscript is that the rich dataset was not fully explored through computational analyses. As discussed below, the manuscript did not fully explore PLAC-seq contacts that do not overlap with a cCRE, which accounts for 75% of the contacts. The predictive modeling works that determine the effect of variants on cCREs can be expanded to rare variants associated with neurodevelopmental disorders. The authors also have an opportunity to integrate the data produced in this study with the dataset described in Song 2020 Nature (from the same groups of senior authors), to provide a more comprehensive view of the regulatory programs in the mid-gestational human brain.

Major comments:
1.The manuscript focused on PLAC-seq loops overlapping with cCREs. But these only account for ~25% of loops. The functional significance of chromatin loops not overlapping with a distal cCRE is not been sufficiently explored. One such example is PTPRG shown in Fig. 2f. Although the discussion focused on VISTA enhancers hs434 and hs435, these two elements are relatively weakly connected to PTPRG. What is the potential regulatory significance of other regions connecting to the gene, if they are not cCREs?
2.In line 134, the manuscript stated that more than two-thirds of interaction clusters contain a cCRE. But 15 kb is a large space, and no statistics were done to support the observation was more significant than the random distribution of cCRE.
3.Why do the regions shown in Fig. 2c show such a strong specificity for chromatin contact frequency? Were the regions selected for high chromatin contact only in one cell type? Alternatively, does the result suggest chromatin contact being the most sensitive data modality for distinguishing the cell types?
4.Is it surprising to find ASCL1 as a major TF driving the distinction between dorsal vRG and oRG? To my knowledge, ASCL1 has critical roles in inhibitory neuron differentiation.
5.The manuscript compared the enrichment of disease heritability at interacting cCREs and all interacting 2kb bins. Did the authors also compare to non-interacting cCREs?
6.The footprinting-based approach for analyzing TF binding may not be distinguish the binding signature of TCF4 and TCF12 , which share a CACGTG binding motif. Such a caveat should be discussed in the manuscript.
7.The predictive modeling of the effect of variants on cCREs may be expanded to rare variants such as those identified by the Simons Simplex Collection for autism, or rare variants associated with other types of neurodevelopmental disorders.
8.Although this study focused on the distinction between glial cell types. It seems natural to integrate the Song 2020 dataset and the newly generated dataset to better understand the regulatory dynamics from vRG to oRG to newborn neurons.

Referee #2

(Remarks to the Author)
This paper by Jones et al is one of the very first to study in a 'multiomic' approach multiple glial cell types in the human fetal brain , with the critical element of FACS-sorting/separating outer radial glia (oRG) from ventricular radial glia and comparing these two cell types. the oRG are thought to play a critical role in primate brain evolution.

Major Comment:
Figure 5 is the most exciting result of the paper, showing that cis-regulatory elements specific to the oRG are enriched for human accelerated (HAR) DNA sequences. Could the authors add a cell function-based experiment, some sort of cell biology or related assay to show for at least one of their oRG cis-regulatory elements at an HAR, how it modifies oRG function and/or how it could contribute to some of the unique features of the human brain?

Minor:
The cell-specific differences in the figure 2f chromosomal contact map are very hard to decipher. perhaps adding differential contact map/track may help.

Technical comment:
figure 1a HOPX marker-based FACS does not produce two separate clouds for vRG and oRG; while I believe the authors that they are successfully enriching/separating ensembles of vRG and oRG, I feel a standard single cell(nuclei) 10x genomics or similar single cell RNA-seq assay as quality control check for this key experiment would greatly benefit the paper.

Referee #3

(Remarks to the Author)
In their manuscript entitled “3D Epigenome of Glial Cell Types in Developing Human Cortex”, Jones, Wang et al combine transcriptomic and epigenomic analyses of four major glial populations in the mid-gestational human neocortex (ventricular radial glia (vRG), outer radial glia (oRG), oligodendrocyte precursor cells (OPC), and microglia (MG)). To do this, they generated high-resolution RNAseq, ATACseq, DNA methylation, and PLAC-seq datasets of sorted vRGs (EOMES-, HOPXlow), oRGs (EOMES-, HOPXhi), microglia (PU.1+), and oligodendrocytes (OLIG2+) purified from second-trimester cortical samples. By prioritizing candidate regulatory sequences (CRSs) that were both low methylation and chromatin accessible, they identify 60,000-70,000 cCREs per cell type. By H3K4me3-mediated PLAC-seq they identify 135,000-145,000 gene regulatory chromatin interactions, 21.5-26.4% of which contained cCREs. Of 15 oRG cCREs examined, 11 were confirmed by mouse transgenic assays. Using machine learning, they resolved known AD SNP rs636317, and further prioritize the SCZ variant rs4449074, again validated by mouse transgenic assay. Yet, the study lacks large-scale functional validation, cross-disorder GWAS analyses to uncover glia cell-type-specific disorder enrichments, or a systematic analysis of inter-donor variation in cell-type-specific CREs. Altogether, this manuscript presents a significant and ambitious attempt to characterize and predict glial cell type specific epigenetic and transcriptomic effects in the developing human brain but falls short of functionally dissecting glia cell type specific disease mechanisms. With revisions, this manuscript may be suitable for publication in Nature.

Major Concerns:

1. Reproducibility: The authors generated a high-resolution atlas of the 3D epigenome and transcriptome in the developing human cortex. Ideally the reader would be able to note key facts about the number of donors per dataset and data quality ranges per dataset in the main text. Although SI Table 1 transparently reveals the scope of the dataset generated, the extent of inter-donor variation is not discussed.
a. The main text does not discuss how many donors were included in the study. From sample metadata in SI Table 1, in total 15 donors were included, but only four for vRG/oRG and three for OPC/MG RNAseq/ATACseq; there are no donors with matched vRG/oRG/OPC/MG data. Likewise, methylation data derived from two donors per cell type and PLAC-seq from three. Is it possible to include at least one set of donor-matched analyses?
b. Please provide basic QC information per dataset (e.g., the range of postmortem interval, RNA RIN, RNAseq percent mapping (89.58-92.75), ATACseq fraction of reads in peaks (0.32-0.53), etc) in methods.
c. The authors demonstrate that their vRGs and oRGs, or OPCs and MGs, are distinct from each other (Fig. 1d). It would be helpful if they included key markers to confirm that the cells are not neurons (e.g., NeuN, MAP2) or astrocytes (e.g., S100b). Better yet, are the cell type signatures similar to those in other published datasets.
d. It would be helpful to resolve the variability between RNAseq, ATACseq, DNA methylation, and PLAC-seq between donors, batches, and any other plausible sources of variation. It’s unclear the extent to which peaks were reproducible across donors, or the extent to which one sample may have biased calls. What is the inter-donor correlation?
e. Please summarize the number of peaks, chromatin interactions, CREs called by cell type per donor. Are there outlier CREs/DARs/chromatin loops not detectable across replicated that should be removed?

2. Functional validation: The tens of thousands of CREs and chromatin interactions identified across human neocortex vRGs, oRGs, OPCs, and MGs, as well as their links to brain disease, are minimally validated.
a. The authors identify 60,000-70,000 CREs per cell type, but only experimentally validate 15 in the developing mouse. Moreover, the CREs examined in vivo are not explored in a cell type specific manner. Are the tested CREs active in the predicted cell types? Are they specific to the predicted cell types?
b. Given the scope of CREs identified, it would be substantially more informative to test them using massively parallel reporter assays. Ideally, validation would be done in a human specific and cell type specific context, either in primary human and/or human induced pluripotent stem cell derived populations, but doing so in transformed glial cell lines would likely be informative as well.
c. Of 15 oRG cCREs examined, 11 were confirmed by mouse transgenic assays. Yet, the rationale for selecting these 15 oRG CREs is unclear. Moreover, the in vivo transgenic assay is not described in the methods and there is no detailed description in the figure legend of the results presented Figure 2F/4C.

3. Disease relevance: The authors apply a machine learning model to explore whether GWAS SNPs linked to Alzheimer's disease and schizophrenia credibly impact chromatin accessibility.
a. Of the 565 of 6,350 credible AD variants that were accessible in at least one cell type, among which 68 were predicted to perturb accessibility, the authors highlight only one example (rs636317). Of the 929 of 11,360 SCZ variants that were accessible, 112 were predicted to perturb accessible. Rather than highlight one SNP, be it rs636317 or rs4449074, it would be substantially more informative to compare GWAS from many brain diseases/disorders/traits and present which GWAS are most enriched for which glial cell types.
b. The authors highlight that both vRG and oRG cCREs and DARs enriched for annotated HARs. Was this likewise true for OPCs and MGs?
c. It's unclear why the analysis of HARs, and TF binding withing HARs, was restricted to oRGs. Could this analysis be integrated into the larger cross-disorder GWAS suggested above? Identifying disease loci within TF binding sites and/or HARs?
d. Again, functional validation of these highlighted CREs by MPRA or CRISPRprime and/or functional validation of the cell-type-specific role of these TFs would dramatically inform the relevance of the analyses presented.

Referee #4

(Remarks to the Author)
The manuscript "3D Epigenome of Glial Cell Types in Developing Human Cortex" by Jones et al. provides a comprehensive examination of transcriptional regulation during neurogenesis, using multi-omics approaches to uncover key differences in radial glial cells (vRG, oRG), OPCs, and microglia during early human development. The authors employ bulk RNA sequencing with ATAC-seq and PLAC-seq to identify cell-type-specific cis-regulatory elements and validate these findings in mouse models. This innovative multi-omics strategy enables the identification of active genomic regions that control gene expression in a highly specific manner, according to cell type. The study highlights the critical role of transcription factor binding sites in regulating gene expression and driving cell fate specialization in oRGs and vRGs. Importantly, the research reveals that oRGs exhibit an exceptionally high density of cis-regulatory elements within HAR regions, offering novel insights into the regulatory mechanisms governing human cortex development. The authors apply machine learning algorithms to predict the effects of GWAS risk variants on gene regulation, focusing specifically on the rs4449074 variant associated with schizophrenia. This work has significant implications for understanding the intricate relationships between genetic variation and gene regulation in the developing brain. While this manuscript provides valuable insights into glial cell development, it also provides a detailed analysis of radial glial cells transitioning to oRGs and vRGs.

Regrettably the manuscript is an incremental step from previous work by the same lab (Song et al., 2020), where it extends the analysis to subtypes of radial glial cells but somewhat superficially includes OPCs and microglia. The manuscript would benefit from more comprehensive comparisons with the previous study, particularly those examining intergenic risk factors associated with GWAS variants across all cell types in human tissue at this stage. To further validate their findings, the authors could investigate what changes occur in oRG biology as a result of the rs4449074 variant compared to non-carriers. Moreover, functional validation of the predicted regulatory elements is essential to establish their biological significance and the causal role they play in controlling gene expression during neurogenesis. While the bioinformatic analysis provides a broad spectrum of insights, the absence of experimental validation reduces the impact of the study in its current form. Nevertheless, this research has the potential to address key questions and make significant contributions to our understanding of gene regulation during neurogenesis, ultimately informing human health and disease

Major points

1. The manuscript lacks experimental validation of the predicted regulatory elements, which is essential to establish their biological significance and the causal role they play in controlling gene expression during neurogenesis.

The manuscript relies heavily on bioinformatic analysis to identify potential regulatory elements, but lacks experimental validation of the predicted regulatory elements. This is essential to establish their biological significance and the causal role they play in controlling gene expression during neurogenesis, and such as driving cell fate specialization in oRGs and vRGs,. To address this, the authors could for example conduct functional assays (e.g. CRISPR-Cas9 knockout or overexpression experiments) in organoids, or fetal cell cultures to demonstrate that these predicted regulatory elements indeed control gene expression during neurogenesis.

For examples are CRE switches needed for the transition or the consequence of the transition? LIFR signalling has been shown to regulate oRG numbers in organoids and is indicated here in the network of interactions for LHX2. The role of LHX2 in human RG function is not clear, does its regulation and expression have a developmental effect. Would any of the identified CRE candidates and TFs such as LHX2 alter vRG to oRG transition? There is no evidence for the TF binding to the cCREs in RGs or data showing alteration or removal of the changes caused by putative binding site alterations.

Functional studies would provide necessary evidence for the biological relevance of these findings and strengthen the conclusions drawn from the bioinformatic analysis.

2.The manuscript builds upon previous work from the same lab (Song et al., 2020) but could benefit from more detailed comparisons to highlight its novel contributions and extend our understanding of early human neurodevelopment. In particularly for examining intergenic risk factors associated with GWAS variants and all cell types in human tissue at this stage. This would provide a more understanding of how these regulatory elements interact with genetic variation and gene regulation during neurogenesis.

3. The manuscript highlights the importance of transcription factor binding sites in CREs in regulating gene expression and driving cell fate specialization in oRGs and vRGs, the authors could delve deeper into how psychiatric GWAS hits interact with these regulatory elements, and their implications in various neuropsychiatric disorders. Importantly the authors should investigate whether these psychiatric GWAS hits affect the predicted regulatory elements, particularly for distal sequences, which would provide valuable insights into the relationship between genetic variation and gene regulation in the developing brain.

In Figure 4, Ext fig 8 the authors correlate schizophrenia and GWAS variant rs4449074 with RGs however their data indicates higher MG and OPC SNP accessibility changes, but these changes not investigated. It is unclear if the SATB2 variant is sufficient to cause changes in upper layer specification or neuron numbers during development? This could be also tested using in vitro organoid or ex vivo slice models.

Referee #5

(Remarks to the Author)
I co-reviewed this manuscript with one of the reviewers who provided the listed reports.

Version 1:

Reviewer comments:

Referee #1

(Remarks to the Author)
The authors have done a comprehensive work in addressing the comments. I have one additional comment related to one of the responses.

1. With additional analyses, the authors showed that 75% PLAC-seq interactions do not involve significant signatures of regulatory elements, defined by VISTA-validated enhancers, LMRs, or accessibilities. Therefore, these regions are considered less interesting in the context of this study. This is a reasonable, although unexciting, conclusion. Are these regions more related to the structural elements of chromatin folding, such as those bound by CTCF or cohesin? The authors should discuss these results in the manuscript, including the negative finding that 75% of interactions do not engage regulatory activity, for completeness.

Referee #2

(Remarks to the Author)
The authors addressed all of my comments properly and likewise, those raised by the other Reviewers. The paper is now much improved.

Referee #3

(Remarks to the Author)
In their revised submission “3D Epigenome of Glial Cell Types in Developing Human Cortex,” Jones, Wang et al provide a detailed response to feedback from all reviewers; in my opinion, they very well addressed all concerns! I am very satisfied with the improved clarity by which data is now presented by replicate in SI Fig. 1. Likewise, I commend the authors with their efforts towards functional validation, incorporating a published MPRA dataset to validate CREs and new shRNA- and CRISPR-mediated experiments to test a top transcription factor and HAR identified in their analyses. Finally, the authors did a thoughtful job integrating their analyses with their previous study of 3D genome structure in neurons as well as with cross-disorder GWAS. This manuscript is a pleasure to read and will be a valued resource to the field. I am happy to recommend it

for publication at Nature!

- Kristen Brennand

Referee #4

(Remarks to the Author)
The manuscript has improved substantially since the previous submission, and the authors have clearly made a serious effort to address the reviewers' concerns. The addition of functional data and further bioinformatic analyses has significantly strengthened the study.
A small number of points remain where clarification would improve interpretability.

The main outstanding issue may reflect a misunderstanding on the reviewer's part; however, the current presentation makes it difficult to reconcile some conclusions with the data, particularly regarding the functional experiments on HARsv2_1313.
The main outstanding issue may reflect a misunderstanding on the reviewer's part; however, the current presentation makes it difficult to reconcile some conclusions with the data, particularly in relation to the functional experiments on HARsv2_1313.
Specifically,
• ROCK2 knockdown reduces ROCK2 mRNA and increases Ki67^{+} cells;
• HARg1 perturbation shows little or no reduction in ROCK2 mRNA, yet also increases Ki67;
• HARg2 perturbation leads to a stronger reduction in ROCK2 mRNA, but does not increase Ki67.
This pattern does not support a consistent relationship between ROCK2 levels and proliferation. Therefore, statements such as "...these results suggest that reduced HARsv2_1313 activity in humans decreases ROCK2 expression and increases proliferation…" and "...CRISPR interference… established a causal link between this predicted regulatory element and a relevant cellular phenotype...", appear overstating the data.
It would therefore be helpful if the authors could clarify whether the proliferation effects are mediated via ROCK2, or whether HARsv2_1313 may have additional targets mediating these effects. Alternatively, a mechanistic explanation for the apparent decoupling between ROCK2 mRNA levels and proliferation in the HARg1 versus HARg2 perturbations would strengthen the interpretation.
Additional supporting data would also improve confidence in these conclusions, including evidence for ROCK2 protein levels or kinase activity, alternative measures of proliferation beyond Ki67, and representative images for the quantifications.

Minor points
1. Extended Figure 3f: please clearly indicate the cell type corresponding to each bar.
2. Minor typographical errors should be corrected throughout, such as "Isolation and in vivo culture of oRG" — "in vivo" should likely read "in vitro"; Figure 2b-"Count stength" should read "Count strength" etc..

Version 2:

Reviewer comments:

Referee #4

(Remarks to the Author)
The authors have now addressed all of our concerns. We appreciate their explanation of the relationship between GFP intensity (as a proxy for perturbation strength) and Ki67 positivity, which clarifies the interpretation of the ROCK2 and proliferation data. As this relationship is important to the conclusions, it may be helpful to illustrate it more explicitly in Figure 5 or as an Extended Data panel, in addition to the description in the text.

Referee #5

(Remarks to the Author)
I co-reviewed this manuscript with one of the reviewers who provided the listed reports.

Open Access This Peer Review File is licensed under a Creative Commons Attribution 4.0 International License, which permits use, sharing, adaptation, distribution and reproduction in any medium or format, as long as you give appropriate credit to the original author(s) and the source, provide a link to the Creative Commons license, and indicate if changes were made.
In cases where reviewers are anonymous, credit should be given to 'Anonymous Referee' and the source.
The images or other third party material in this Peer Review File are included in the article's Creative Commons license, unless indicated otherwise in a credit line to the material. If material is not included in the article's Creative Commons license and your intended use is not permitted by statutory regulation or exceeds the permitted use, you will need to obtain permission directly from the copyright holder.
To view a copy of this license, visit https://creativecommons.org/licenses/by/4.0/

Executive Summary:

We sincerely thank all reviewers for their thoughtful and constructive feedback. In response to the reviewers' comments, we performed additional experiments and analyses to strengthen the manuscript, including deeper integration with published datasets, expanded analyses of disease-associated variants, and additional functional validation of predicted regulatory elements. These new analyses addressed many of the reviewers' major shared concerns:

1. **Reviewers 1 & 4 have asked for better integrative analyses with published datasets.**

We extended our linkage disequilibrium score regression (LDSC) analyses to include accessible chromatin peaks from radial glia (RG), intermediate progenitor cells (IPC), excitatory neurons (eN), and inhibitory neurons (iN) from Song et al. These analyses show that chromatin peaks involved in 3D interactions are enriched for neuropsychiatric disease heritability across multiple developmental cell types, while non-interacting peaks show enrichment primarily in glia. This demonstrates that 3D chromatin architecture helps refine disease-relevant regulatory elements in a cell type–specific manner and highlights previously unappreciated contributions of glial regulatory regions to neuropsychiatric risk.

2. **Reviewers 1 & 4 suggested to enhance disease relevance of the variant analysis.**

We also expanded our predictive modeling framework to include rare non-coding variants associated with neurodevelopmental disorders. Specifically, we analyzed variants from the Homozygosity Mapping Collaborative for Autism, NIMH, and Simons Simplex Collection cohorts that overlap human accelerated regions, VISTA enhancers, or conserved neural enhancers. While these variants were prioritized in both cases and controls, indicating no global case-control difference in regulatory impact, this analysis identifies individual case-associated variants with cell type-specific regulatory effects, highlighting their potential contribution to neurodevelopmental disorder risk.

3. Reviewers 2, 3 & 4 have requested to add functional validations.

We provide multiple layers of functional validation that establish causal roles for predicted regulatory elements. Cross-validation with a published massively parallel reporter assay demonstrates that a substantial fraction of predicted cis-regulatory elements are active in mid-gestation cortical cells, confirming their enhancer activity. Targeted shRNA-mediated knockdown of the transcription factor LHX2 in oRG altered transcriptional programs and differentiation trajectories, reducing self-renewal and promoting a shift toward an astrocyte-like state. CRISPR interference of the human accelerated region HARsv2_1313 demonstrated that it regulates ROCK2 expression and modulates proliferation in human neuronal precursors. These experiments confirm that specific predicted regulatory elements and transcription factors directly control gene expression programs and cell type–specific behavior during human cortical development.

Together, these additions substantially strengthen the manuscript by integrating external datasets, expanding disease-relevant analyses, and providing direct experimental support for predicted regulatory mechanisms.

In addition to these shared concerns, we have also addressed all other points raised by the reviewers. Below, we provide a point-by-point response letter, with the original reviewer comments in black and our responses in blue.

Referee #1 (Remarks to the Author):

The manuscript by Jones et al., has generated a comprehensive dataset for the investigation of genomic and epigenomic regulation in glial cell types in the mid-gestational developing human brain. In particular, the study applied a cell sorting scheme that can effectively separate the related vRG and oRG. Using the enriched cell populations, the authors generated three types of epigenomic profiles including ATAC-seq, WGBS, and PLAC-seq (or HiChIP). The data resource is complementary to published single cell datasets for two main reasons: 1) the published single cell HiC works have limited ability in distinguishing oRG from vRG. 2) the PLAC-seq data allows chromatin loops to be identified at a 2kb resolution, providing a greater spatial resolution than the single cell HiC approaches. The study took advantage of the VISTA enhancer resource in developing mouse brain, in addition to some new transgenic mice experiments to demonstrate that the identified cCREs are functional regulatory elements. The main weakness of the current manuscript is that the rich dataset was not fully explored through computational analyses. As discussed below, the manuscript did not fully explore PLAC-seq

contacts that do not overlap with a cCRE, which accounts for 75% of the contacts. The predictive modeling works that determine the effect of variants on cCREs can be expanded to rare variants associated with neurodevelopmental disorders. The authors also have an opportunity to integrate the data produced in this study with the dataset described in Song 2020 Nature (from the same groups of senior authors), to provide a more comprehensive view of the regulatory programs in the mid-gestational human brain.

We thank Referee #1 for the thoughtful and constructive evaluation of our manuscript and for recognizing the value of the cell type-specific epigenomic datasets generated in this study. We appreciate the reviewer's comments that the resource could be further explored computationally and have revised the manuscript substantially to address these suggestions and clarify several aspects of the analysis.

In response to the reviewer's comments regarding chromatin interactions that do not overlap cCREs, we performed additional analyses to evaluate the potential regulatory significance of these regions. We show that although some interacting bins overlap accessible or lowly methylated regions, distal bins lacking both features show little enrichment for neural VISTA enhancers, suggesting limited enhancer-like activity in neural lineages. We also clarified the regulatory interpretation of the PTPRG locus and expanded the description of the broader interaction landscape at this gene.

To further extend the genetic analyses, we now compare disease heritability enrichment between interacting and non-interacting accessible regions and integrate datasets from Song et al. In addition, we expanded our predictive modeling framework to include rare non-coding variants associated with neurodevelopmental disorders, including variants from the Simons Simplex Collection. These analyses identify rare variants predicted to perturb regulatory activity in a cell type–specific manner.

Together, these revisions expand the computational exploration of the dataset, clarify key interpretations, and strengthen the manuscript's conclusions. We thank Referee #1 again for the helpful suggestions that significantly improved the manuscript.

Major comments:

1.The manuscript focused on PLAC-seq loops overlapping with cCREs. But these only account for ~25% of loops. <u>The functional significance of chromatin loops not overlapping with a distal cCRE is not been sufficiently explored</u>. One such example is PTPRG shown in Fig. 2f. Although the discussion focused on VISTA enhancers hs434 and hs435, these two elements are relatively weakly connected to PTPRG. What is the potential regulatory significance of other regions connecting to the gene, if they are not cCREs?

We thank Referee #1 for raising the important point that only a subset (~25%) of PLAC-seq bin-to-bin interactions overlap distal cCREs, and for encouraging further consideration of the functional relevance of interactions that do not meet our cCRE criteria.

In this study, we deliberately focused on a stringent subset of interacting regions in Fig. 2b to enable a conservative and interpretable functional analysis. Specifically, distal cCREs were required to be distal to promoters, not overlap H3K4me3 signal, and to be both accessible and lowly methylated. Under these criteria, a substantial fraction of interactions fall into the "other" category. Importantly, however, ~15-21% of these "other" interactions still overlap regions that are either exclusively accessible or lowly methylated (LMRs), as shown in the table below, suggesting that a subset may retain regulatory potential despite not meeting our full cCRE definition.

		cCREs	LMR / Accessible	Closed	Total
vRG	XOR interactions	25,129	15,721	62,842	103,692
	Percentage	24.23%	15.16%	60.60%	100.00%
oRG	XOR interactions	26,913	15,907	67,721	110,541
	Percentage	24.35%	14.39%	61.26%	100.00%
OPC	XOR interactions	23,296	18,973	64,741	107,010
	Percentage	21.77%	17.73%	60.50%	100.00%
MG	XOR interactions	27,209	21,946	52,589	101,744
	Percentage	26.74%	21.57%	51.69%	100.00%

Table of the amount of distal cCREs (lowly methylated and accessible chromatin), lowly methylated or accessible chromatin (LMR/Accessible), or closed chromatin participating in XOR (H3K4me3-to-distal) interactions for each cell type.

We fully agree that chromatin interactions not overlapping distal cCREs may nevertheless be functionally relevant. Such regions could act as silencers, insulators, structural elements, or components of higher-order regulatory hubs, roles that are not readily inferred from accessibility and methylation alone. Computationally assigning function to these regions is challenging, as they often occur within "clusters" of interactions that include cCREs, complicating direct attribution.

To address this concern, we expanded our VISTA enhancer enrichment analysis to explicitly test whether distal interacting bins that do not overlap either accessible or lowly methylated chromatin ("Closed" in Table) show evidence of enhancer activity. We filtered both neural VISTA enhancers (n = 1,233; defined by activity in neural tube, forebrain, midbrain, hindbrain, dorsal root ganglion, cranial nerve, or trigeminal tissues) and negative VISTA elements (n = 1,868; completely inactive) to exclude overlap with accessible or lowly methylated regions, accounting for the fact that VISTA elements can span multiple 2 kb bins. Under these conditions, we observed no enrichment of neuronal enhancers among distal interacting regions in vRG or oRG. We did detect a weak negative association in OPCs (OR = 0.39, P = 0.04) and a weak positive association in microglia (OR = 2.18, P = 0.027), suggesting limited and cell type-specific effects. Overall, these results indicate that distal interacting bins lacking accessibility or low methylation show minimal enhancer-like activity in neural lineages.

Conditon	cCRE_Neu	cCRE_Neg	Control_Neu	Control_Neg	% overlapping hits	% control overlapping hits	OR	Low	High	Pval
vRG	17	74	13	74	18.68%	14.94%	1.305722	0.5522532	3.151252	0.55
oRG	22	80	27	76	21.57%	26.21%	0.7750469	0.3845987	1.5487963	0.513
OPC	9	71	21	65	11.25%	24.42%	0.3945231	0.1478416	0.9768245	0.04234
MG	30	43	21	66	41.10%	24.14%	2.181664	1.056348	4.576743	0.02701

Table of odds ratios of the association of interacting bins not overlapping accessible or lowly methylated chromatin with positive neural VISTA elements or negative neuronal VISTA elements.

Regarding Fig. 2f, we highlighted two distal cCREs within validated VISTA enhancers, hs434 and hs435, which interact with the PTPRG locus over a distance exceeding 800 kb in vRG. While the Referee notes that these elements appear weakly connected relative to other interactions, the interactions with hs434 are significant (FDR = 1.22×10^{-5} in vRG and 1.63×10^{-5} in oRG), ranking stronger than 60 and 82 other significant interactions involving PTPRG in the respective cell types. In total, PTPRG participates in 102 and 168 interactions encompassing 31 and 51 cCREs across 10 and 15 interaction clusters in vRG and oRG, respectively, indicating a complex regulatory landscape. Many additional regions contacting PTPRG are also cCREs, and we agree that these elements may be functionally important.

We specifically highlighted hs434 and hs435 because they are independently validated enhancers in the VISTA database, serving as a proof-of-principle that our approach can recover long-range regulatory interactions. We agree with Referee #1 that other interacting regions at this locus may also contribute to gene regulation.

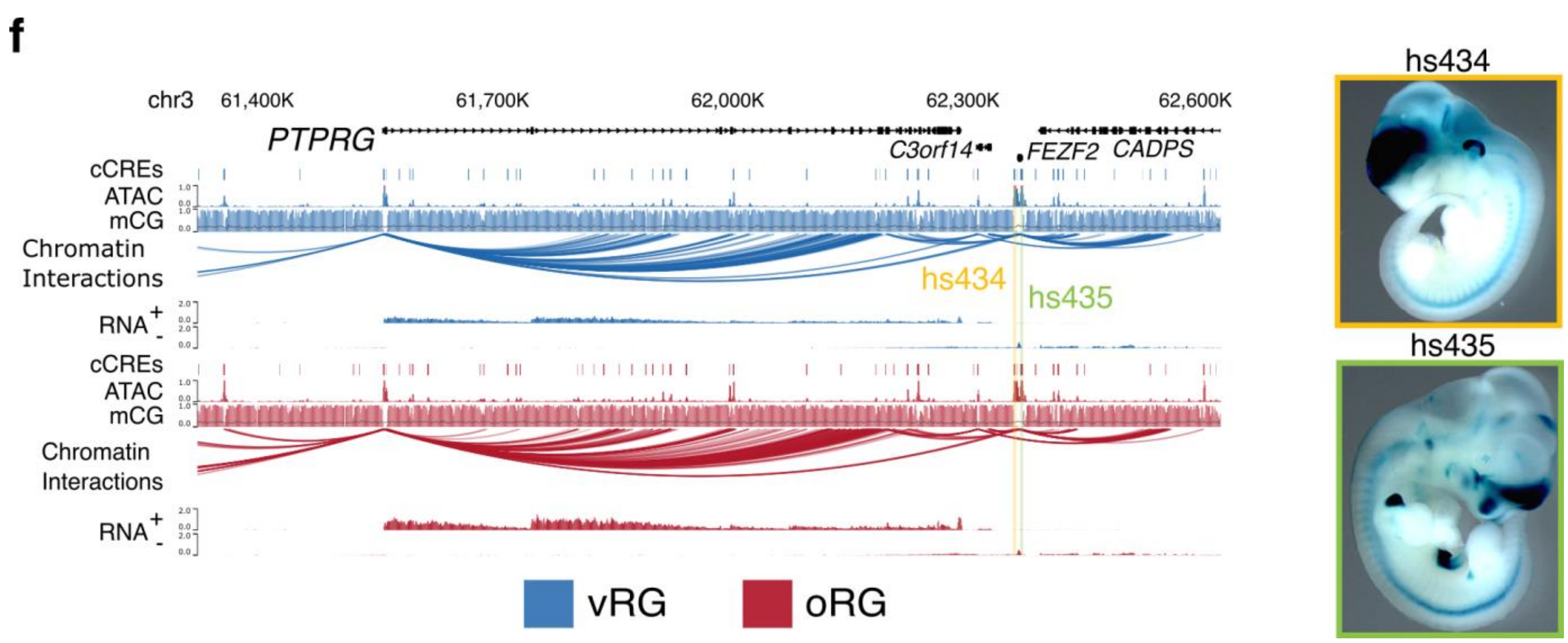

Figure 2: f) Browser session of VISTA element hs434 (light yellow highlight) and hs435 (light green highlight) shown to interact with PTPRG in both vRG (blue) and oRG (red).

2. In line 134, the manuscript stated that more than two-thirds of interaction clusters contain a cCRE. But 15 kb is a large space, and no statistics were done to support the observation was more significant than the random distribution of cCRE.

The difference between the overlap of interactions (Ext. Fig. 3d) and clusters (Ext. Fig. 3e) with cCREs is contributed by the filtering criteria: for the interaction analysis, we focused on XOR (H3K4me3-to-distal) interactions exclusively and assessed cCREs that overlap within the distal region, whereas the cluster analysis included all interactions, AND, and XOR. For example, there may be a strong interaction between two H3K4me3 bins, but the surrounding non-accessible bins are also deemed to be significantly interacting. As such, when overlapping cCREs with either bin of all interactions, 95.4%-96.6% of them contain a cCRE.

We chose to use a 15kb distance to define clusters as suggested by MAPS (PMID 30986246). We removed Ext. Fig. 3e and f for clarity, as Fig. 2b and Ext. Fig. 3e both demonstrate that cCREs participate in interactions with 6.7-13% greater average normalized contact frequency (observed/expected counts) than non-cCRE elements.

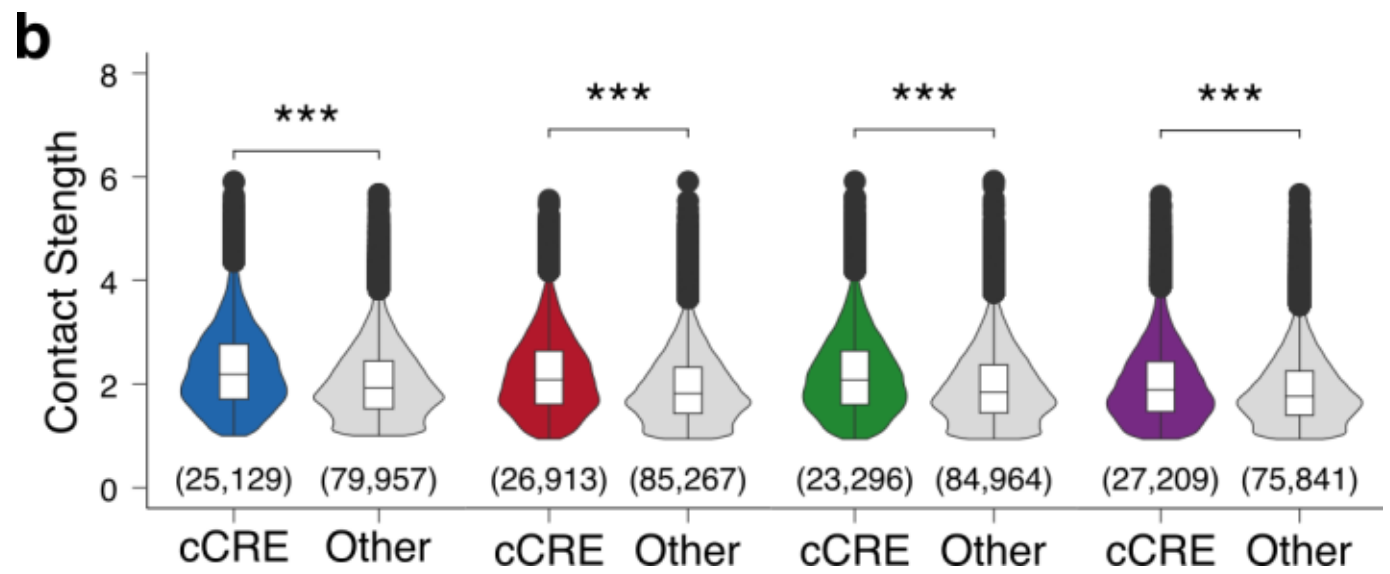

Figure 2: b) Violin plots of the contact strength log2(observed/expected counts) in XOR interactions with cCREs and without cCREs in the distal bin (two-sided t-test, *: P < 0.05, **: P < 0.01, ***: P < 0.001).

3.Why do the regions shown in Fig. 2c show such a strong specificity for chromatin contact frequency? Were the regions selected for high chromatin contact only in one cell type? Alternatively, does the result suggest chromatin contact being the most sensitive data modality for distinguishing the cell types?

We thank the reviewer for this question and for the opportunity to clarify the rationale underlying Fig. 2c. The strong cell type-specificity observed in chromatin contact frequency in this figure reflects, in part, the intentional selection of regions and interactions.

Specifically, the interactions shown in Fig. 2c were first filtered to be cell type-specific based on the binary significance of interactions, as shown in Ext. Fig. 4d. Only interactions significant in one cell type were retained. These interactions were then further filtered to require overlap of at least 50% with a cCRE within the distal bin, as described in the "Heatmap of cell type-specific cCREs" section of the Methods. As a result, the regions displayed in Fig. 2c are enriched for loci with pronounced cell type-specific 3D genome organization by design.

Therefore, Fig. 2c does not imply that chromatin contact frequency is intrinsically the most sensitive data modality for distinguishing cell types. Rather, the figure highlights the strong concordance between cell type-specific chromatin interactions and cell type-specific chromatin accessibility, DNA methylation, and gene expression at these selected loci.

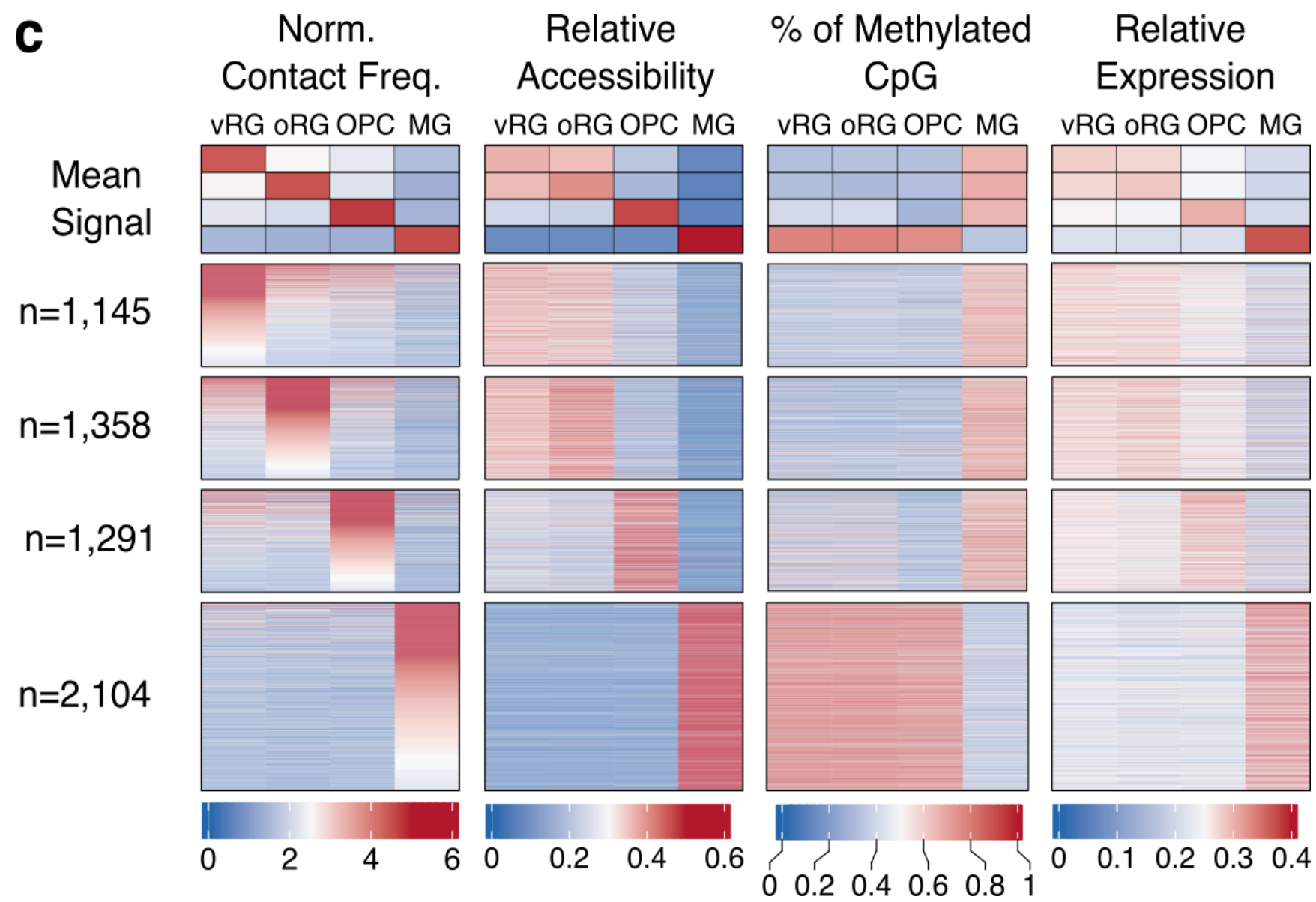

Figure 2: c) Heatmap displaying normalized contact frequencies, chromatin accessibility, CpG methylation percentage, and target gene expression of cell type-specific XOR interactions with cCREs in the distal bin.

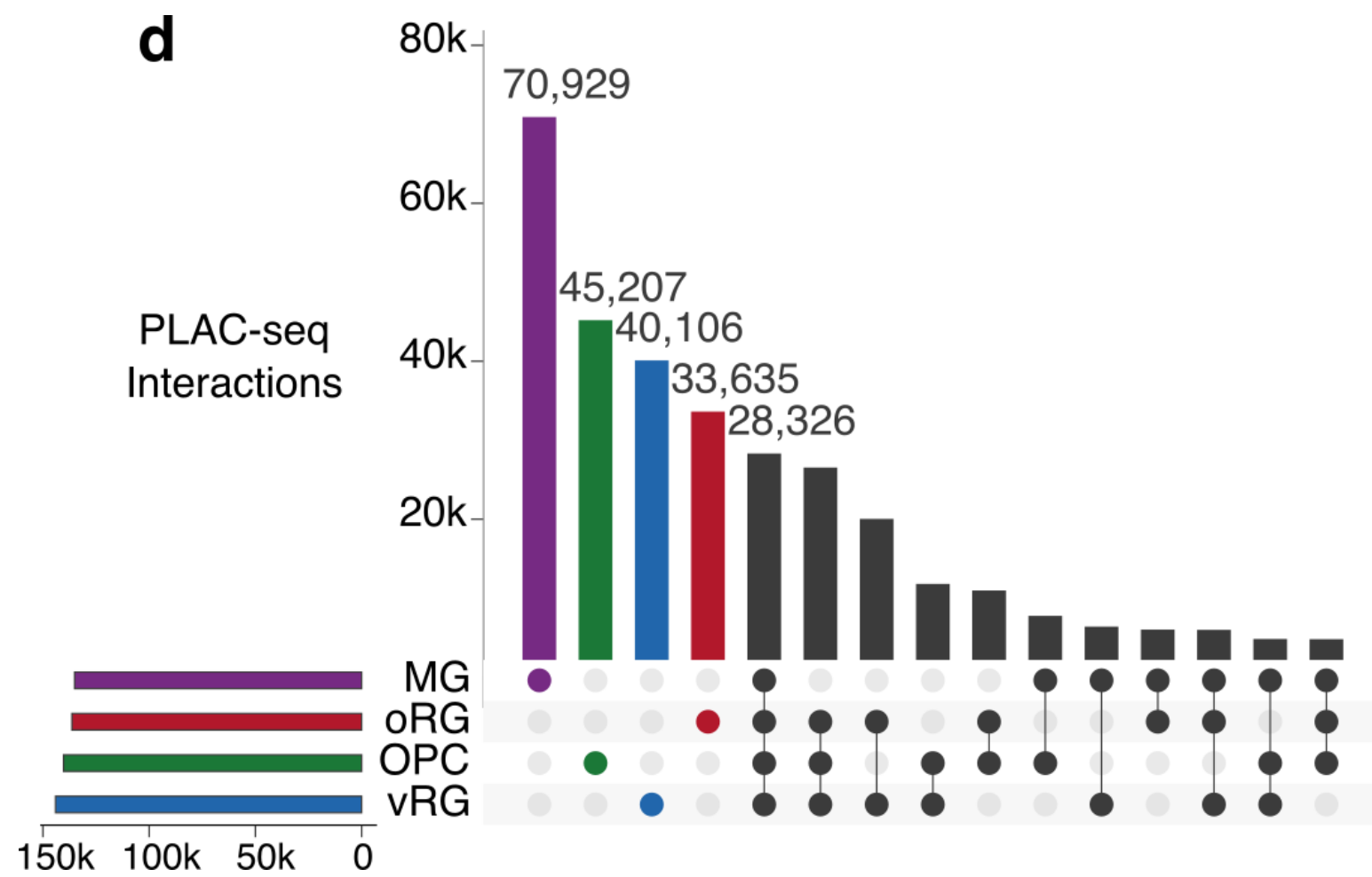

Extended Figure 4d: Upset plot of both AND and XOR PLAC-seq interactions.

4.Is it surprising to find ASCL1 as a major TF driving the distinction between dorsal vRG and oRG? To my knowledge, ASCL1 has critical roles in inhibitory neuron differentiation.

We thank the reviewer for this thoughtful question. ASCL1 is well known for its critical role in inhibitory neuron differentiation. Recent findings from our group and others have shown that both vRGs and oRGs in the cortex possess the capacity to generate inhibitory lineage cells (PMIDs 34912114, 37673072, 39779846). Notably, it has been proposed that vRGs have a greater propensity to generate inhibitory neurons than oRGs (PMID 39023844). Our current results further support this notion, highlighting intrinsic differences in lineage potential between these two radial glia populations.

5.The manuscript compared the enrichment of disease heritability at interacting cCREs and all interacting 2kb bins. Did the authors also compare to non-interacting cCREs?

In the initial submission, we did not explicitly compare interacting cCREs to non-interacting cCREs. We have now performed this additional analysis.

To enable direct comparison with the datasets used in Song et al., we repeated the heritability enrichment analysis using accessible chromatin peaks. We stratified peaks into those participating in significant 3D chromatin interactions and those that do not. We found that accessible peaks involved in 3D interactions are enriched for neuropsychiatric disease heritability in both glial and neuronal cell types. In contrast, non-interacting accessible chromatin shows enrichment for neuropsychiatric traits exclusively in glial cell types.

These results (described at line 266) indicate that chromatin interactions refine disease-relevant regulatory elements, particularly in neuronal lineages, whereas a subset of non-interacting accessible regions in glial cells may still contribute to disease risk through interaction-independent regulatory mechanisms. We have incorporated these results into the revised manuscript.

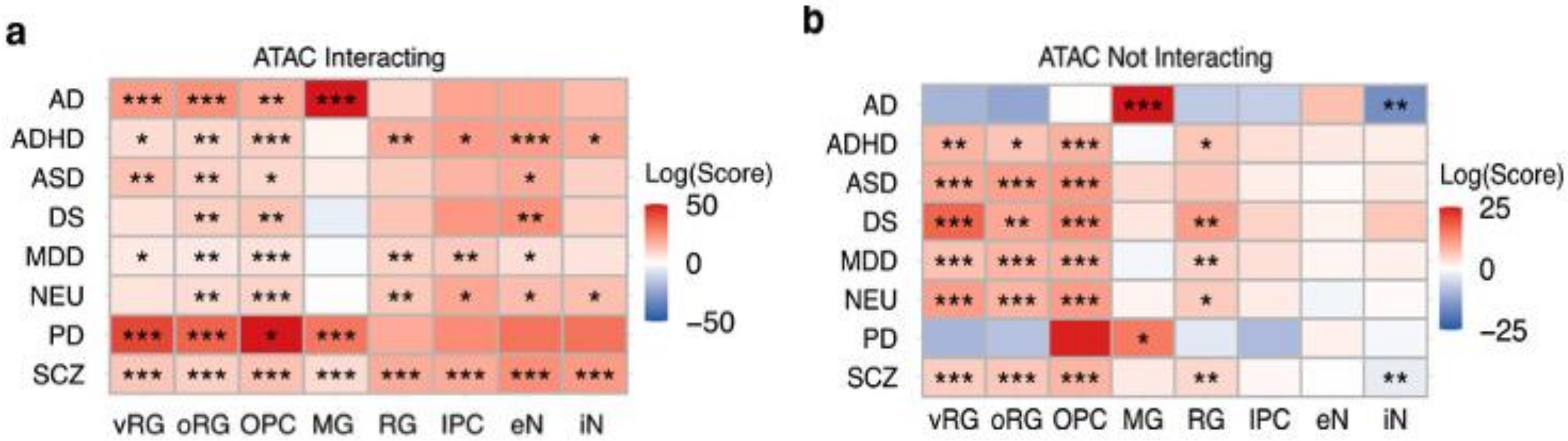

Ext. Fig. 10: Heatmap of LDSC score for (a) ATAC-seq peaks involved in 3D interaction and (b) ATAC-seq peaks not involved in 3D interactions. Red, the disorder is positively enriched. Blue, the disorder is negatively enriched. (*: P < 0.05, **: P < 0.01, ***: P < 0.001). Note that the high yet insignificant enrichment score for PD in eN was driven by variants in the *MAPT* locus.

6.The footprinting-based approach for analyzing TF binding may not be distinguish the binding signature of TCF4 and TCF12 , which share a CACGTG binding motif. Such a caveat should be discussed in the manuscript.

We thank the reviewer for highlighting this important limitation. We agree that footprinting-based approaches cannot reliably distinguish binding events between transcription factors that share highly similar or identical DNA-binding motifs, such as TCF4 and TCF12.

We have now explicitly discussed this caveat in the revised manuscript (Discussion, line 402). We note that although transcription factor binding sites were filtered based on expression of the corresponding TFs, foot printing signal at shared motifs likely reflects motif family activity rather than unambiguous binding by a specific factor.

7.The predictive modeling of the effect of variants on cCREs may be expanded to rare variants such as those identified by the Simons Simplex Collection for autism, or rare variants associated with other types of neurodevelopmental disorders.

We appreciate the feedback to extend our analysis to rare variants. We expanded our predictive modeling framework to include rare non-coding variants associated with neurodevelopmental disorders, as suggested. Specifically, we analyzed rare variants from the Homozygosity Mapping Collaborative for Autism, NIMH, and Simons Simplex Collection (SSC) cohorts that occur within human accelerated regions (HARs), VISTA enhancers, or conserved regions previously predicted to function as neural enhancers (PMID 39019033). These additions are described in the revised manuscript (line 287).

723 of 1,235 rare variants were accessible in one of the cell types, with 61 predicted to perturb accessibility (Ext. Fig. 10f). The variants were identified in cases and controls (167 case-specific, 509 control-specific, and 47 shared) and were predicted to perturb accessibility to a similar extent across all cell types, indicating no global case-control difference in regulatory impact. (Ext. Fig. 11e). Instead, this approach resolves individual case-associated variants with cell type-specific effects, such as a variant in HAR0366 (chr11:31246972:C>T) predicted to disrupt accessibility in vRG and oRG and to interact with the ASD-associated transcription factor PAX6 (PMID 21203536) (Ext. Fig. 11f).

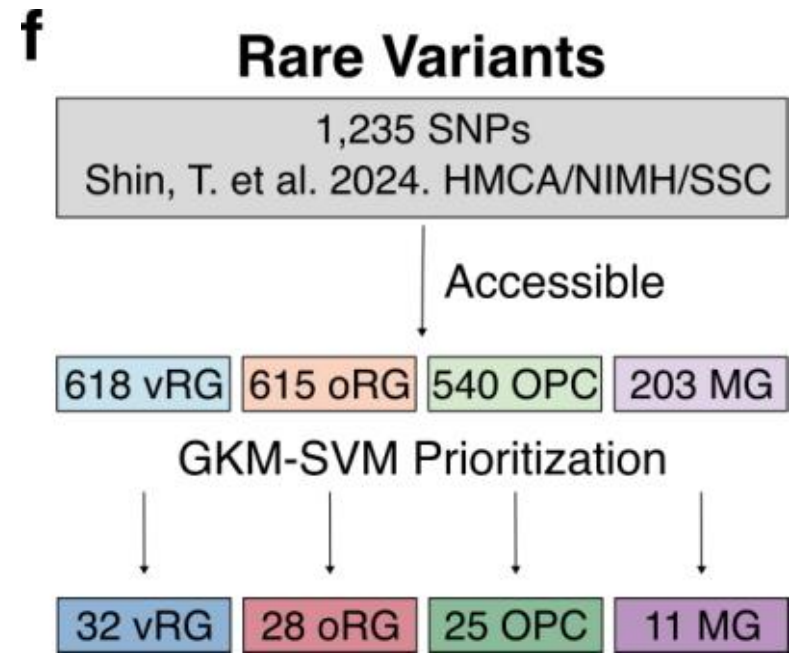

Ext. Fig. 10: f) Schematic of variant prioritization for Rare Variants 1) Credible SNPs accessible in each cell type. 2) SNPs predicted to significantly disrupt accessibility compared to shuffled controls.

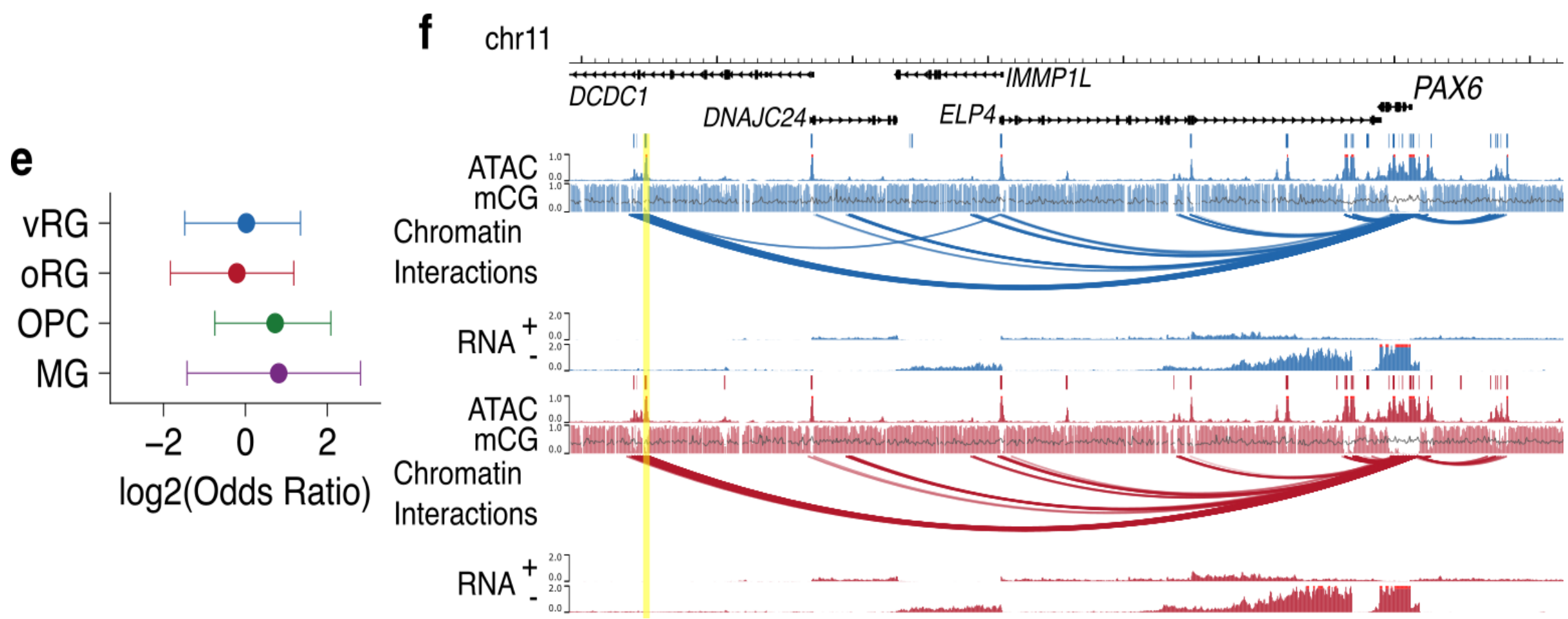

Ext. Fig. 11: e) Forest plot showing the association of rare variants with ASD cases. Data are shown as odds ratios with 95% confidence intervals. f) WashU browser of the locus containing HAR0366.

8. Although this study focused on the distinction between glial cell types. It seems natural to integrate the Song 2020 dataset and the newly generated dataset to better understand the regulatory dynamics from vRG to oRG to newborn neurons.

We agree that the Song 2020 dataset provides a valuable reference for comparison. Direct integration, however, is challenging due to differences in data quality and resolution: our PLAC-seq data captures 135-144k interactions at 2 kb resolution, whereas Song 2020 reports 22-35k interactions at 5 kb resolution. Additionally, our definition of cCREs leverages methylation data, which is not available in the Song 2020 dataset, further limiting direct comparisons.

Despite these limitations, we incorporated the Song 2020 dataset in two complementary ways. First, we extended the genetic heritability analysis using LDSC to include accessible peaks from radial glia (RG), intermediate progenitor cells (IPC), excitatory neurons (eN), and inhibitory neurons (iN) from Song 2020 (Ext. Fig. 9), enabling a broader comparison of disease relevance across developmental stages. Second, we included Song 2020 ATAC-seq data when testing the enrichment of human accelerated regions (HARs) overlapping cell type-specific regulatory elements. This analysis confirmed that the enrichment of HARs overlapping oRG regulatory elements was not exclusive to the cell populations within our study, but remained when data from Song 2020 was integrated (Ext.Fig. 13).

Together, these analyses partially address the reviewer's suggestion by integrating Song 2020 data where data and methodological compatibility permits. However, a full joint analysis of 3D regulatory dynamics from vRG to oRG to newborn neurons is limited by differences in data resolution and available epigenomic modalities and would require generation of matched datasets across all lineages.

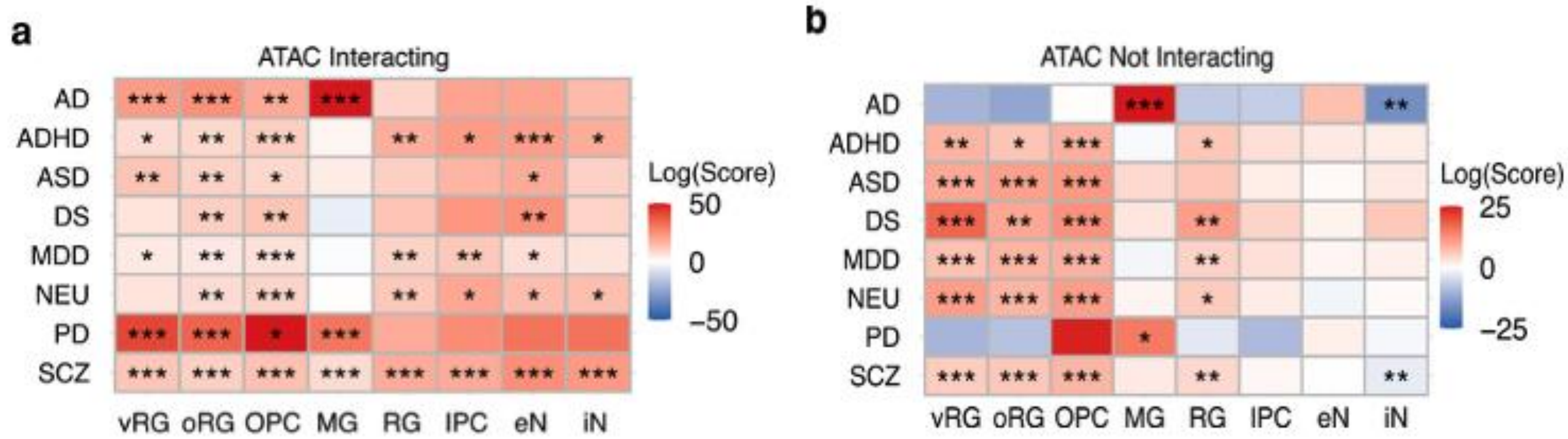

Ext. Fig. 10: Heatmap of LDSC score for (a) ATAC-seq peaks involved in 3D interaction and (b) ATAC-seq peaks not involved in 3D interactions. Red, the disorder is positively enriched. Blue, the disorder is negatively enriched. (*: P < 0.05, **: P < 0.01, ***: P < 0.001).

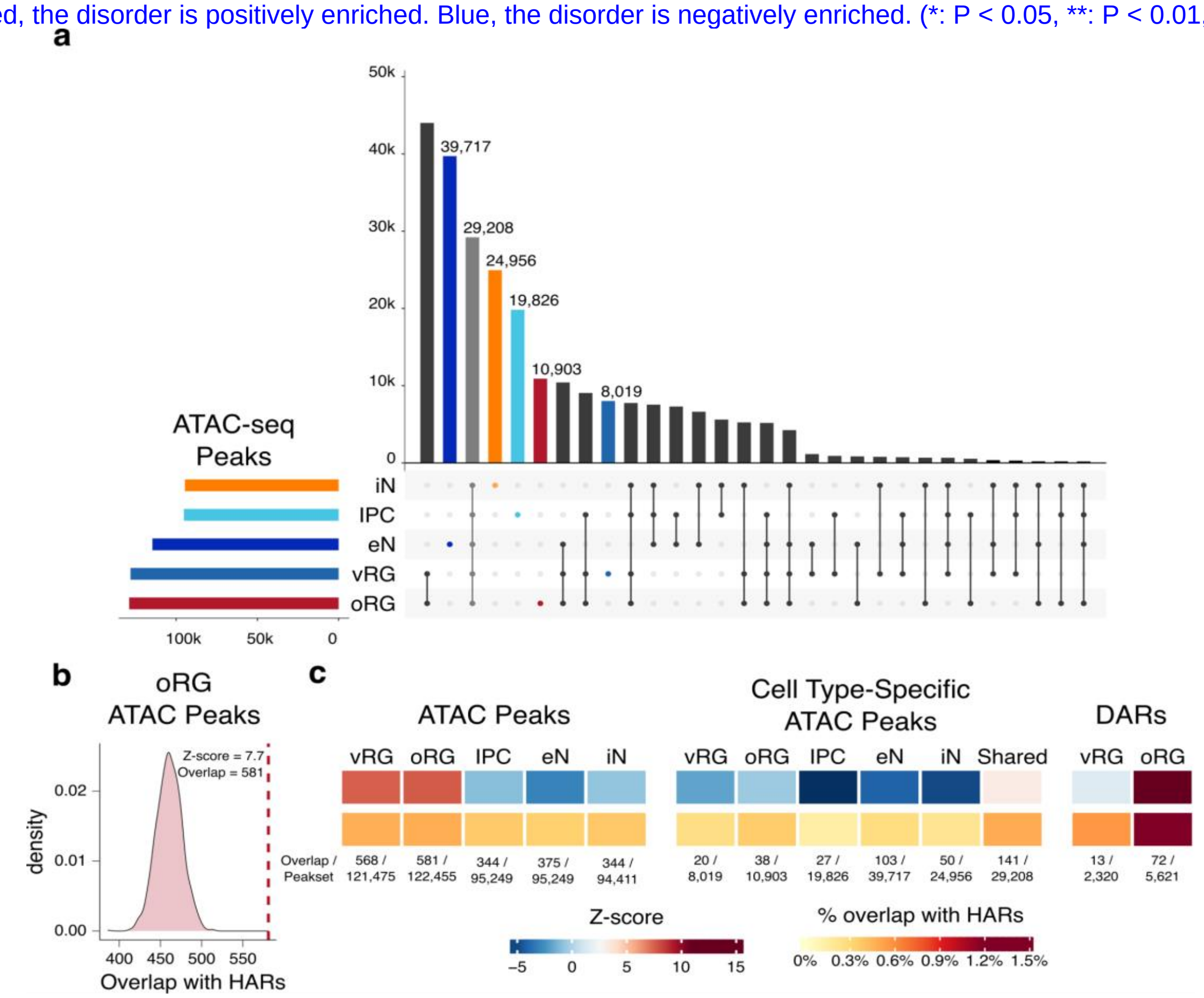

Ext. Fig 13: a) Upset plot of vRG, oRG, IPC, iN, and eN ATAC-seq peaks b) Density plot of randomly sampled cCREs (n=1,000) overlapping with HARs. Dotted line marks the amount of overlap of cell type-specific cCREs with HARs. c) Heatmap displaying the z-score (top) for overlap of either ATAC peaks, cell type-specific ATAC Peaks, or vRG and oRG DARs compared with a randomly sampled empirical null distribution, as well as the percentage of HARs overlapping the data (bottom).

Referee #2 (Remarks to the Author):

This paper by Jones et al is one of the very first to study in a 'multiomic' approach multiple glial cell types in the human fetal brain , with the critical element of FACS-sorting/separating outer radial glia (oRG) from ventricular radial glia and comparing these two cell types. the oRG are thought to play a critical role in primate brain evolution.

Major Comment:

Figure 5 is the most exciting result of the paper, showing that cis-regulatory elements specific to the oRG are enriched for human accelerated (HAR) DNA sequences. Could the authors add a cell function-based experiment, some sort of cell biology or related assay to show for at least one of their oRG cis-regulatory

elements at an HAR, how it modifies oRG function and/or how it could contribute to some of the unique features of the human brain?

We greatly appreciate Referee #2's enthusiasm for our finding that oRG cCREs are enriched for human accelerated regions (HARs). In response to the suggestion to provide functional validation, we initially attempted to perturb HARsv2_1602 (Fig. 5), using CRISPR interference (CRISPRi) in human iPSC-derived neural progenitor cells (NPCs) (described in methods at line 1006) to assess its regulation of EPHA4. Prior work (PMID 25978062) demonstrates that loss of EPHA4 leads to a reduced neonatal cortical size in mice, consistent with the gene ontology of our prioritized HAR target genes, including "system development" and "cell population proliferation." Unfortunately, CRISPRi perturbation of HARsv2_1602 did not decrease EPHA4 expression in NPCs, likely reflecting differences in the regulatory epigenomic landscape between primary oRGs and the iPSC-derived NPCs used for validation, as shown in the figure below.

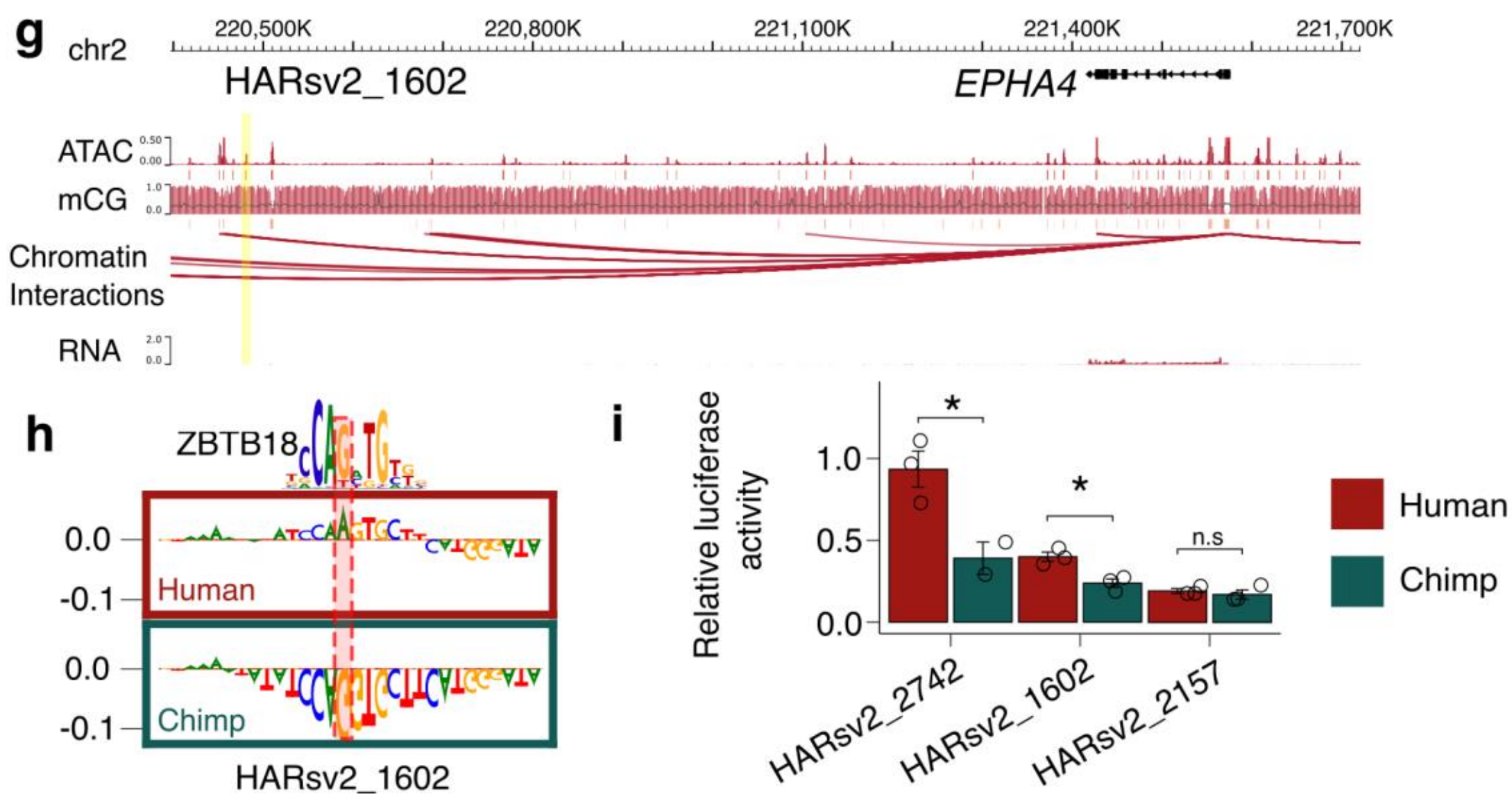

Figure 5: g) WashU browser of the *EPHA4* locus. HARv2_1602 highlighted in yellow. h) Overview of HARsv2_1602. Top, ZBT18 motif that is predicted to be perturbed. Middle, GkmExplain importance scores for each base pair surrounding the human A allele. Bottom, GkmExplain importance scores for each base pair surrounding the chimpanzee ancestral G allele i) Relative luciferase activities compared to minimum promoter of HARs predicted to be more accessible than chimp orthologs (two-sided t-test, n.s: not significant, *: $P < 0.05$, **: $P < 0.01$, ***: $P < 0.001$).

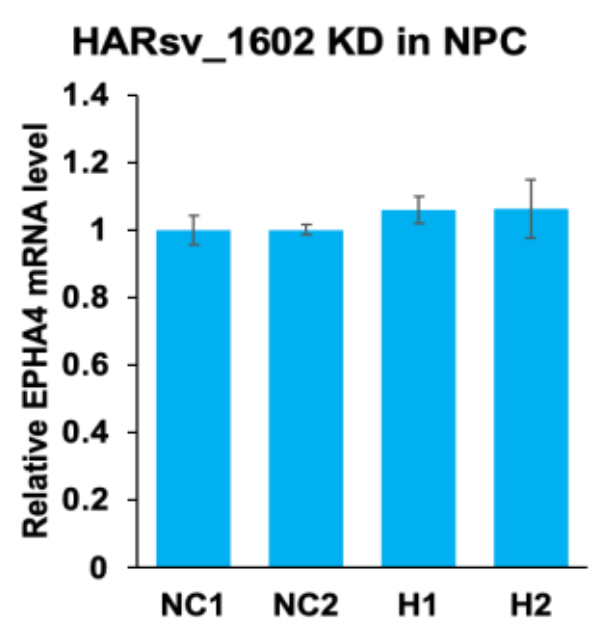

Expression of EPHA4 in NPC based on RT-qPCR. Negative Control (NC). HARv2_1602 targeting guides (H).

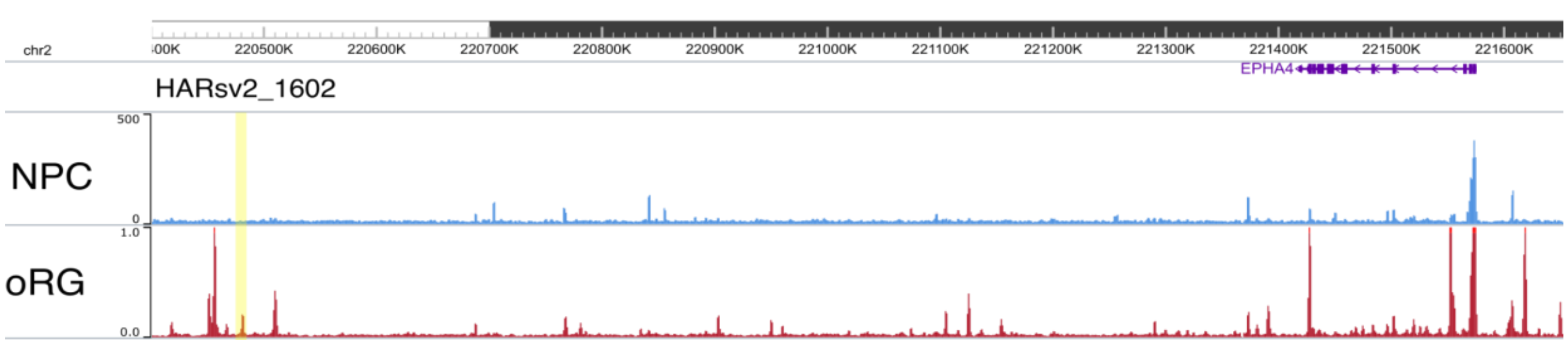

WashU browser of the EPHA4 locus. HARv2_1602 highlighted in yellow. NPC signal obtained from PMID 38297124.

Given these limitations, we focused on validating HARsv2_1313 (line 338). The human-specific G allele at chr2:11,301,810 is predicted to reduce chromatin accessibility relative to the ancestral A allele in oRGs (Fig

5c). HARsv2_1313 interacts with the promoter of ROCK2 (Fig. 5d). CRISPR interference (CRISPRi) using paired guide RNAs (gRNAs; Table 10) in human iPSC-derived neuronal precursor cells (NPCs) demonstrated decreased ROCK2 expression, confirming HARsv2_1313 functions as an enhancer of ROCK2 (Fig. 5e). Consistent with prior evidence that ROCK inhibition promotes stem cell proliferation and viability (PMID 19235204), we observed a significant increase in Ki67-positive cells in EMX+ NPC population following knockdown of the ROCK2 promoter and HARsv2_1313 with one pair of gRNAs, whereas the second pair showed a similar trend toward increased proliferation (Ext. Fig. 14d). Moreover, the percentage of Ki67 was positively correlated with the gRNA-associated green fluorescent protein (GFP) signal in all conditions relative to control (slope difference = 3.48-5.86, P < 0.001) (Fig. 5f). Together, these results suggest that reduced HARsv2_1313 activity in humans decreases ROCK2 expression and increases proliferation, in agreement with prior CRISPR activation experiments in iPSC-derived neurons (PMID 36640767) and with higher ROCK2 expression in chimpanzee organoids compared to human organoids (PMID 30735633).

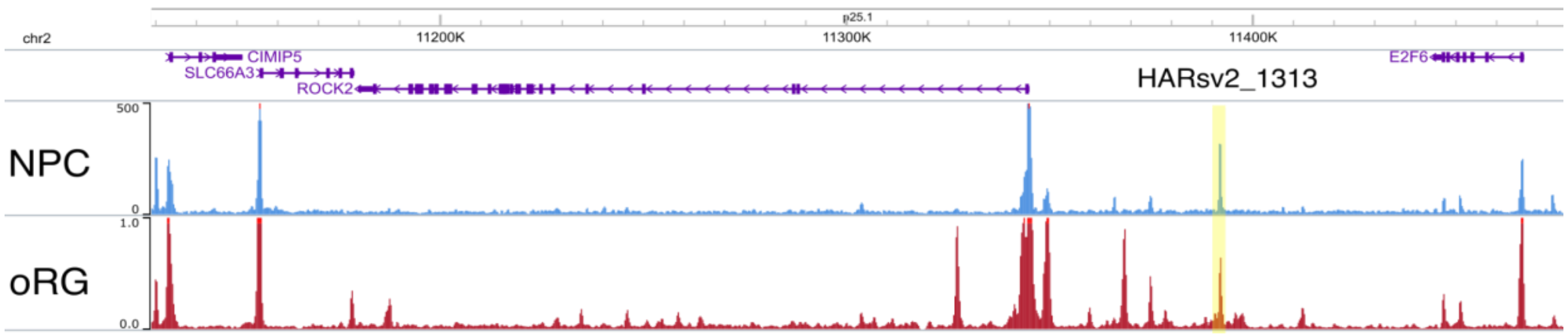

WashU browser of the ROCK2 locus. HARv2_1313 highlighted in yellow. NPC signal obtained from PMID 38297124.

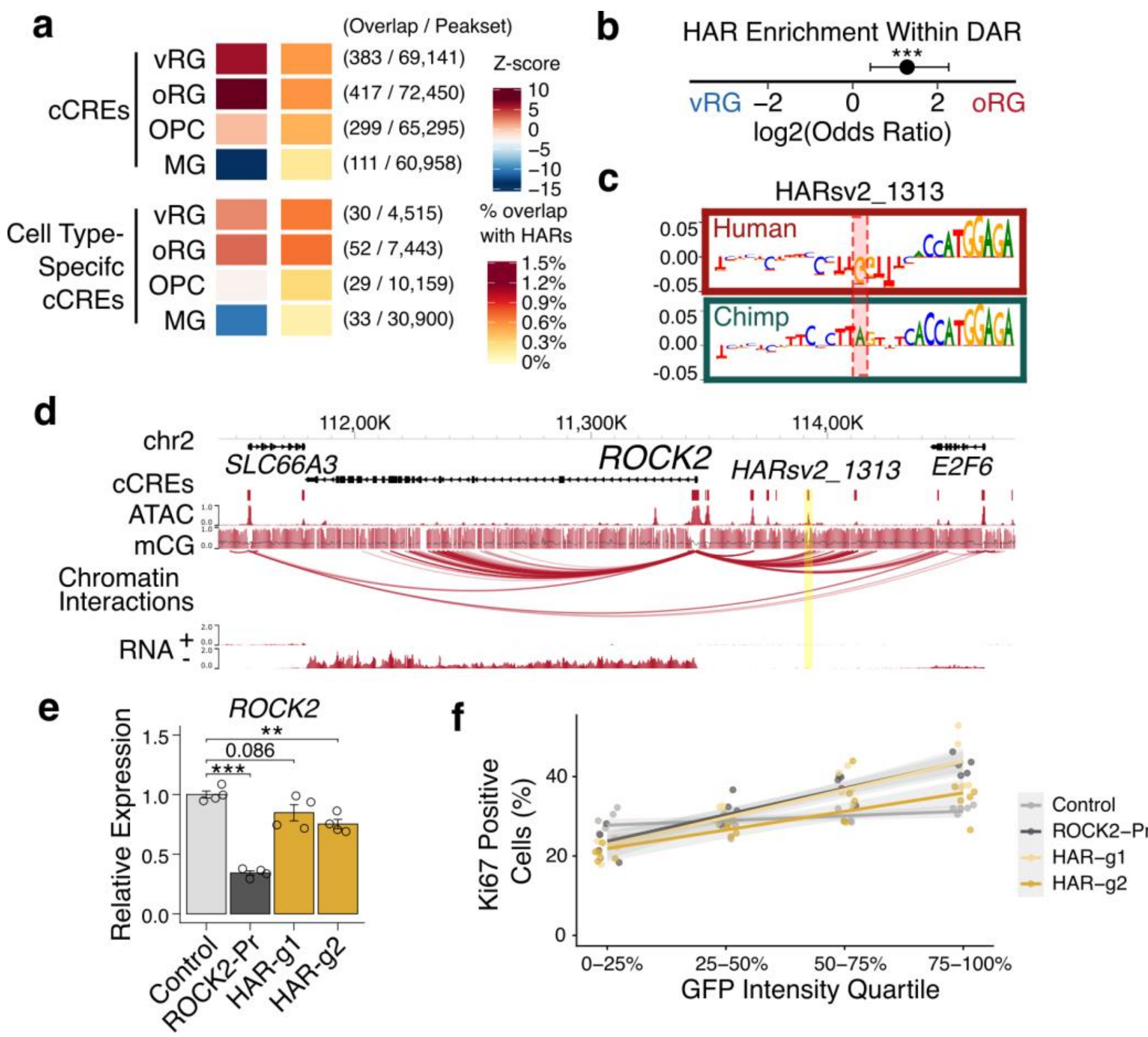

Figure 5: a) Heatmap displaying the z-score (left) for overlap of either cCREs or cell type-specific cCREs compared with a randomly sampled empirical null distribution as well as the percentage of HARs overlapping the data (right). b) Forest plot showing the association of vRG or oRG DARs with HARs. Data are shown as odds ratio with 95% confidence interval (Fisher's exact test, *: P < 0.05, **: P < 0.01, ***: P < 0.001). c) Overview of HARsv2_1313. Top GkmExplain importance scores for each base pair surrounding the human G allele. Bottom, GkmExplain importance scores for each base pair surrounding the chimpanzee ancestral A allele. d) WashU browser of the ROCK2 locus. HARv2_1313 highlighted in yellow. e) Expression of ROCK2 in NPC based on RT-qPCR. Four independent differentiations per condition were used. (two-sided t-test, *: P < 0.05, **: P < 0.01, ***: P < 0.001). f) Percentage of cells positive for Ki67+ in NPC based on immunocytochemistry stratified into quartiles by GFP intensity. Linear regression lines are shown with 95% confidence intervals.

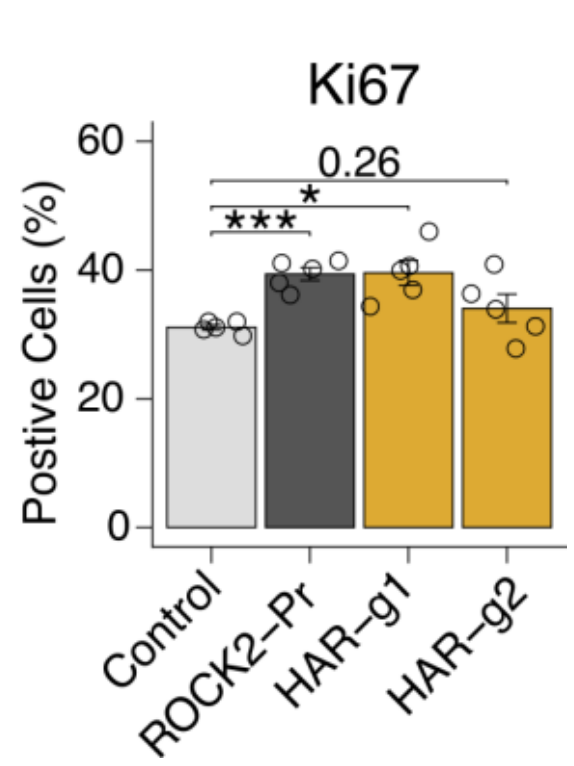

Figure 14: d) Percentage of cells positive for Ki67+ in NPC based on immunocytochemistry. Five independent differentiations per condition were used (two-sided t-test, *: P < 0.05, **: P < 0.01, ***: P < 0.001).

Minor:

The cell-specific differences in the figure 2f chromosomal contact map are very hard to decipher. perhaps adding differential contact map/track may help.

We thank the reviewer for this suggestion. The primary purpose of Figure 2f was not to highlight differential contacts, but to illustrate that VISTA elements participate in chromatin interactions in both vRG and oRG cells. To improve interpretability, we have remade the figure using solid colors for the 3D interactions, making the contacts easier to visualize. We also have a WashU genome browser session available in the Data Availability Statement, which allows readers to better view genome-wide contact information.

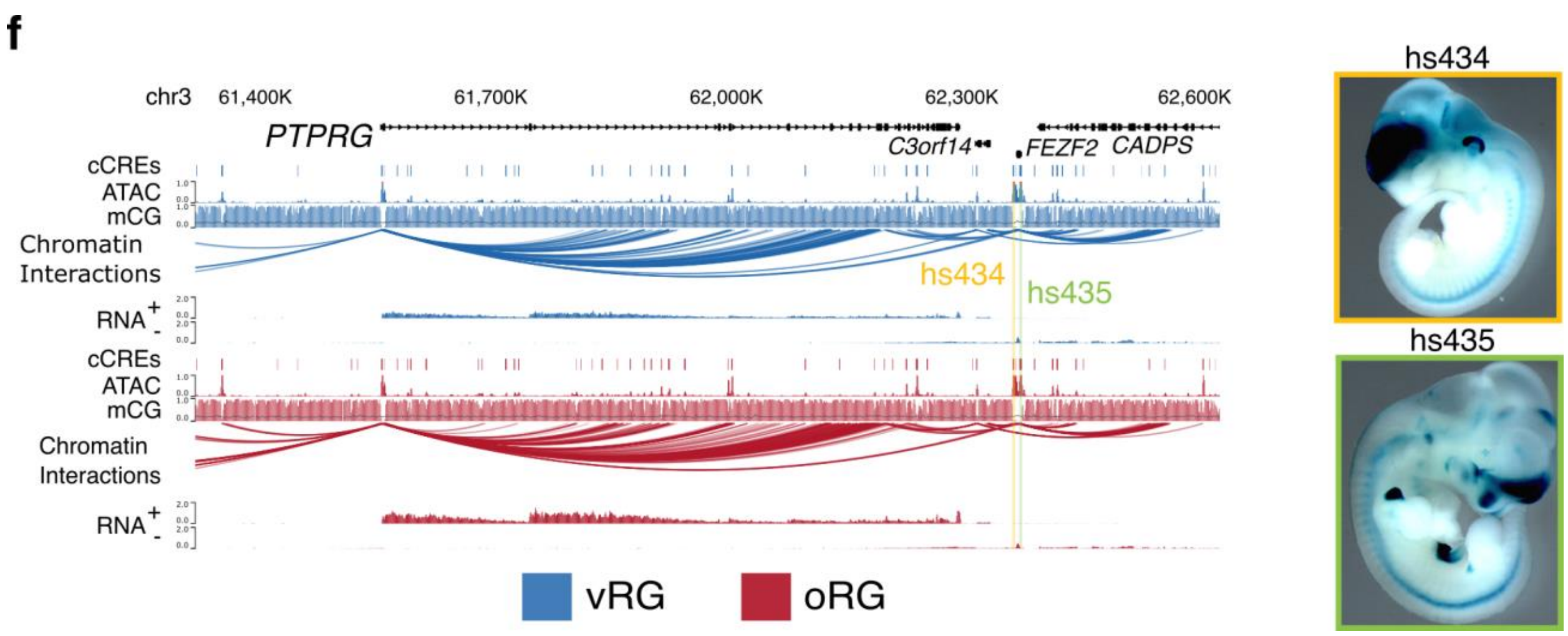

Figure 2: f) Browser session of VISTA element hs434 (light yellow highlight) and hs435 (light green highlight) shown to interact with PTPRG in both vRG (blue) and oRG (red).

Technical comment:

figure 1a HOPX marker-based FACS does not produce two separate clouds for vRG and oRG; while I believe the authors that they are successfully enriching/separating ensembles of vRG and oRG, I feel a standard single cell(nuclei) 10x genomics or similar single cell RNA-seq assay as quality control check for this key experiment would greatly benefit the paper.

We thank Referee #2 for thoroughly reviewing our sorting strategy. We agree that the HOPX marker-based strategy does not produce two distinct populations, as it reflects a continuous range of expression during corticogenesis. Indeed, prior scRNA-seq (PMID 29217575) and staining (PMID 26406371) have shown HOPX is preferentially, but not exclusively, expressed in oRGs. To further confirm and validate our sorting strategy, we utilized the CIBERSORTx program to compare our bulk RNA-seq data with matching scRNA-seq (PMID 29217575). Our results in Ext. Fig. 1e highlight successful enrichment of vRG vs oRG using the current sorting strategy.

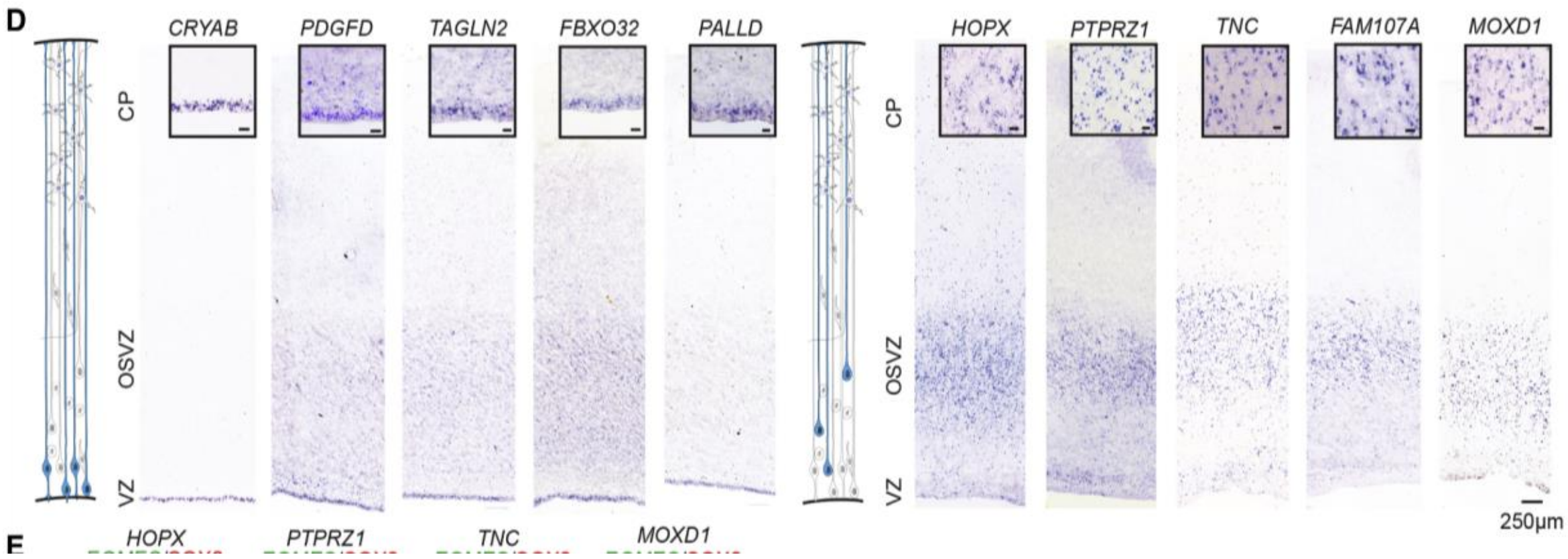

PMID 26406371: D) Representative examples of in situ hybridization staining for candidate vRG and oRG markers in human cortical tissue sections at GW18.2. The inset shows a higher magnification of the positively stained region (scale bar 25µm).

Referee #3 (Remarks to the Author):

In their manuscript entitled "3D Epigenome of Glial Cell Types in Developing Human Cortex", Jones, Wang et al combine transcriptomic and epigenomic analyses of four major glial populations in the mid-gestational human neocortex (ventricular radial glia (vRG), outer radial glia (oRG), oligodendrocyte precursor cells (OPC), and microglia (MG)). To do this, they generated high-resolution RNAseq, ATACseq, DNA methylation, and PLAC-seq datasets of sorted vRGs (EOMES-, HOPXlow), oRGs (EOMES-, HOPXhi), microglia (PU.1+), and oligodendrocytes (OLIG2+) purified from second-trimester cortical samples. By prioritizing candidate regulatory sequences (CRSs) that were both low methylation and chromatin accessible, they identify 60,000-70,000 cCREs per cell type. By H3K4me3-mediated PLAC-seq they identify 135,000-145,000 gene regulatory chromatin interactions, 21.5-26.4% of which contained cCREs. Of 15 oRG cCREs examined, 11 were confirmed by mouse transgenic assays. Using machine learning, they resolved known AD SNP rs636317, and further prioritize the SCZ variant rs4449074, again validated by mouse transgenic assay. Yet, the study lacks large-scale functional validation, cross-disorder GWAS analyses to uncover glia cell-type-specific disorder enrichments, or a systematic analysis of inter-donor variation in cell-type-specific CREs. Altogether, this manuscript presents a significant and ambitious attempt to characterize and predict glial cell type specific epigenetic and transcriptomic effects in the developing human brain but falls short of functionally dissecting glia cell type specific disease mechanisms. With revisions, this manuscript may be suitable for publication in Nature.

We are grateful to Referee #3 for the careful assessment of our manuscript and for highlighting both the strengths of the dataset and areas where the analysis and presentation could be improved. In particular, the reviewer raised important points regarding documentation of dataset reproducibility, the extent of functional validation, and the breadth of disease-related analyses. We have revised the manuscript substantially in response to these suggestions, adding new analyses and clarifying several aspects of the data and methodology to strengthen the overall study.

First, to improve transparency and reproducibility, we now summarize donor numbers, developmental stages, and replicate information directly in the main text and Extended Fig. 1. We also added detailed quality control metrics for each dataset (RNA-seq, ATAC-seq, DNA methylation, and PLAC-seq) in the Methods and expanded analyses of inter-donor variability and replicate concordance.

Second, we expanded functional validation of candidate regulatory elements. In addition to the VISTA transgenic assays reported in the original manuscript, we now provide additional spatial characterization of reporter activity supporting radial glia-associated enhancer activity. We also integrated our cCRE annotations with a published massively parallel reporter assay dataset from mid-gestational cortical cells, demonstrating enhancer activity for thousands of elements identified in our study. Finally, we added targeted functional validation of the transcription factor LHX2 using shRNA knockdown and single-cell RNA sequencing, which reveals transcriptional and cellular changes consistent with the predicted regulatory role of LHX2 in outer radial glia.

Third, we expanded the analyses of genetic and disease relevance. In addition to the Alzheimer's disease and schizophrenia examples presented in the original manuscript, we now extend our analyses to rare noncoding variants from autism cohorts. We also broadened the linkage disequilibrium score regression (LDSC) analyses to evaluate enrichment of disease-associated variants across multiple neural and glial cell types and provide a more systematic summary of predicted variant effects.

Together, these revisions improve the transparency, functional interpretation, and disease relevance of the dataset and strengthen the overall conclusions of the manuscript. We thank Referee #3 for their insightful suggestions, which substantially improved the manuscript.

Major Concerns:

1. Reproducibility: The authors generated a high-resolution atlas of the 3D epigenome and transcriptome in the developing human cortex. Ideally the reader would be able to note key facts about the number of donors per dataset and data quality ranges per dataset in the main text. Although SI Table 1 transparently reveals the scope of the dataset generated, the extent of inter-donor variation is not discussed.

We appreciate Referee #3's emphasis on reproducibility. To address this, we have incorporated key information from Table 1 into Extended Figure 1 to make donor and replicate information more visible. Specifically, in the main text (lines 96-102), we now provide details on the number of donors and developmental stages for each cell type:

"We obtained vRGs and oRGs from six donors spanning gestational weeks (GW) 15-18, and MG and OPCs from nine donors spanning GW22-24, corresponding to the onset of gliogenesis. Donors were intentionally selected across different gestational age ranges to capture the peak abundance of each cell type."

a

		vRG				oRG			
Individual	Age	RNA-seq	ATAC-seq	WGBS	PLAC-seq	RNA-seq	ATAC-seq	WGBS	PLAC-seq
ARK-2021-002	GW15	R1	R1			R1	R1		
ARK-2021-007	GW18				R1				R1
ARK-2021-008	GW18	R2	R2	R1	R2	R2	R2	R1	R2
ARK-2021-010	GW15	R3				R3			
ARK-2021-011	GW16		R3	R2	R3		R3	R2	R3
ARK-2021-013	GW18	R4	R4			R4	R4		

b

		OPC				MG			
Individual	Age	RNA-seq	ATAC-seq	WGBS	PLAC-seq	RNA-seq	ATAC-seq	WGBS	PLAC-seq
ARK-2021-001	GW22		R1				R1		
ARK-2021-003	GW23								R1
ARK-2021-004	GW24				R1				R1
ARK-2021-005	GW23	R1			R2	R1			R1
ARK-2021-006	GW22							R1	
ARK-2021-009	GW22	R2		R1		R2		R2	
ARK-2021-012	GW22		R2	R2	R3		R2		R2
ARK-2021-014	GW23	R3	R3			R3	R3	R3	
ARK-2021-016	GW23								R3

Ext. Fig. 1: a, b) Table displaying the number of replicates across each cell type for each assay. Replicate one (R1), replicate two (R2), replicate three (R3), and replicate four (R4).

a. The main text does not discuss how many donors were included in the study. From sample metadata in SI Table 1, in total 15 donors were included, but only four for vRG/oRG and three for OPC/MG RNAseq/ATACseq; there are no donors with matched vRG/oRG/OPC/MG data. Likewise, methylation data derived from two donors per cell type and PLAC-seq from three. Is it possible to include at least one set of donor-matched analyses?

We thank Referee #3 for carefully reviewing our dataset. We intentionally chose donors across different gestational age groups for vRG/oRG (GW15-18) versus OPC/MG (GW22-24) because these cell types reach peak abundance at different developmental stages. Within each age group, we prioritized obtaining both relevant cell types (vRG/oRG or OPC/MG) from the same donor whenever possible, to enable fair comparisons and minimize donor-specific effects.

However, due to limited sample availability and the rarity of certain cell types, such as MG, some datasets required the inclusion of samples from different donors. Consequently, complete donor-matched datasets across all four cell types (vRG, oRG, OPC, MG) were not feasible, our focus is to identify shared cCREs among donors.

b. Please provide basic QC information per dataset (e.g., the range of postmortem interval, RNA RIN, RNAseq percent mapping (89.58-92.75), ATACseq fraction of reads in peaks (0.32-0.53), etc) in methods.

For RNA, we provide DV200 values (an improved QC metric over RIN for FFPE samples; line 826) and TIN scores to evaluate transcript integrity after sequencing (line 836). For ATAC-seq, we now report transcription start site (TSS) enrichment and fraction of reads in peaks (FRiP) at line 865. All reported QC metrics are well above ENCODE-recommended guidelines, confirming high-quality datasets. These additions ensure that readers can assess the quality of each dataset.

c. The authors demonstrate that their vRGs and oRGs, or OPCs and MGs, are distinct from each other (Fig. 1d). It would be helpful if they included key markers to confirm that the cells are not neurons (e.g., NeuN, MAP2) or astrocytes (e.g., S100b). Better yet, are the cell type signatures similar to those in other published datasets.

We agree that it is important to confirm that our vRGs and oRGs, as well as OPCs and MGs, are distinct from neurons and astrocytes. To this end, we had already performed a CIBERSORTx analysis to estimate the abundance of different cell types within our bulk datasets using matched scRNA-seq profiles (Ext. Fig. 1e). In addition, we have now added a supplementary figure displaying RPKM expression of key marker genes, including neuronal markers (e.g., NeuN, MAP2) and astrocyte markers (e.g., S100B), to explicitly demonstrate that these cell populations do not express markers of neurons or astrocytes.

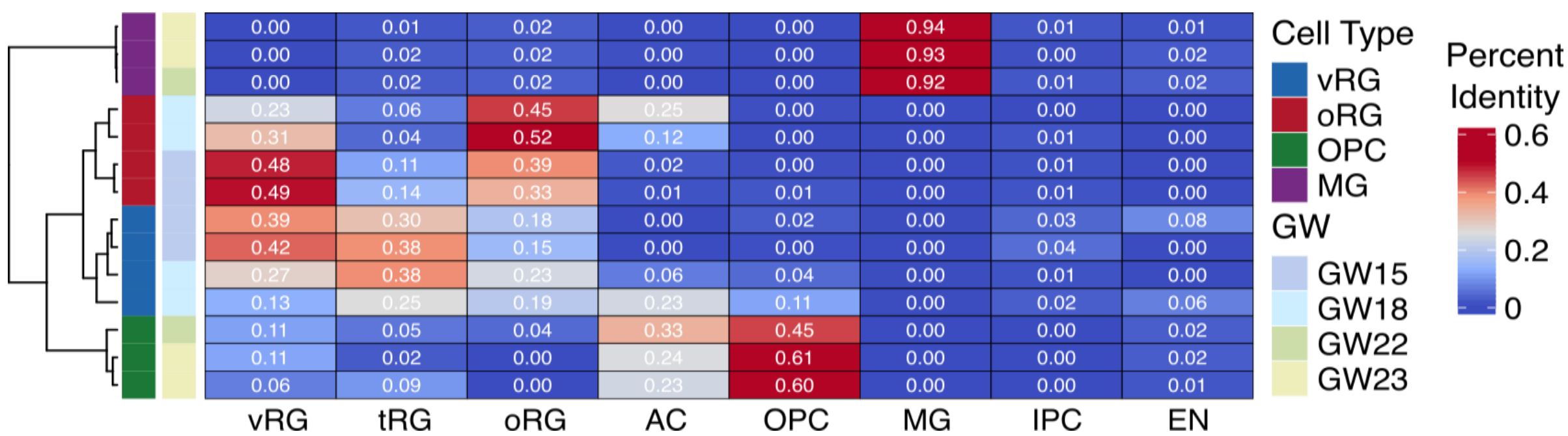

Ext. Fig. 1: e) Heatmap of cell distribution for bulk RNA-seq libraries imputed from scRNA-seq with CIBnERSORTx.

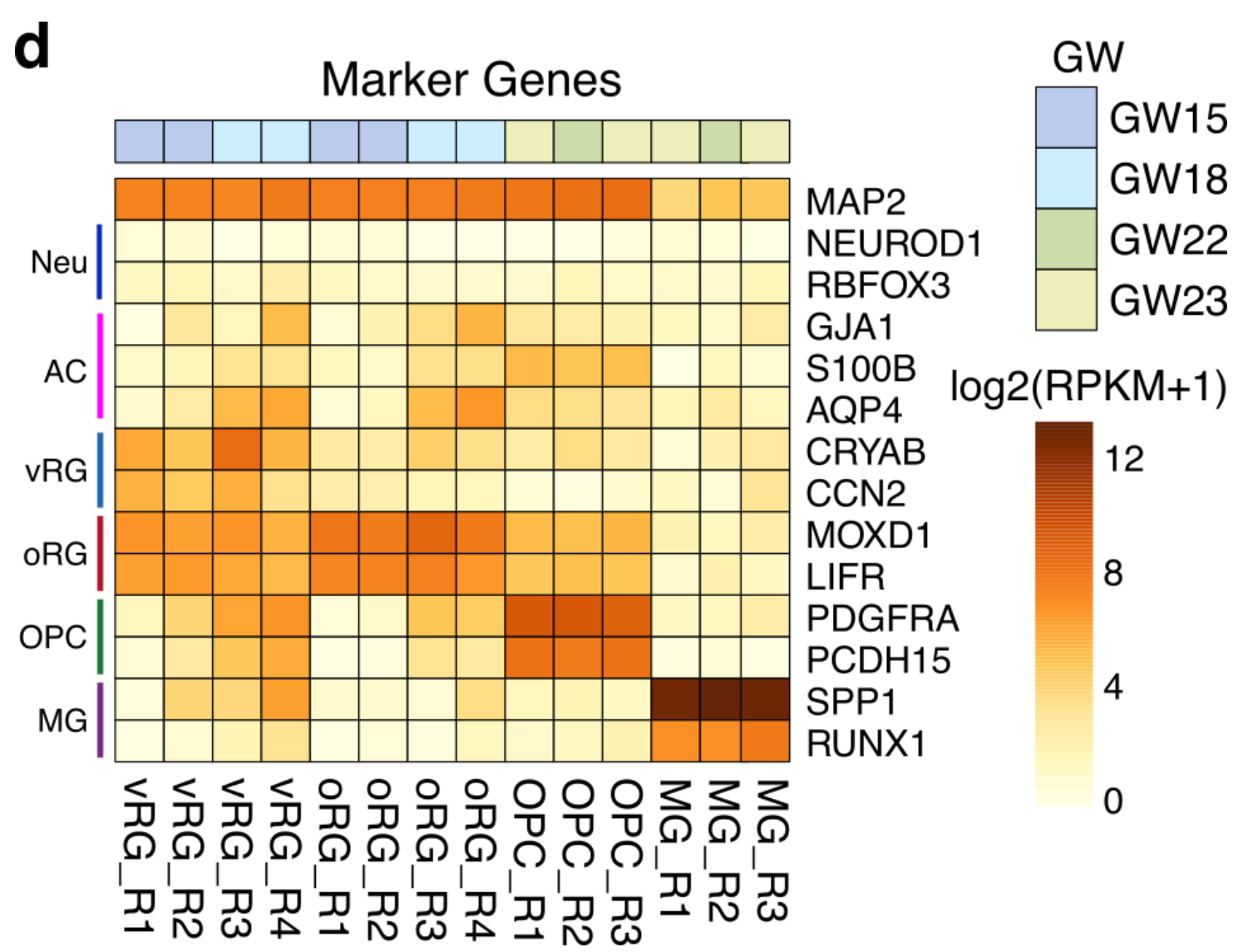

Ext. Fig. 1: d) Heatmap of RNA-seq log2(RPKM) values for key marker genes for each replicate. Astrocyte (AC), Neuronal (Neu).

d. It would be helpful to resolve the variability between RNAseq, ATACseq, DNA methylation, and PLAC-seq between donors, batches, and any other plausible sources of variation. It's unclear the extent to which peaks were reproducible across donors, or the extent to which one sample may have biased calls. What is the inter-donor correlation?

We thank Referee #3 for highlighting the importance of quantifying variability across donors, batches, and assays. To address this, we performed extensive clustering of replicates for RNA-seq, ATAC-seq, DNA

methylation, and PLAC-seq, as shown in Extended Figures 1 and 2. These analyses demonstrate that while some inter-donor variation exists, cell type-specific signals are robust, enabling reliable identification of biological features.

We observed that clustering between vRG and oRG is occasionally influenced by donor identity, likely reflecting subtle differences in gestational age. To account for this, we include donor identity as a covariate in our design matrix when identifying differential elements between vRG and oRG as stated in line 839 of the manuscript. This approach ensures that detected differences reflect consistent, cell type-specific signals rather than donor-specific effects.

a

Individual	Age	vRG RNA-seq	vRG ATAC-seq	vRG WGBS	vRG PLAC-seq	oRG RNA-seq	oRG ATAC-seq	oRG WGBS	oRG PLAC-seq
ARK-2021-002	GW15	R1	R1			R1	R1		
ARK-2021-007	GW18				R1				R1
ARK-2021-008	GW18	R2	R2	R1	R2	R2	R2	R1	R2
ARK-2021-010	GW15	R3				R3			
ARK-2021-011	GW16		R3	R2	R3		R3	R2	R3
ARK-2021-013	GW18	R4	R4			R4	R4		

b

Individual	Age	OPC RNA-seq	OPC ATAC-seq	OPC WGBS	OPC PLAC-seq	MG RNA-seq	MG ATAC-seq	MG WGBS	MG PLAC-seq
ARK-2021-001	GW22		R1				R1		
ARK-2021-003	GW23								R1
ARK-2021-004	GW24				R1				R1
ARK-2021-005	GW23	R1			R2	R1			R1
ARK-2021-006	GW22							R1	
ARK-2021-009	GW22	R2		R1		R2		R2	
ARK-2021-012	GW22		R2	R2	R3		R2		R2
ARK-2021-014	GW23	R3	R3			R3	R3	R3	
ARK-2021-016	GW23								R3

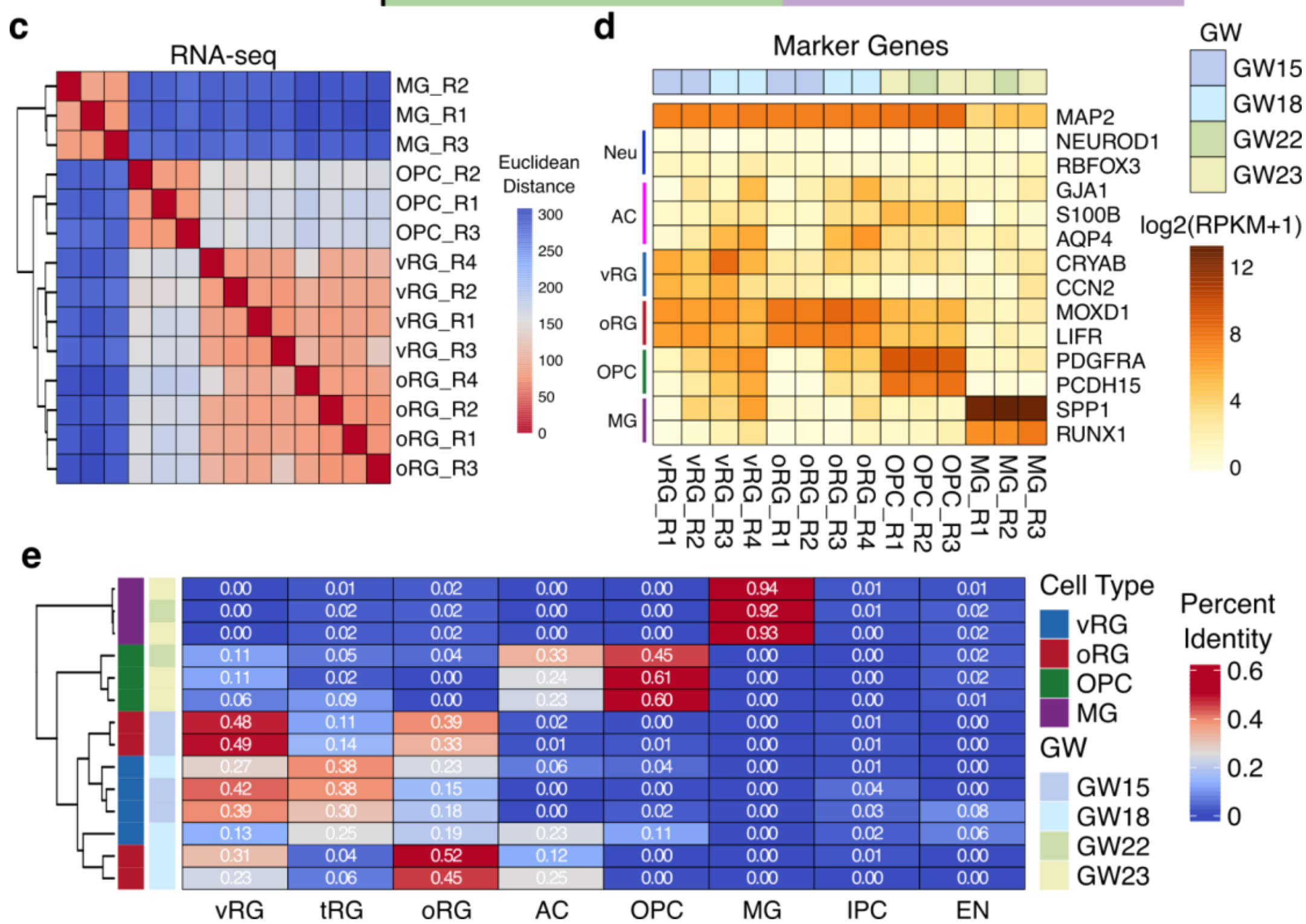

Ext. Fig. 1: a, b) Table displaying the number of replicates across each cell type for each assay. Replicate one (R1), replicate two (R2), replicate three (R3). c) Hierarchical clustering of Euclidean distance for RNA-seq libraries performed post batch correction with DESeq2. d) Heatmap of log2(RPKM) values for key marker genes for each replicate. e) Heatmap of cell type composition estimated for bulk RNA-seq libraries using scRNA-seq as reference with CIBERSORTx.

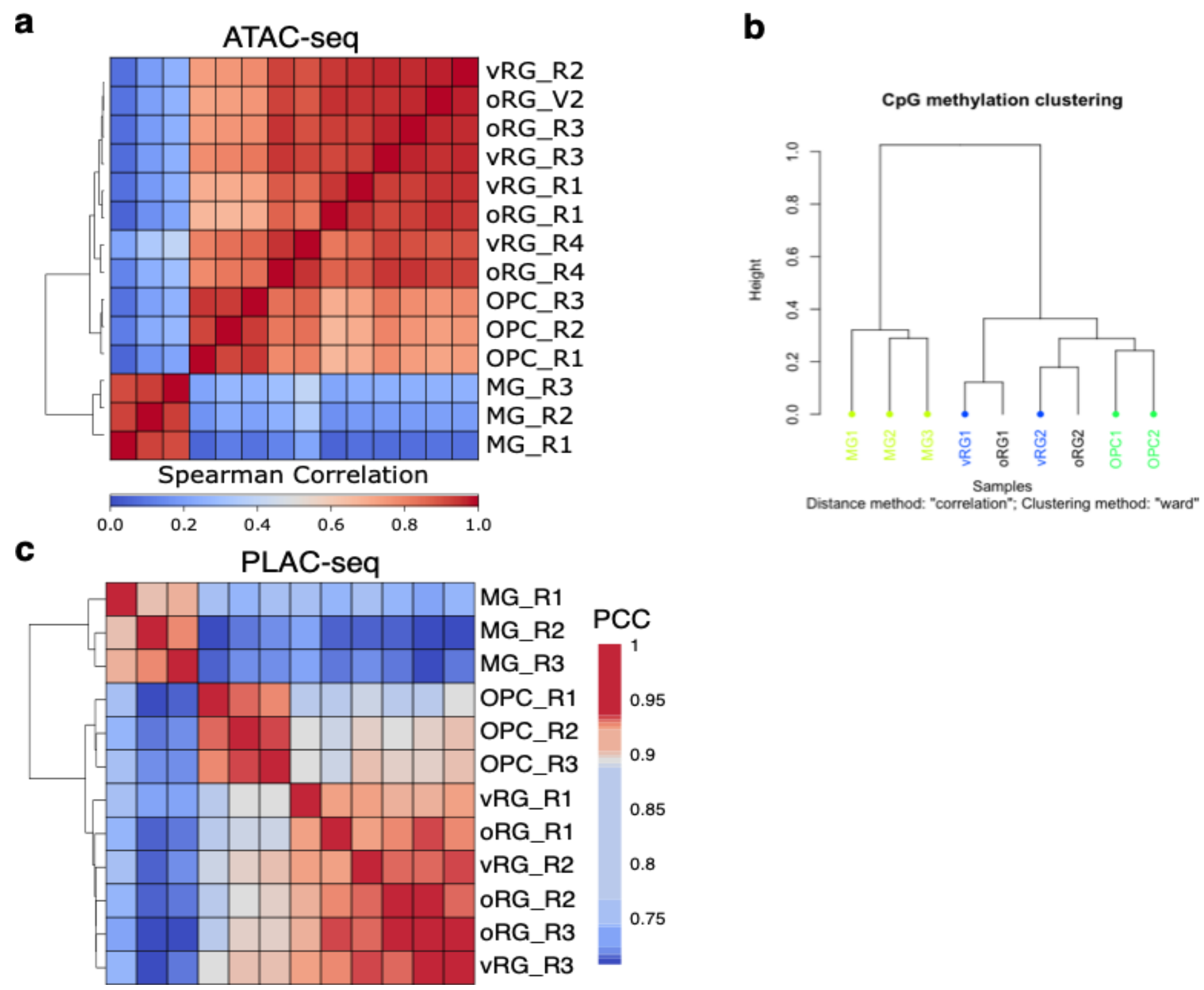

Ext. Fig. 2: a) Heatmap of Spearman correlation of ATAC-seq libraries within unified peaks across all four cell types. b) Clustering of CpG methylation. c) Heatmap of Pearson correlation of PLAC-seq libraries obtained with HPRep.

e. Please summarize the number of peaks, chromatin interactions, CREs called by cell type per donor. Are there outlier CREs/DARs/chromatin loops not detectable across replicated that should be removed?

We appreciate the concern about identifying outliers in the data. The number of peaks called per replicate has now been added to Table 1_3. Across all replicates, we identified 65,210, 69,172, 80,177, and 78,776 peaks in vRG, oRG, OPC, and MG, respectively. When assessing reproducibility, 110,067 (vRG), 107,708 (oRG), 105,866 (OPC), and 101,087 (MG) peaks were detected in at least two biological replicates. Lastly, the merged peaks account for 91.6-98.7% of the peaks in individual samples, indicating that the majority of peaks are reproducible across donors.

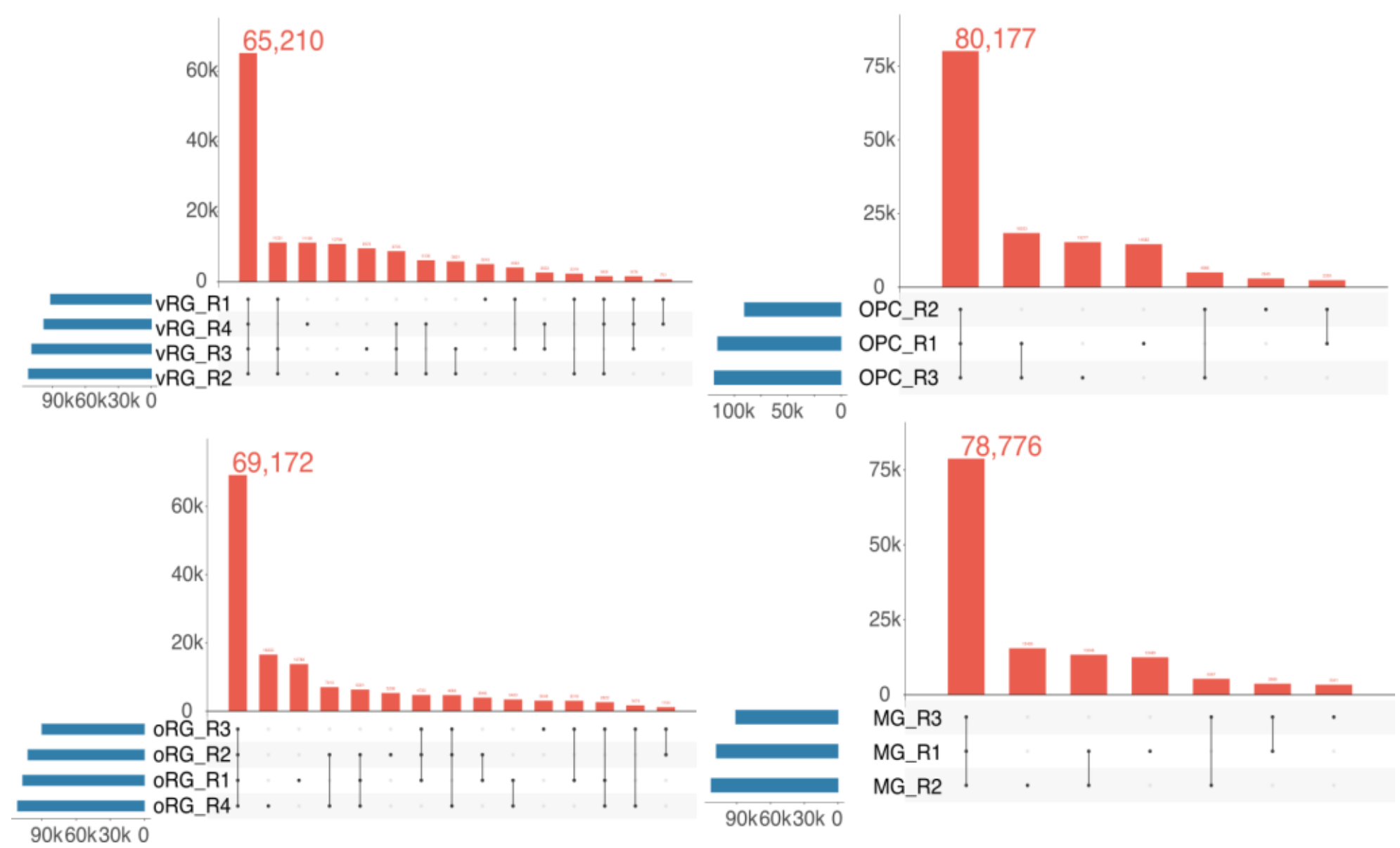

Upset plots displaying the overlap of replicates within vRG, oRG, OPC, and MG.

The number of chromatin interactions per replicate is provided in Table 1_5. Final interaction calls were generated after downsampling pooled replicates to approximately 60 million usable reads (range: 59,777,372-

61,171,167) within universal H3K4me3 anchors. We observed a strong correlation between the number of usable reads and the total number of significant interactions (Pearson correlation, R = 0.899), as shown in the figure below, supporting the robustness of interaction detection.

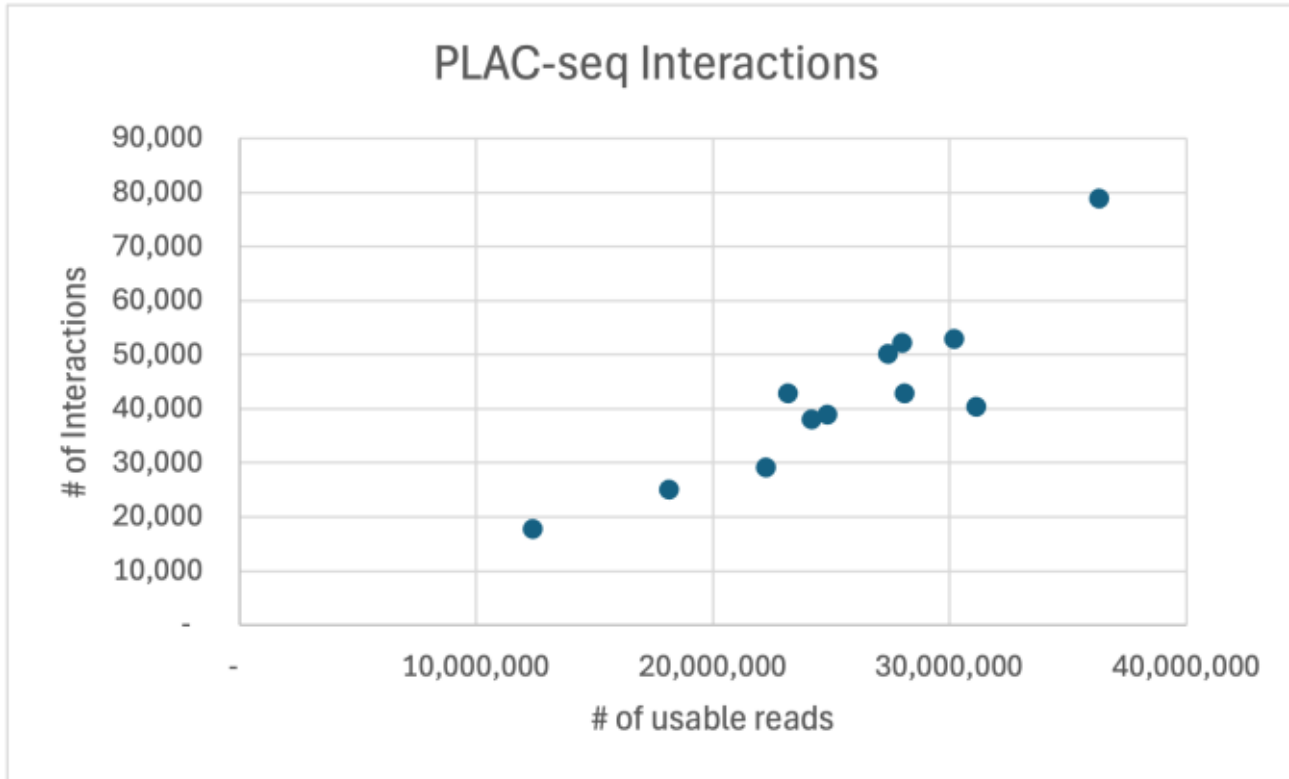

Scatter plot comparing the number of usable PLAC-seq reads with the number of significant interactions.

To further evaluate reproducibility and identify potential outlier replicates, each replicate was independently downsampled to 10 million usable reads and analyzed using HPRep (PMID 34563051). Within each cell type, Pearson correlations of interaction strengths exceeded 0.9 across replicates, and no replicate showed systematic deviation in the number of interactions or in correlation metrics (Ext. Fig. 2c). Based on these analyses, no chromatin loop outliers were identified that warranted removal.

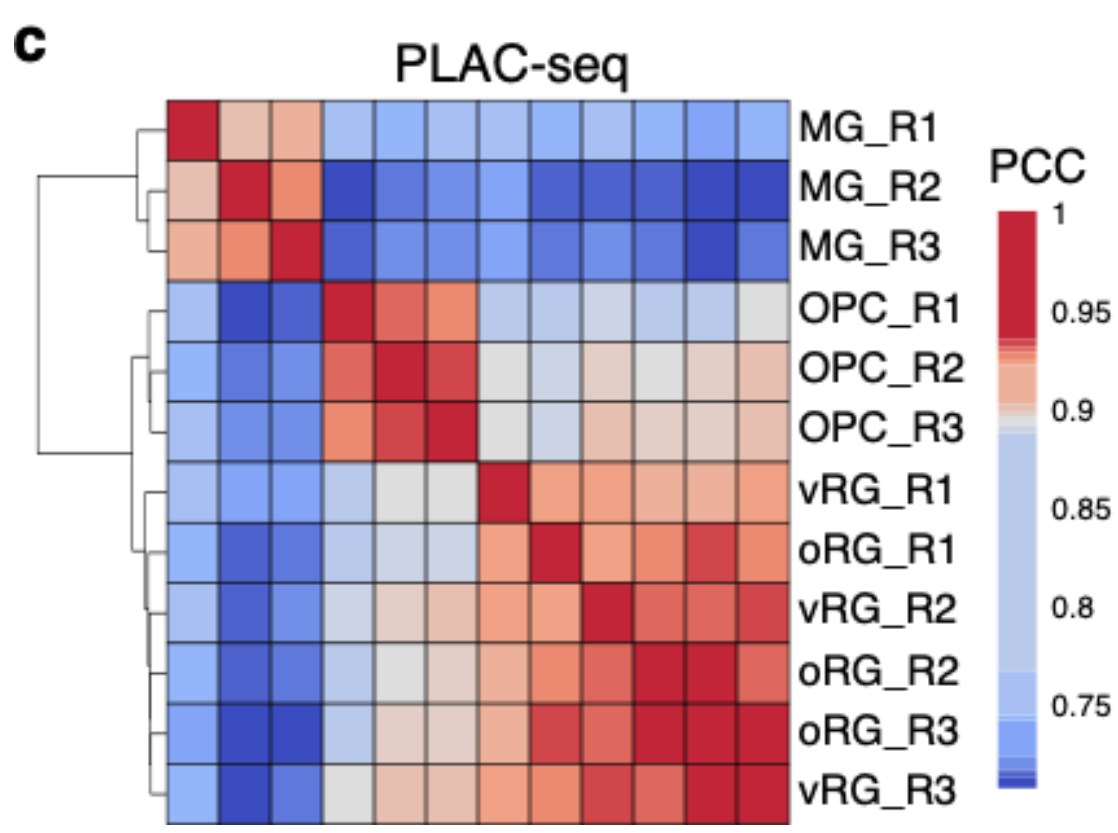

Ext. Fig. 2: c) Heatmap of Pearson correlation of PLAC-seq libraries obtained with HPRep.

We appreciate the suggestion. Our current cCRE definition integrates both ATAC-seq and methylation data. However, we do not have both assays performed on the same donors, which makes reporting cCRE counts per donor infeasible. To ensure robust CRE identification, we instead focused on high-quality, reproducible cCREs by assessing consistency across biological replicates. This approach captures the most reliable regulatory elements while accounting for biological variability. Specifically, we evaluated Spearman correlations of ATAC-seq read counts within peaks, Ward-based correlations of CpG methylation levels for WGBS, and Pearson Correlation for PLAC-seq interactions (Ext. Fig 2a-c). These analyses demonstrate strong agreement within each cell type and support the robustness of the final CRE, DAR, and chromatin interaction sets used in downstream analyses.

2. Functional validation: The tens of thousands of CREs and chromatin interactions identified across human neocortex vRGs, oRGs, OPCs, and MGs, as well as their links to brain disease, are minimally validated.

We appreciate Referee #3's concern regarding functional validation of our predicted CREs and have implemented multiple complementary approaches to assess their activity and cell type specificity."

a. The authors identify 60,000-70,000 CREs per cell type, but only experimentally validate 15 in the developing mouse. Moreover, the CREs examined in vivo are not explored in a cell type specific manner. Are the tested CREs active in the predicted cell types? Are they specific to the predicted cell types?

To provide complementary, orthogonal support for the functional relevance of our cCRE annotations at scale, we examined whether previously validated neural enhancers are preferentially associated with our cell type-specific regulatory interactions. Specifically, we assessed enrichment of 1,233 functional neural enhancers relative to 1,868 negative elements annotated in the VISTA Enhancer Browser among promoter-interacting cCREs identified in our study, compared with distance-matched controls. We found that validated neural enhancers are significantly enriched among interacting cCREs in vRG, oRG, and OPC, but not in MG, as expected, given that the assays were performed in earlier developmental mouse brain stages, when MG-specific enhancer activity is limited or not yet established.

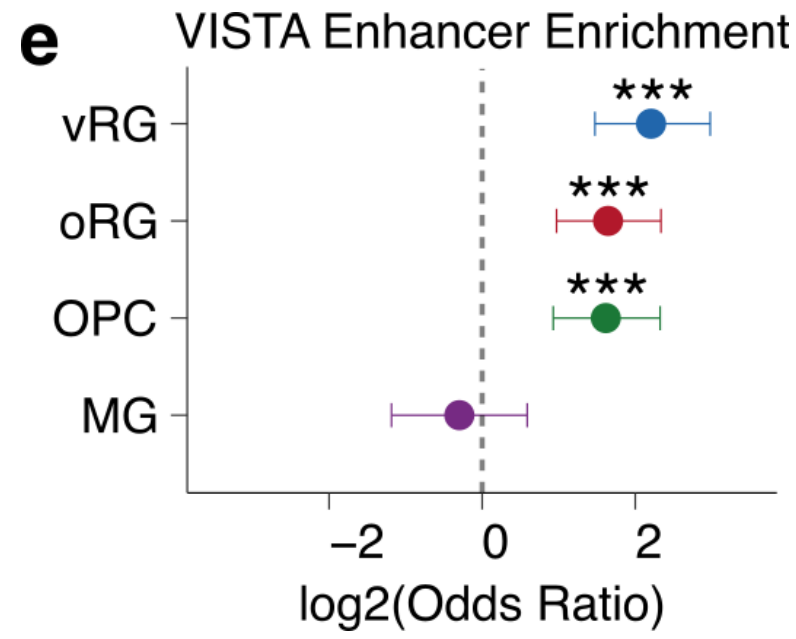

Ext. Fig. 5: e) Forest plot showing the association of cCREs with positive neuronal VISTA elements or negative neuronal VISTA elements. Data are shown as odds ratios with 95% confidence intervals (Fisher's exact test, *: P< 0.05, **: P < 0.01, ***: P < 0.001).

To address the cell type specificity of our in vivo enhancer assays, we performed additional sectioning and high-resolution microscopy on embryos used to validate RG cCREs. These analyses revealed that the lacZ reporter activity is predominantly localized to the ventricular zone, where RG cell bodies reside, for all four elements (Ext. Fig. 5c) we tested (hs3126, hs3127, hs3129, hs3148). This spatial restriction is consistent with activity in RG populations and supports the predicted RG association of these cCREs.

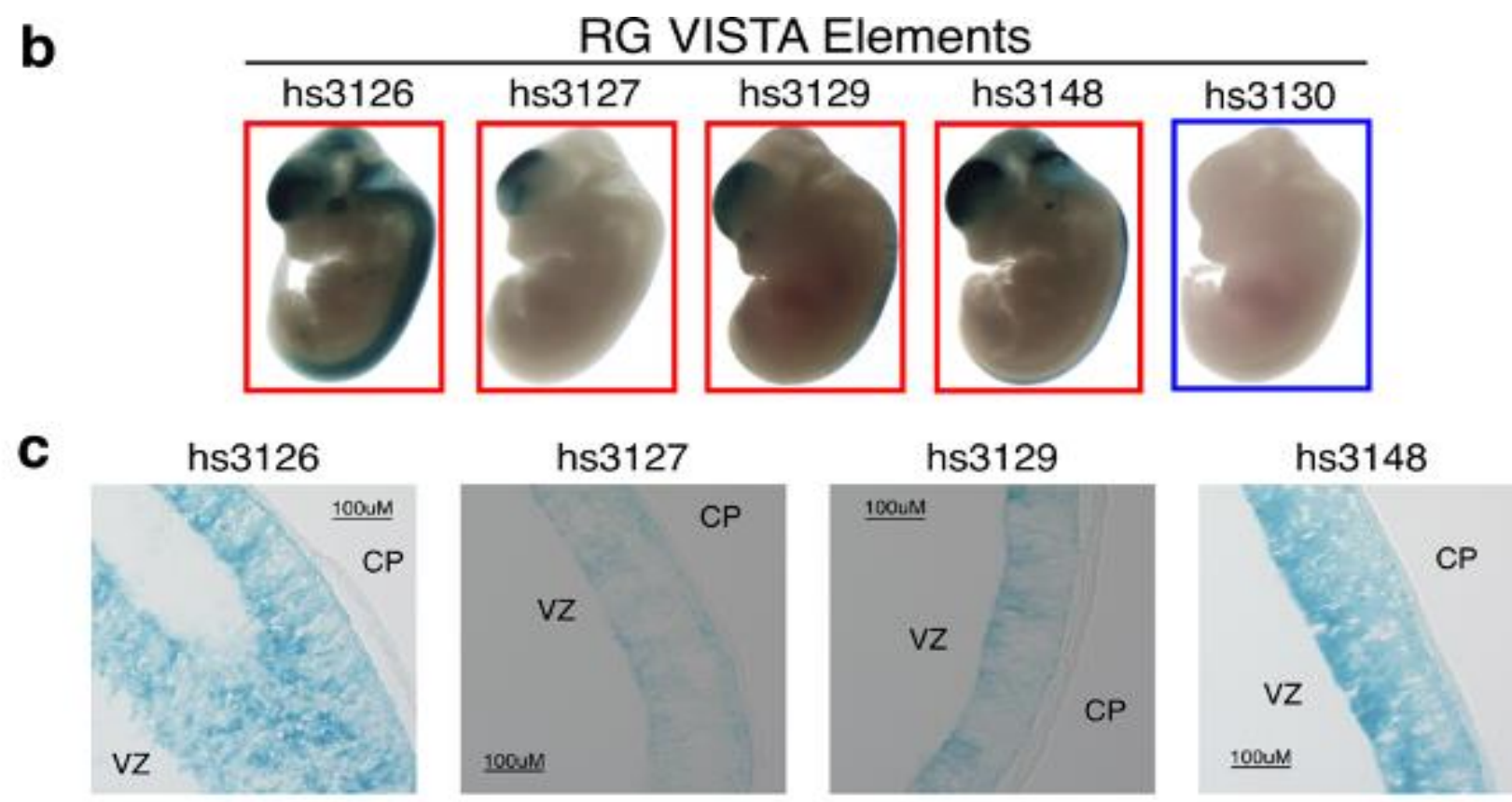

Ext. Fig. 5: b) VISTA results for select RG peaks at e12.5. c) Brain sections of RG VISTA elements. (VZ) ventricular zone. (CP) cortical plate

For two elements (hs3126 and hs3127), we additionally observed reporter signals near the cortical plate. This suggests that while these cCREs are active in RGs, they may also exhibit activity in downstream neural lineages or broader developmental contexts.

We emphasize that the in vivo transgenic mouse assays were designed as proof-of-principle functional validation to demonstrate regulatory activity of predicted human cCREs in vivo, rather than as a comprehensive or exclusive assessment of cell type specificity. Given the technical constraints of transgenic assays and species differences between human and mouse cortical development, systematic functional testing of tens of thousands of CREs in a fully cell type-resolved manner is not currently feasible.

b. Given the scope of CREs identified, it would be substantially more informative to test them using massively parallel reporter assays. Ideally, validation would be done in a human specific and cell type specific context, either in primary human and/or human induced pluripotent stem cell derived populations, but doing so in transformed glial cell lines would likely be informative as well.

We thank Referee #3 for this suggestion. An MPRA requires substantial resources, effort, and time, and warrants a separate study. Fortunately, our colleagues have published an MPRA study in mid-gestation human cortical cells and cerebral organoids (PMID 38781390), and we performed cross-validation of our annotated cCREs using these published datasets. A total of 9,032, 9,027, 8,587, and 7,910 cCREs from vRG, oRG, OPC, and MG, respectively, were tested, representing 12.4-13.1% of cCREs. Across all four cell types in our study, the fraction of cCREs exhibiting enhancer activity in the MPRA, 44.5%-51.1%, was comparable to that reported in the original study, which selected elements either overlapping H3K27ac signal or chromatin interactions (Ext. Fig. 3f). These results support the functional potential of a subset of our predicted regulatory elements.

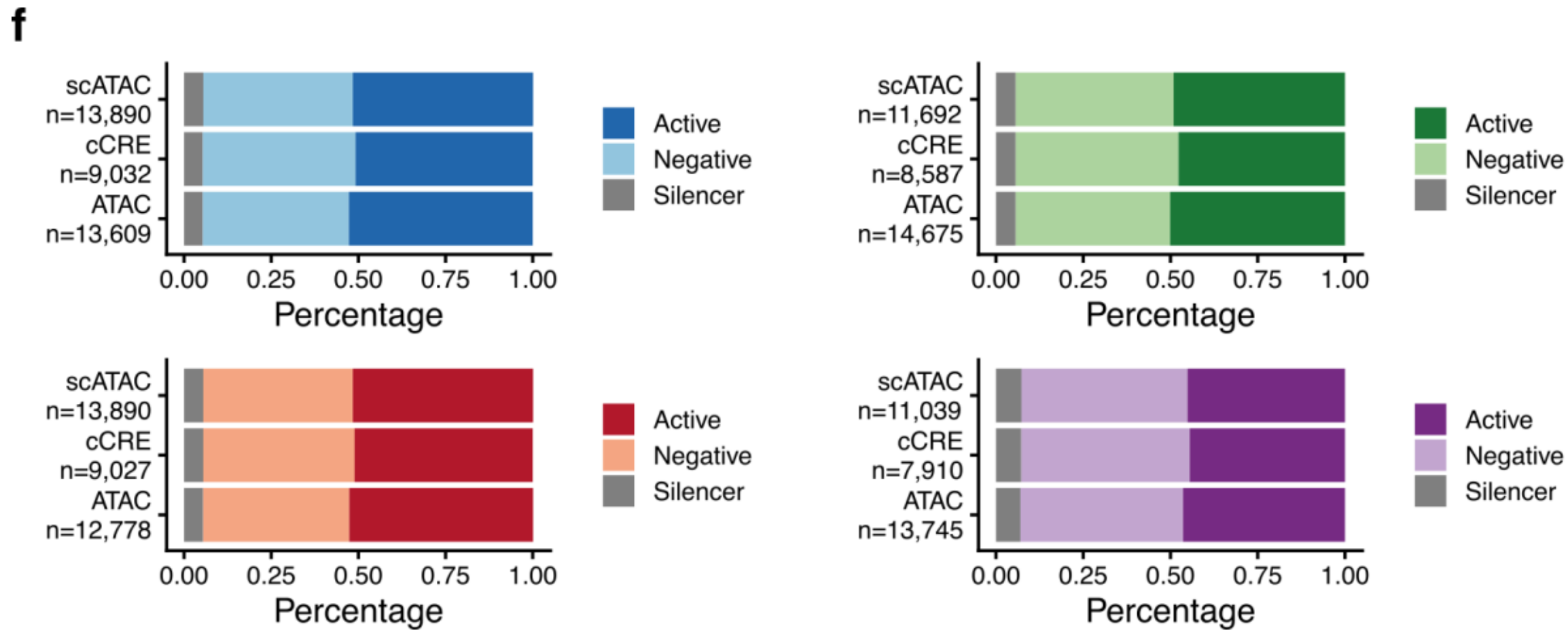

Ext. Fig. 3: f) Barplot of MPRA in mid-gestation human cortical cells that overlap cell type-specific peaks identified by scATAC (PMID 38781390), cCREs, and ATAC-seq peaks. For scATAC-seq, microglia, radial glia, and astrocyte/oligodendrocyte accessible peaks were used.

c. Of 15 oRG cCREs examined, 11 were confirmed by mouse transgenic assays. Yet, the rationale for selecting these 15 oRG CREs is unclear. Moreover, the in vivo transgenic assay is not described in the methods and there is no detailed description in the figure legend of the results presented Figure 2F/4C.

Candidate elements for VISTA mouse transgenic assays were initially selected from ATAC-seq peaks identified in RG, IPC, eN, and iN (PMID: 33057195). First, ATAC-seq reads were merged into a peak set and subsequently quantile-normalized. Second, peaks overlapping transcription start sites defined by CAGE-seq were removed, and remaining regions were required to participate in a 3D chromatin interaction and overlap the top 15k accessible orthogonal regions in mouse embryonic brain at E11.5 (PMID: 32728240; PMID: 29434377). Lastly, peaks were further filtered for strong ATAC-seq signal (> 0.8 CPM) and cell type specificity enrichment (> 0.5 CPM difference), yielding 61 candidate regions. From these, 29 elements were manually selected for testing. Specifically, manual selection would discard 1) the peaks that overlap existing Vista elements, 2) broad/low peaks that we suspected could have been called erroneously by the peak caller (despite overall high signal value), 3) peaks likely hard to synthesize/clone due to repeats. That resulted in 42 elements designed/ordered, many of which failed at cloning, resulting in 29 curated elements, which largely overlapped with cCREs annotations (accessible and lowly methylated, 20/29) or were chromatin interacting regions (20/29, of which 16 are cCREs) identified in one of the vRG, oRG, OPC, and MG datasets generated in this study (Table 4). We have now clarified this rationale in the method section (line 959).

We agree that the in vivo transgenic assay was insufficiently described in the original manuscript. We have now added a reference at line 975 to the detailed protocol stating that transgenic mouse embryos were generated as described previously (PMID 34913122) with the exception that mouse embryos were collected at E12.5.

3. Disease relevance: The authors apply a machine learning model to explore whether GWAS SNPs linked to Alzheimer’s disease and schizophrenia credibly impact chromatin accessibility.

a. Of the 565 of 6,350 credible AD variants that were accessible in at least one cell type, among which 68 were predicted to perturb accessibility, the authors highlight only one example (rs636317). Of the 929 of 11,360 SCZ variants that were accessible, 112 were predicted to perturb accessible. Rather than highlight one SNP, be it rs636317 or rs4449074, it would be substantially more informative to compare GWAS from many brain diseases/disorders/traits and present which GWAS are most enriched for which glial cell types.

We thank Referee #3 for this constructive suggestion to broaden the disease relevance analysis and to compare enrichment of genetic risk across multiple brain-related diseases and traits in a cell type-specific manner.

In the original manuscript, we focused on Alzheimer's disease (AD) and schizophrenia (SCZ) as representative examples to illustrate how our machine learning model can prioritize candidate causal variants and predict their effects on chromatin accessibility, and we highlighted individual SNPs (e.g., rs636317 and rs4449074) as illustrative case studies. As part of this revision, we additionally performed a similar analysis for noncoding rare variants identified previously from multiple autism spectrum disorder (ASD) cohorts, focusing on those that overlap human accelerated regions (HARs), VISTA enhancers, or conserved regions predicted to function as neural enhancers (PMID 39019033) (Ext. Fig. 10f, Ext. Fig. 11e, f). We agree, however, that a broader, trait-wide comparison is substantially more informative.

To address this concern, we expanded our linkage disequilibrium score regression (LDSC) analysis to evaluate heritability enrichment across GWAS for multiple brain-related diseases and traits in a cell type-specific manner. While Fig. 4a already reports enrichment of interacting cCREs within glial cell types, we now extend this analysis to include accessible chromatin peaks from the Song et al. (2020) datasets, thereby enabling broader comparisons across neural and glial populations at this developmental stage. These results provide a more comprehensive view of which brain-related diseases and traits show preferential enrichment in specific glial cell types.

In addition, we provide a complete summary of predicted effects on chromatin accessibility across cell types in Table 8. This table enables readers to systematically assess the distribution of predicted regulatory impacts across diseases, variants, and cell types, rather than relying on individual examples.

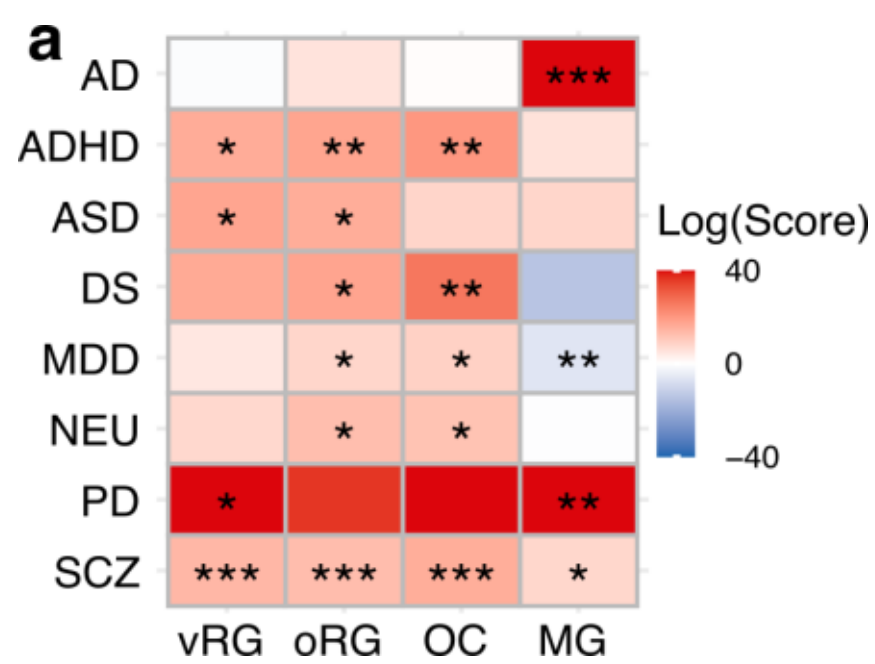

Fig. 4: a) Heatmap of LDSC score for interacting cCRE. Red, the disorder is positively enriched. Blue, the disorder is negatively enriched. (*: P < 0.05, **: P < 0.01, ***: P < 0.001).

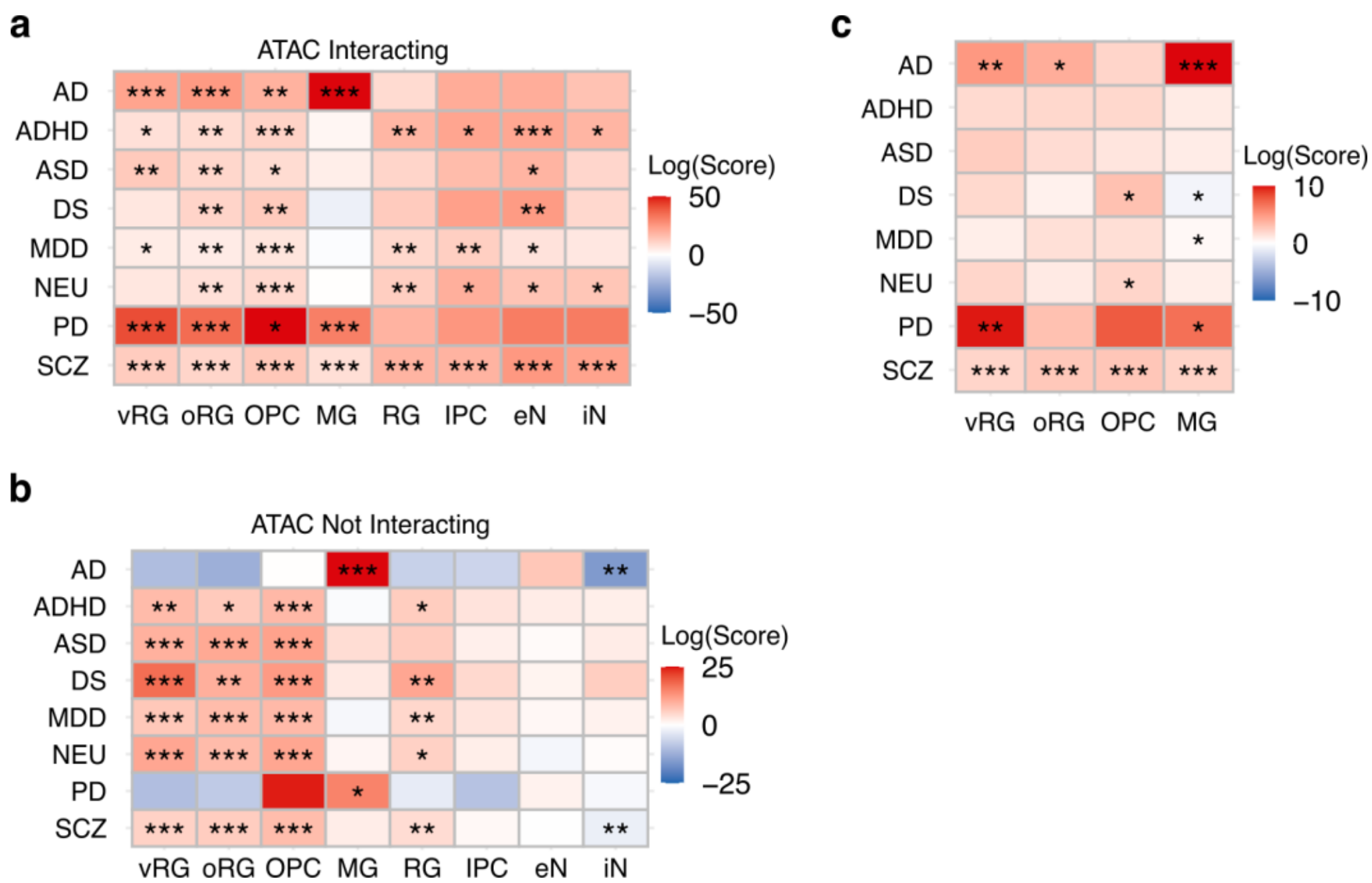

Ext. Fig. 10: Heatmap of LDSC score for a) ATAC-seq peaks involved in 3D interaction b) ATAC-seq peaks not involved in 3D interactions. Red, the disorder is positively enriched. Blue, the disorder is negatively enriched. (*: P < 0.05, **: P < 0.01, ***: P < 0.001). c) Heatmap of LDSC score for interacting cCRE. Red, the disorder is positively enriched. Blue, the disorder is negatively enriched. (*: P < 0.05, **: P < 0.01, ***: P < 0.001).

b. The authors highlight that both vRG and oRG cCREs and DARs enriched for annotated HARs. Was this likewise true for OPCs and MGs?

We appreciate Referee #3's concern about enrichment of HARs in non-RG cell types. The short answer is no. Actually, MGs are depleted for annotated HARs. In Figure 5a and Supplementary Table 9.3, we show that the Z-score compared to an empirical null distribution for OPC and MG are 0.42 and -13.48, respectively, for all cCREs and -2.5 and -10.55 for cell type-specific peaks. This demonstrates a lower-than-expected overlap between MG cCREs and HARs.

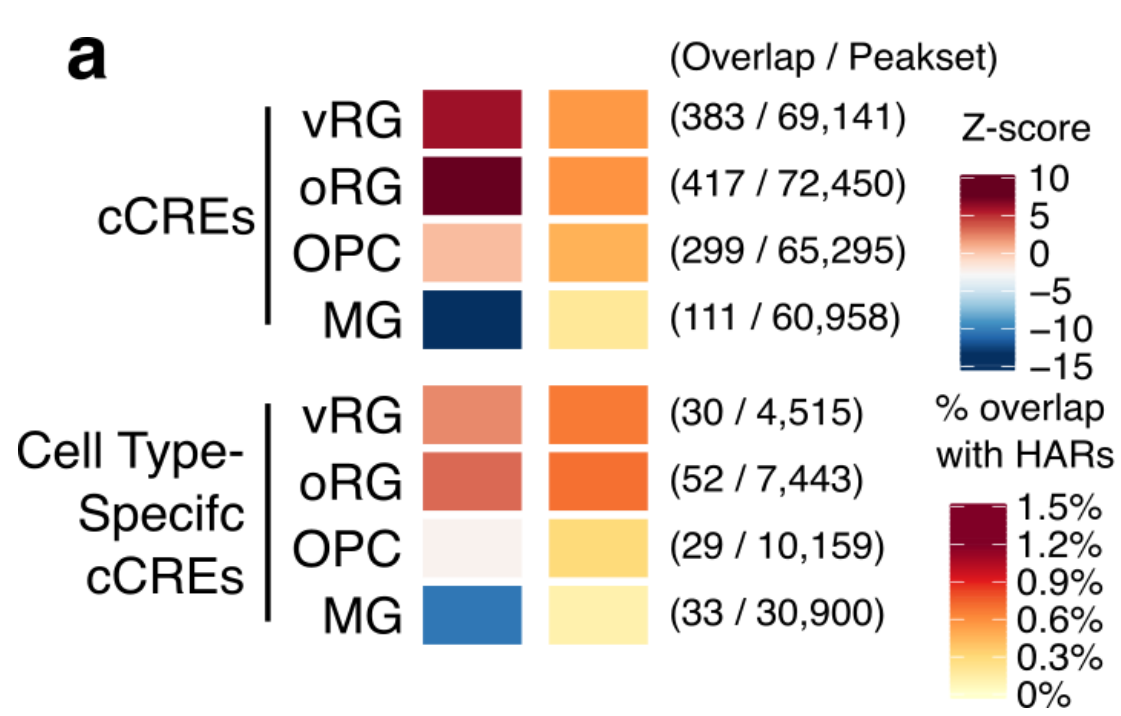

Fig. 5: a) Heatmap displaying the z-score (left) for overlap of either cCREs or cell type-specific cCREs compared with a randomly sampled empirical null distribution, as well as the percentage of HARs overlapping the data (right).

c. It's unclear why the analysis of HARs, and TF binding within HARs, was restricted to oRGs. Could this analysis be integrated into the larger cross-disorder GWAS suggested above? Identifying disease loci within TF binding sites and/or HARs?

We thank Referee #3 for this insightful suggestion. Our initial focus on oRGs was motivated by the observation that oRG cCREs show significantly stronger enrichment for annotated HARs compared to vRGs, OPCs, and microglia (MGs). This enrichment was substantially higher than in other cell types, motivating a more detailed characterization of HAR-associated regulatory logic in oRGs.

We agree, however, that a subset of HARs may also be functionally relevant in other cell types, even in the absence of global enrichment. To address this, we have now expanded our analysis to include cell type-specific model predictions for HARs in vRGs, OPCs, and MGs, which are provided in Supplementary Table

9_6. This addition allows readers to assess HAR-associated regulatory activity across all glial populations examined, rather than only oRGs.

We also agree that TF binding is highly relevant for prioritizing disease-associated variants and HARs. Building on prior work (PMID 33106633), we report, in Tables 9 and 10, prominence scores and corresponding P values for each prediction, which quantify whether the sequence surrounding a variant exhibits higher model signal than shuffled controls. Importantly, we extend this framework to also report prominence scores for negative (repressive) contributions, enabling identification of both activating and repressive regulatory motifs. For example, HARsv2_2157_2 (Table 9_6) is predicted to bind the repressor ZEB1 and does not show significant positive prominence (P = 0.199) but exhibits significant negative prominence (P = 0.013), consistent with a repressive regulatory role.

Together, these additions integrate HAR annotation, TF binding predictions, and cell type-specific regulatory modeling to support systematic prioritization of disease-associated loci across multiple cell types. While motifBreakR predictions are based on sequence alone, prominence scores provide cell type-specific functional predictions, enabling a more nuanced interpretation of disease-relevant regulatory mechanisms.

d. Again, functional validation of these highlighted CREs by MPRA or CRISPRprime and/or functional validation of the cell-type-specific role of these TFs would dramatically inform the relevance of the analyses presented.

We thank Referee #3 for their constructive suggestion. As previously described, we integrated our cCRE annotations with a massively parallel reporter assay (MPRA) conducted in mid-gestation human cortical cells and cerebral organoids (PMID 38781390), providing large-scale functional support for predicted regulatory activity.

Previously, we found that LHX2 TFBSs were enriched in oRG compared with vRG cCREs (Fig 3b) and that oRG-specific TFBSs interacted with 16 genes differentially expressed in oRGs (Ext. Fig. 7c). We therefore performed targeted functional validation of LHX2 to assess its cell type-specific role, as described at line 228.

To assess the role of LHX2 in oRG, we performed short hairpin RNA (shRNA)-mediated knockdown followed by single-cell RNA sequencing (scRNA-seq) to examine its impact on transcription and neuronal differentiation. UMAP clustering identified eight distinct cell types, with minimal batch effects (Ext. Fig. 8a-c). Successful knockdown of LHX2 was confirmed within the oRG cluster, with shLHX2_2 producing a more pronounced reduction than shLHX2_1 (Fig. 3c). To identify cell types that exhibited transcriptomic dysregulation upon LHX2 knock down, we applied scDist (PMID 39218971), a statistically rigorous method to fairly rank transcriptomic changes based on the Euclidean distance. Both shRNAs produced concordant directional transcriptomic changes, but the effect was substantially greater with shLHX2_2, consistent with its stronger knockdown efficiency (Fig. 3d). Subsequent analyses therefore focused on shLHX2_2. The oRG and astrocytes (AC) clusters were the most perturbed, with the AC marker SPARCL1 being of high feature importance (Ext. Fig. 9a), in agreement with prior work suggesting that LHX2 is required to repress astrogenesis (PMID 21690374). In addition to the increase of SPARCL1, we observed a general upregulation of the 16 DEGs predicted as targets of LHX2 TFBS, including FAT3, SPRY1, PRKCA, and PREX2 (Fig 3e, Ext. Fig. 9a, c). These results support a model in which LHX2 regulates AC-associated genes and downstream differentiation trajectories. Furthermore, the relative abundance of oRG cells increased significantly following shLHX2_2 knockdown at the expense of differentiated neurons (Fig. 3f. Ext. Fig. 9b). Despite this relative expansion, shLHX2_2 exhibited a higher proportion of cells in G1 phase (Fig. 3g; Ext. Fig. 9e), indicating reduced proliferative activity and the onset of a transition toward differentiation or quiescence. Taken together, loss of LHX2 reduces self-renewal and shifts oRG towards a more differentiated, AC-like state. Consistent with a weaker perturbation, shLHX2_1 showed a similar transcriptomic trend but did not produce comparable compositional or cell-cycle changes, likely due to incomplete depletion of LHX2 (Ext. Fig. 9d, f-g).

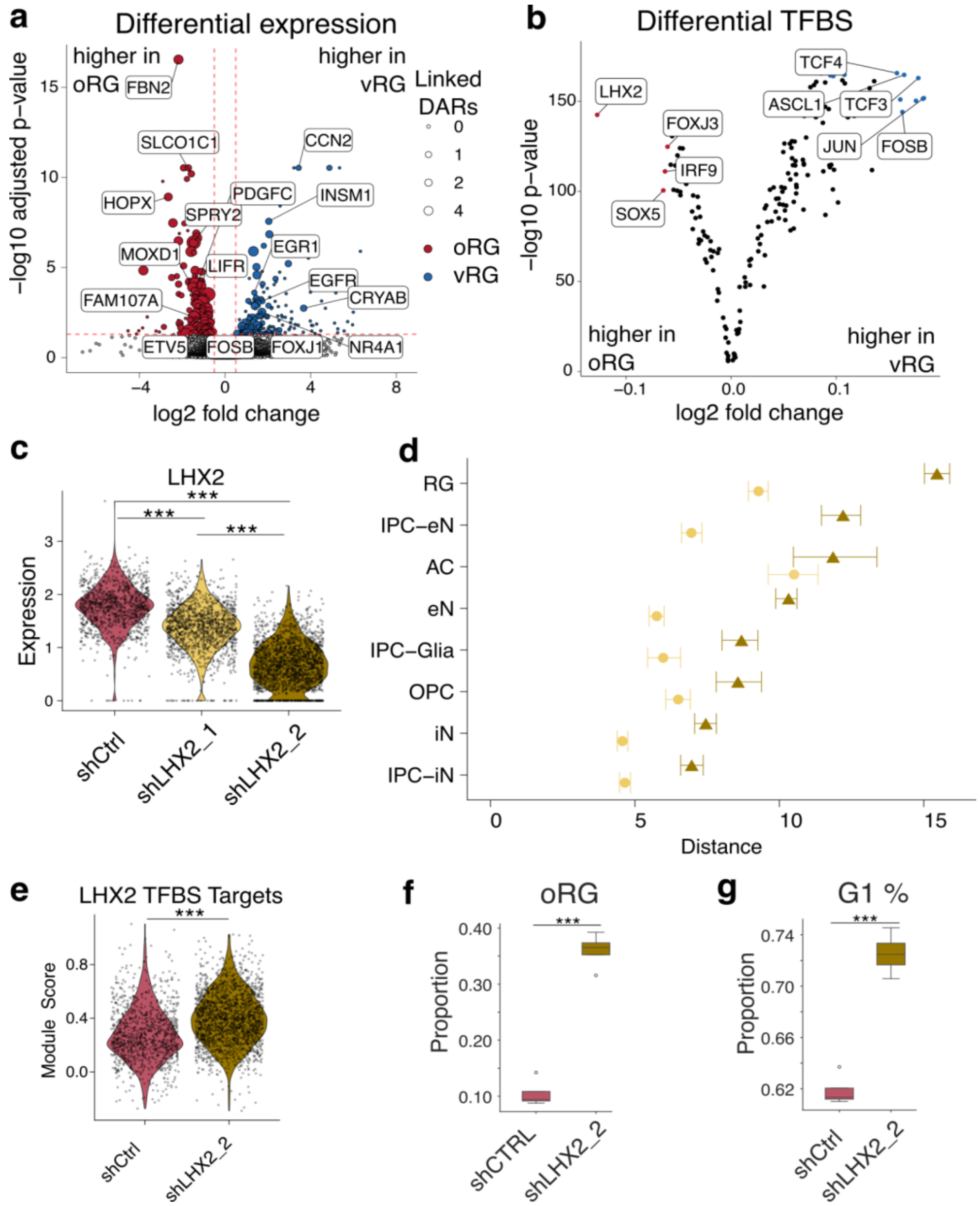

Fig. 3: a) Volcano plot of differentially expressed genes. The size of each dot is determined by the amount of DARs overlapping the TSS or interacting with each gene. b) Volcano plot of motif binding difference between vRG and oRG, using TOBIAS Footprinting Analysis. c) Violin plot of *LHX2* expression within the oRG cluster. (Wilcoxon Rank Sum test, *: P< 0.05, **: P < 0.01, ***: P < 0.001). d) Distance score obtained by scDist for each cell type. e) Violin plot of the module score of the 16 DEGs targets of the LHX2 motif within the oRG cluster. (Wilcoxon Rank Sum test, *: P< 0.05, **: P < 0.01, ***: P < 0.001). f) Boxplot of the proportion of oRG. (scCODA, *: P< 0.05, **: P < 0.01, ***: P < 0.001). g) Boxplot of the proportion of cells at the G1 state within the oRG cluster (Paired T-test, *: P< 0.05, **: P < 0.01, ***: P < 0.001).

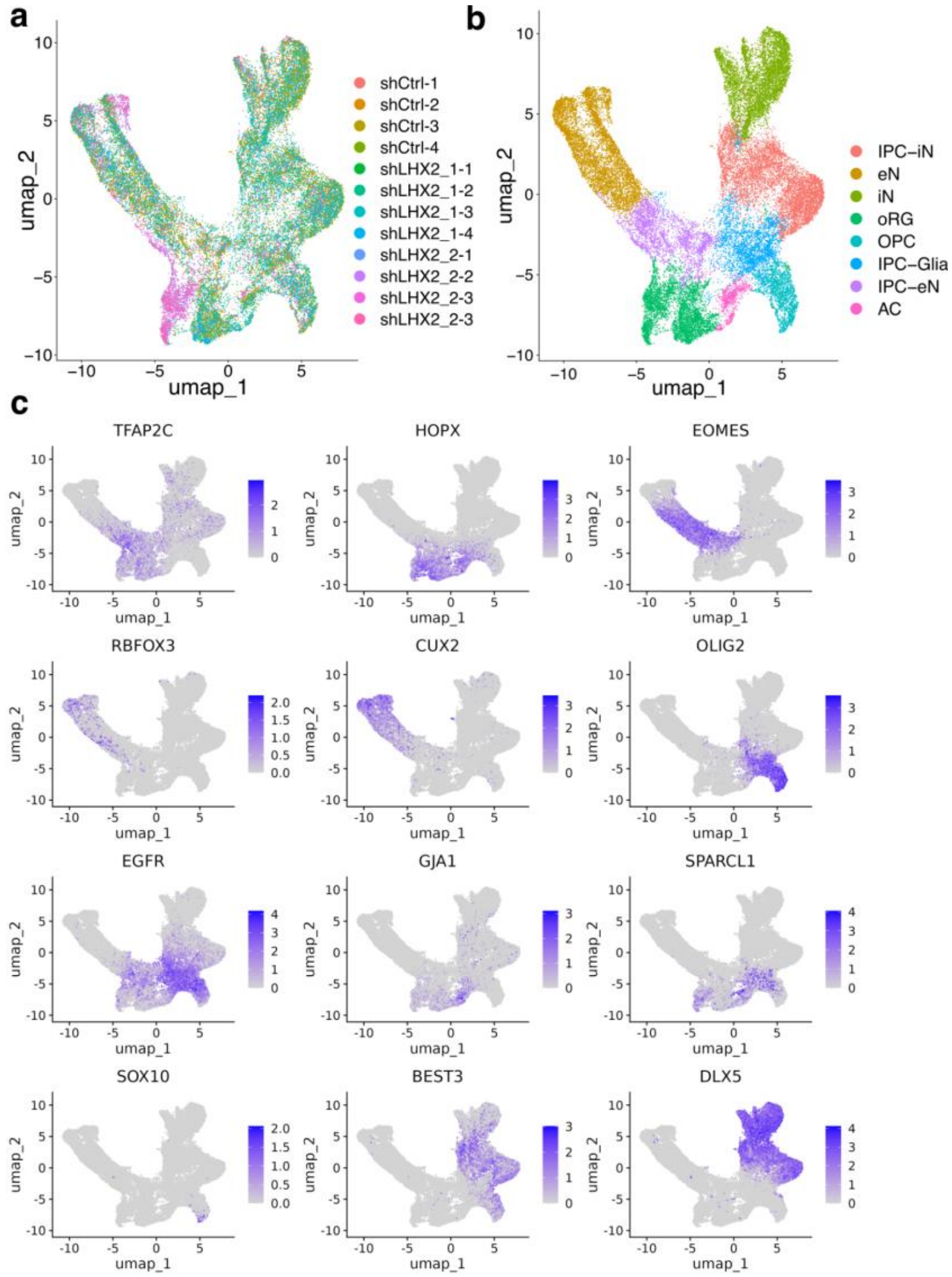

Ext. Fig. 8: a, b) UMAP plots of oRGs after 7 days of in vitro differentiation based on scRNA-seq, colored by (a) replicate, and (b) cell types. c) Scatterplot displaying the feature importance for each gene determined by scDist. The top 3 genes are highlighted.

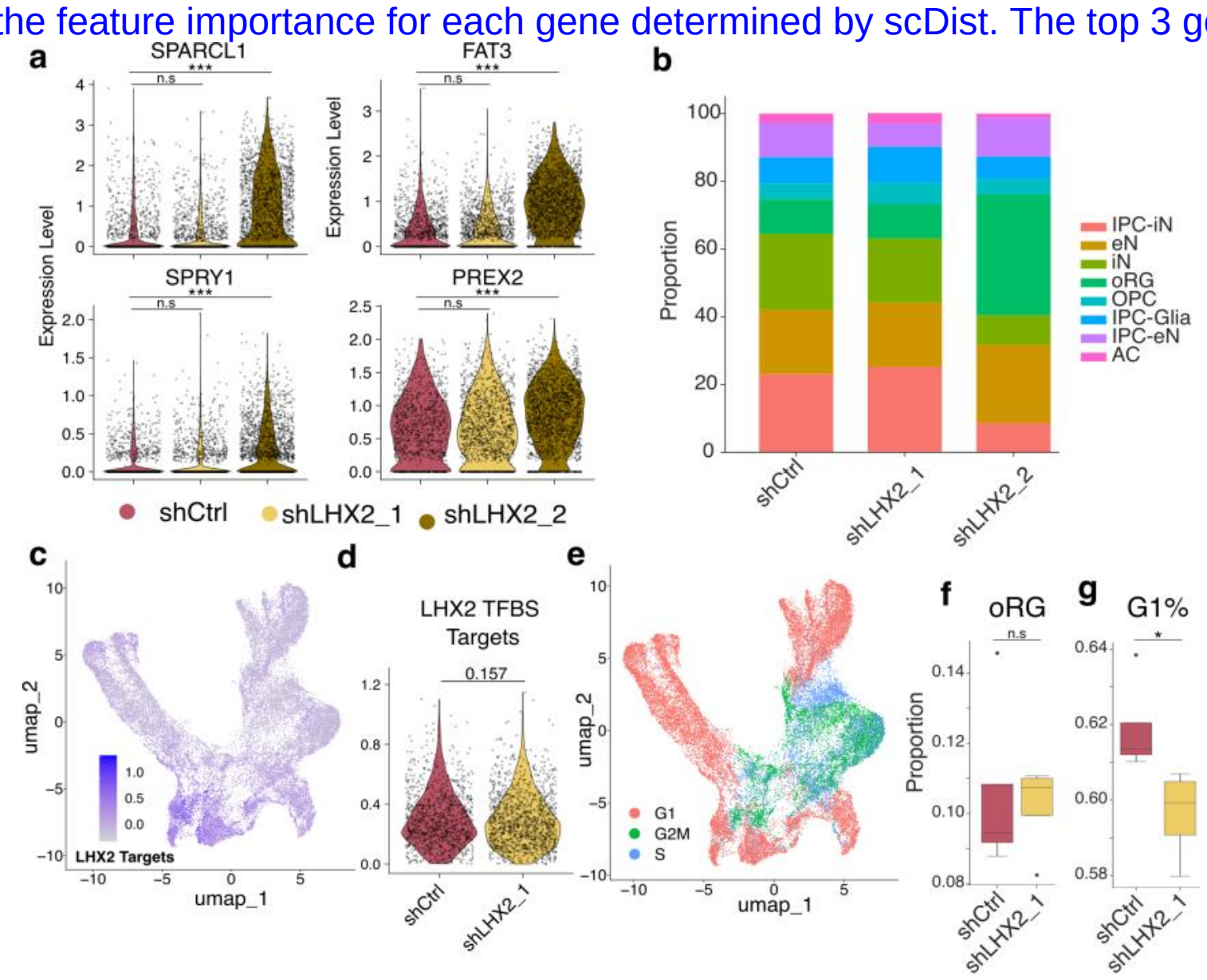

Ext. Fig. 9: a) Violin plots of the expression within the oRG cluster of genes perturbed by LHX2 knock down (Wilcoxon Rank Sum test, n.s: not significant, *: P< 0.05, **: P < 0.01, ***: P < 0.001). b) Barplots of the average proportion of cell types across four replicates. c) UMAP plots of oRGs after 7 days of in vitro differentiation based on scRNA-seq colors by module score of the 16 DEGs targets of the LHX2 motif within the oRG cluster d) Violin plot of the module score of the 16 DEGs targets of the LHX2 motif within the oRG cluster

(Wilcoxon Rank Sum test). e) UMAP plots of oRGs after 7 days of in vitro differentiation based on scRNA-seq colors by cell cycle phase. f) Boxplot of the proportion of oRG (scCODA, n.s: not significant). g) Boxplot of the proportion of cells at the G1 state within all clusters (Paired T-test, *: P< 0.05).

Referee #4 (Remarks to the Author):

The manuscript "3D Epigenome of Glial Cell Types in Developing Human Cortex" by Jones et al. provides a comprehensive examination of transcriptional regulation during neurogenesis, using multi-omics approaches to uncover key differences in radial glial cells (vRG, oRG), OPCs, and microglia during early human development. The authors employ bulk RNA sequencing with ATAC-seq and PLAC-seq to identify cell-type-specific cis-regulatory elements and validate these findings in mouse models. This innovative multi-omics strategy enables the identification of active genomic regions that control gene expression in a highly specific manner, according to cell type. The study highlights the critical role of transcription factor binding sites in regulating gene expression and driving cell fate specialization in oRGs and vRGs. Importantly, the research reveals that oRGs exhibit an exceptionally high density of cis-regulatory elements within HAR regions, offering novel insights into the regulatory mechanisms governing human cortex development. The authors apply machine learning algorithms to predict the effects of GWAS risk variants on gene regulation, focusing specifically on the rs4449074 variant associated with schizophrenia. This work has significant implications for understanding the intricate relationships between genetic variation and gene regulation in the developing brain. While this manuscript provides valuable insights into glial cell development, it also provides a detailed analysis of radial glial cells transitioning to oRGs and vRGs.

Regrettably the manuscript is an incremental step from previous work by the same lab (Song et al., 2020), where it extends the analysis to subtypes of radial glial cells but somewhat superficially includes OPCs and microglia. The manuscript would benefit from more comprehensive comparisons with the previous study, particularly those examining intergenic risk factors associated with GWAS variants across all cell types in human tissue at this stage. To further validate their findings, the authors could investigate what changes occur in oRG biology as a result of the rs4449074 variant compared to non-carriers. Moreover, functional validation of the predicted regulatory elements is essential to establish their biological significance and the causal role they play in controlling gene expression during neurogenesis. While the bioinformatic analysis provides a broad spectrum of insights, the absence of experimental validation reduces the impact of the study in its current form. Nevertheless, this research has the potential to address key questions and make significant contributions to our understanding of gene regulation during neurogenesis, ultimately informing human health and disease.

We sincerely thank Referee #4 for their thoughtful evaluation of our manuscript and for recognizing the novelty and potential impact of our multi-omic analysis of human glial development. The reviewer highlighted several important areas for improvement, including functional validation of predicted regulatory elements, direct comparisons with prior work (Song et al., 2020), and integration of psychiatric GWAS variants with cell type-specific regulatory programs. In response, we have conducted additional experiments and analyses and clarified key aspects of our data and interpretations to strengthen the manuscript.

First, we substantially expanded functional validation of candidate regulatory elements. Cross-validation with a published MPRA dataset in mid-gestation human cortical cells and cerebral organoids confirmed enhancer activity for a large fraction of predicted cCREs across vRG, oRG, OPC, and microglia. We also performed targeted shRNA-mediated knockdown of the transcription factor LHX2 in oRGs, coupled with single-cell RNA-seq, revealing transcriptional and cellular changes consistent with LHX2's predicted regulatory role, including reduced self-renewal and a shift toward an astrocyte-like state. Finally, CRISPR interference of the HARsv2_1313 in iPSC-derived neuronal precursors demonstrated its regulation of ROCK2 expression and impact on cell proliferation, establishing a causal link between this predicted regulatory element and a relevant cellular phenotype.

Second, we clarified the novel contributions of our study relative to Song et al., 2020 and extended disease-relevance analyses. Heritability enrichment analyses of accessible chromatin peaks stratified by 3D interactions reveal that interacting peaks are enriched for neuropsychiatric disease risk in both glial and neuronal cell types, whereas non-interacting peaks show enrichment primarily in glial. These results highlight how chromatin interactions refine disease-relevant regulatory elements and extend prior observations.

Third, we provide full predictions of variants across vRGs, oRGs, OPCs, and MG (Table 8) and further analyzed the impact of the rs4449074 genotype on SATB2 expression in vRGs using a recently published single-nucleus multiome dataset from matched developmental stages. We observed no statistically significant difference in SATB2 expression between individuals harboring the risk allele and those with the reference allele. This observation may be explained by three factors: (1) the limited sample size suggests that rs4449074 alone is not sufficient to measurably alter SATB2 expression at this developmental stage; (2) SATB2 may not be the target gene for the rs4449074-containing cCRE; or (3) the variant may have a modest enhancer effect or be subject to compensatory regulation by other CREs within the locus.

Together, these revisions provide robust functional validation and contextualize our findings relative to prior work, substantially strengthening the manuscript. We thank Referee #4 for these insightful suggestions, which have significantly improved the rigor, interpretation, and impact of our study.

Major points

1. The manuscript lacks experimental validation of the predicted regulatory elements, which is essential to establish their biological significance and the causal role they play in controlling gene expression during neurogenesis.

We agree with Referee #4 that functional validation is essential. To address the reviewer's concerns, we performed new experiments and analysis as the following: First, we performed cross-validation of our annotated cCREs with results from an MPRA study in mid-gestation human cortical cells and cerebral organoids (PMID 38781390). A total of 9,032, 9,027, 8,587, and 7,910 cCREs from vRG, oRG, OPC, and MG, respectively, were tested, representing 12.4-13.1% of cCREs. Across all four cell types in our study, the fraction of cCREs exhibiting enhancer activity in the MPRA, 44.5%-51.1%, was comparable to that reported in the original study, which selected elements either overlapping H3K27ac signal or chromatin interactions (Ext. Fig. 3f). These results support the functional potential of a subset of our predicted regulatory elements.

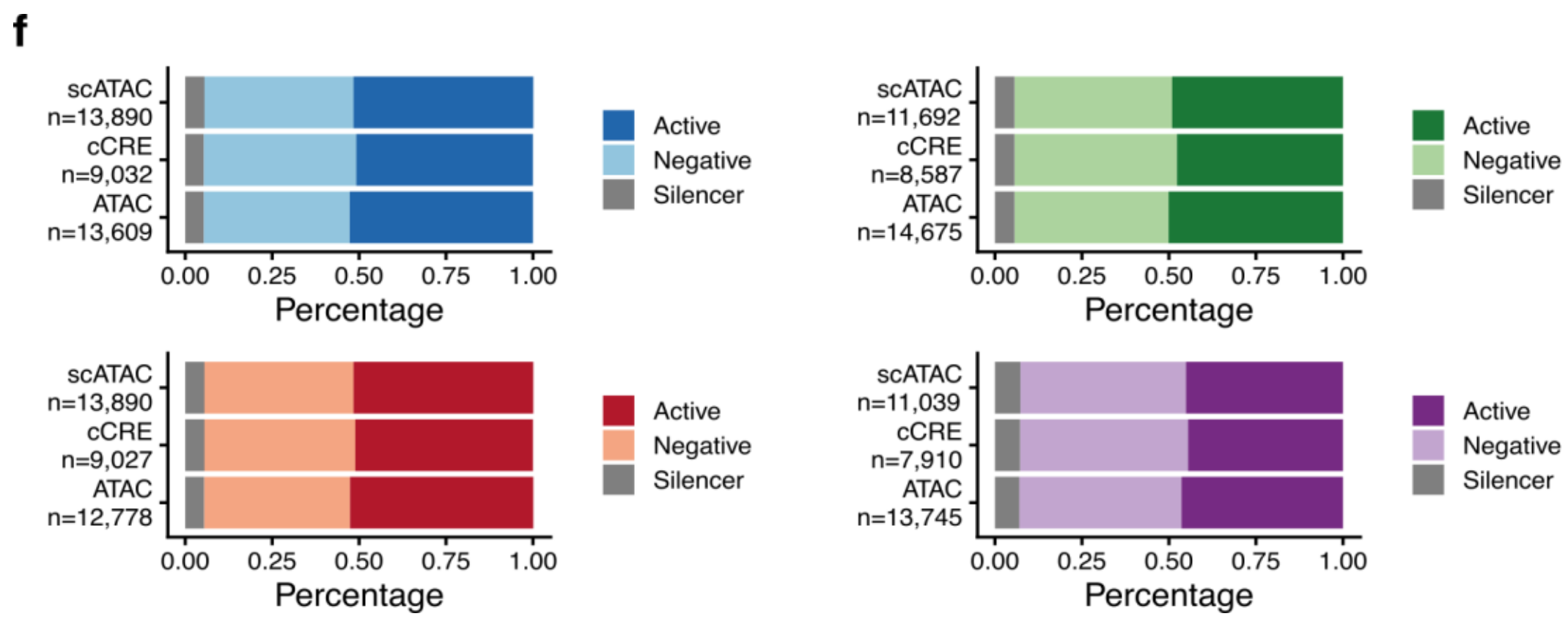

Ext. Fig. 3: f) Barplot of MPRA in mid-gestation human cortical cells that overlap cell type-specific peaks identified by scATAC (PMID 38781390), cCREs, and ATAC-seq peaks. For scATAC-seq, microglia, radial glia, and astrocyte/oligodendrocyte accessible peaks were used.

Second, we previously leveraged the VISTA Enhancer Database to assess in vivo enhancer activity and found that our cCREs are significantly enriched for elements with validated developmental enhancer function, further supporting their biological relevance.

Third, we conducted targeted functional validation of the transcription factor LHX2 to directly assess its cell type-specific role in oRGs, guided by our regulatory network predictions.

Finally, we performed CRISPR-based perturbation experiments demonstrating that the human accelerated region HARsv2_1313 regulates ROCK2 expression and influences cell proliferation, establishing a causal link between a predicted regulatory element, its target gene, and a relevant cellular phenotype.

These new experiments and analyses provide strong evidence supporting the functional significance of our predicted regulatory elements, substantially strengthen the manuscript, and directly address the reviewer's concerns regarding biological validation.

The manuscript relies heavily on bioinformatic analysis to identify potential regulatory elements, but lacks experimental validation of the predicted regulatory elements. This is essential to establish their biological significance and the causal role they play in controlling gene expression during neurogenesis, and such as driving cell fate specialization in oRGs and vRGs,. To address this, the authors could for example conduct functional assays (e.g. CRISPR-Cas9 knockout or overexpression experiments) in organoids, or fetal cell cultures to demonstrate that these predicted regulatory elements indeed control gene expression during neurogenesis.

For examples are CRE switches needed for the transition or the consequence of the transition? LIFR signalling has been shown to regulate oRG numbers in organoids and is indicated here in the network of interactions for LHX2. The role of LHX2 in human RG function is not clear, does its regulation and expression have a developmental effect. Would any of the identified CRE candidates and TFs such as LHX2 alter vRG to oRG transition? There is no evidence for the TF binding to the cCREs in RGs or data showing alteration or removal of the changes caused by putative binding site alterations.

Functional studies would provide necessary evidence for the biological relevance of these findings and strengthen the conclusions drawn from the bioinformatic analysis.

We appreciate the reviewer's emphasis on functional validation and have addressed this by performing targeted perturbation experiments focused on the key candidate regulator LHX2, an oRG-associated TF, to assess its cell-type-specific role, as described at line 228.

We performed short hairpin RNA (shRNA)-mediated knockdown followed by single-cell RNA sequencing (scRNA-seq) to examine its impact on transcription and neuronal differentiation. UMAP clustering identified eight distinct cell types, with minimal batch effects (Ext. Fig. 8a-c). Successful knockdown of LHX2 was confirmed within the oRG cluster, with shLHX2_2 producing a more pronounced reduction than shLHX2_1 (Fig. 3c). To identify cell types that exhibited transcriptomic dysregulation upon LHX2 knock down, we applied scDist (PMID 39218971), a statistically rigorous method to fairly rank transcriptomic changes based on the Euclidean distance. Both shRNAs produced concordant directional transcriptomic changes, but the effect was substantially greater with shLHX2_2, consistent with its stronger knockdown efficiency (Fig. 3d). Subsequent analyses therefore focused on shLHX2_2. The oRG and astrocytes (AC) clusters were the most perturbed, with the AC marker SPARCL1 being of high feature importance (Ext. Fig. 9a), in agreement with prior work suggesting that LHX2 is required to repress astrogenesis (PMID 21690374). In addition to the increase of SPARCL1, we observed a general upregulation of the 16 DEGs predicted as targets of LHX2 TFBS, including FAT3, SPRY1, PRKCA, and PREX2 (Fig 3e, Ext. Fig. 9a, c). These results support a model in which LHX2 regulates AC-associated genes and downstream differentiation trajectories. Furthermore, the relative abundance of oRG cells increased significantly following shLHX2_2 knockdown at the expense of differentiated neurons (Fig. 3f. Ext. Fig. 9b). Despite this relative expansion, shLHX2_2 exhibited a higher proportion of cells in G1 phase (Fig. 3g; Ext. Fig. 9e), indicating reduced proliferative activity and the onset of a transition toward differentiation or quiescence. Taken together, loss of LHX2 reduces self-renewal and shifts oRG towards a more differentiated, AC-like state. Consistent with a weaker perturbation, shLHX2_1 showed a similar transcriptomic trend but did not produce comparable compositional or cell-cycle changes, likely due to incomplete depletion of LHX2 (Ext. Fig. 9d, f-g).

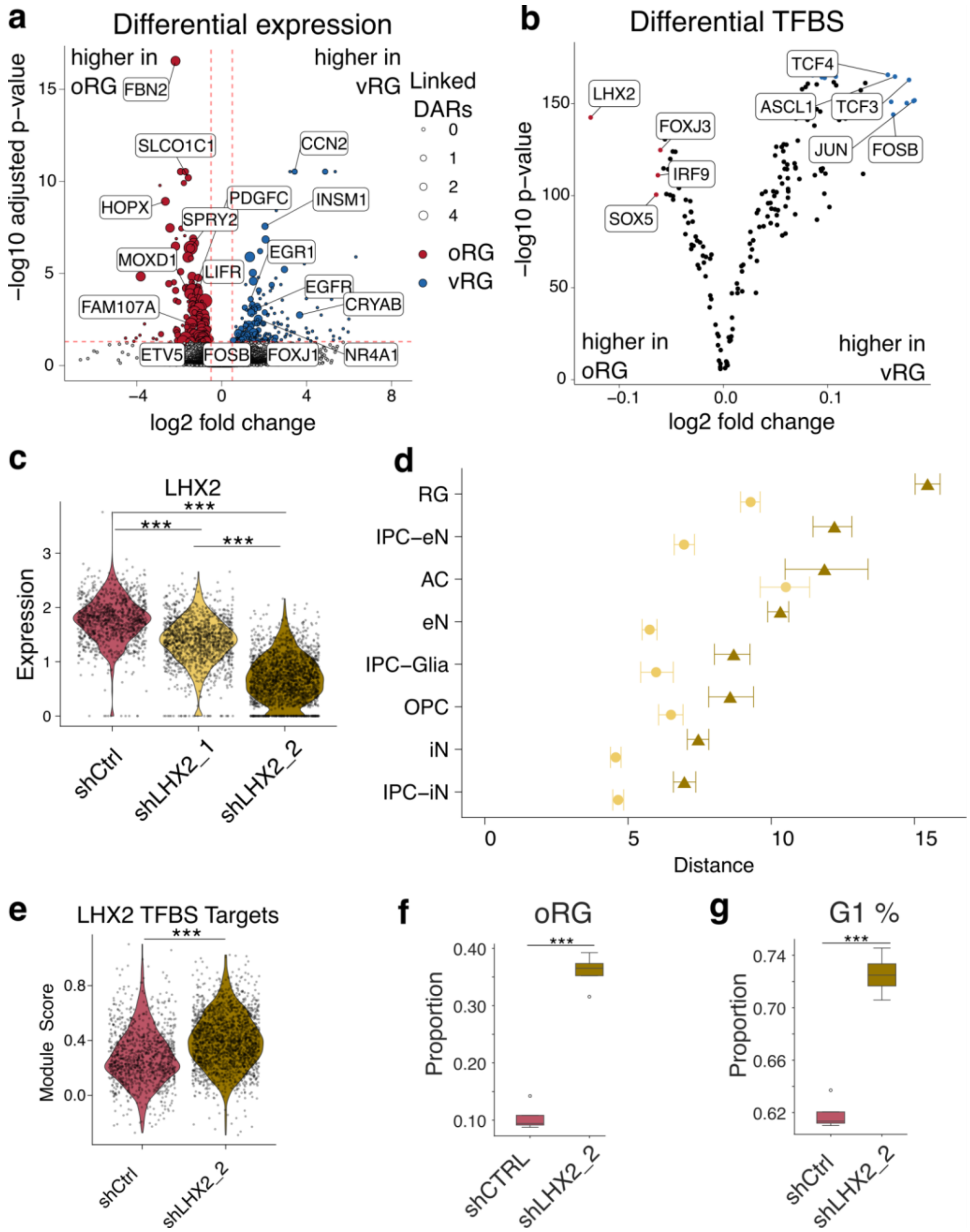

Fig. 3: a) Volcano plot of differentially expressed genes. The size of each dot is determined by the amount of DARs overlapping the TSS or interacting with each gene. b) Volcano plot of motif binding difference between vRG and oRG, using TOBIAS Footprinting Analysis. c) Violin plot of *LHX2* expression within the oRG cluster. (Wilcoxon Rank Sum test, *: P< 0.05, **: P < 0.01, ***: P < 0.001). d) Distance score obtained by scDist for each cell type. e) Violin plot of the module score of the 16 DEGs targets of the LHX2 motif within the oRG cluster. (Wilcoxon Rank Sum test, *: P< 0.05, **: P < 0.01, ***: P < 0.001). f) Boxplot of the proportion of oRG. (scCODA, *: P< 0.05, **: P < 0.01, ***: P < 0.001). g) Boxplot of the proportion of cells at the G1 state within the oRG cluster (Paired T-test, *: P< 0.05, **: P < 0.01, ***: P < 0.001).

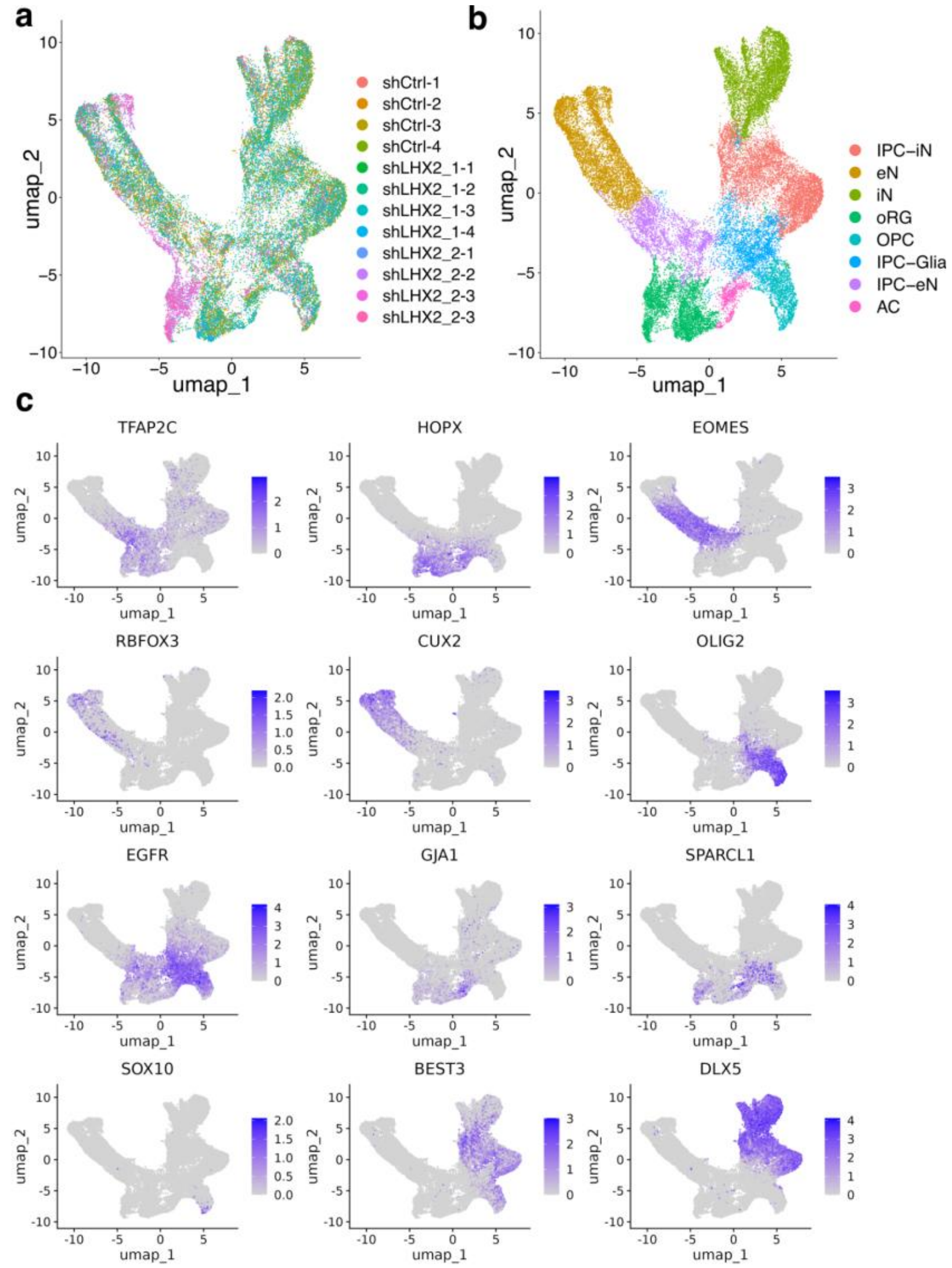

Ext. Fig. 8: a, b) UMAP plots of oRGs after 7 days of in vitro differentiation based on scRNA-seq colors by (a) replicate, and (b) cell types. c) Scatterplot displaying the feature importance for each gene determined by scDist. Top 3 genes are highlighted.

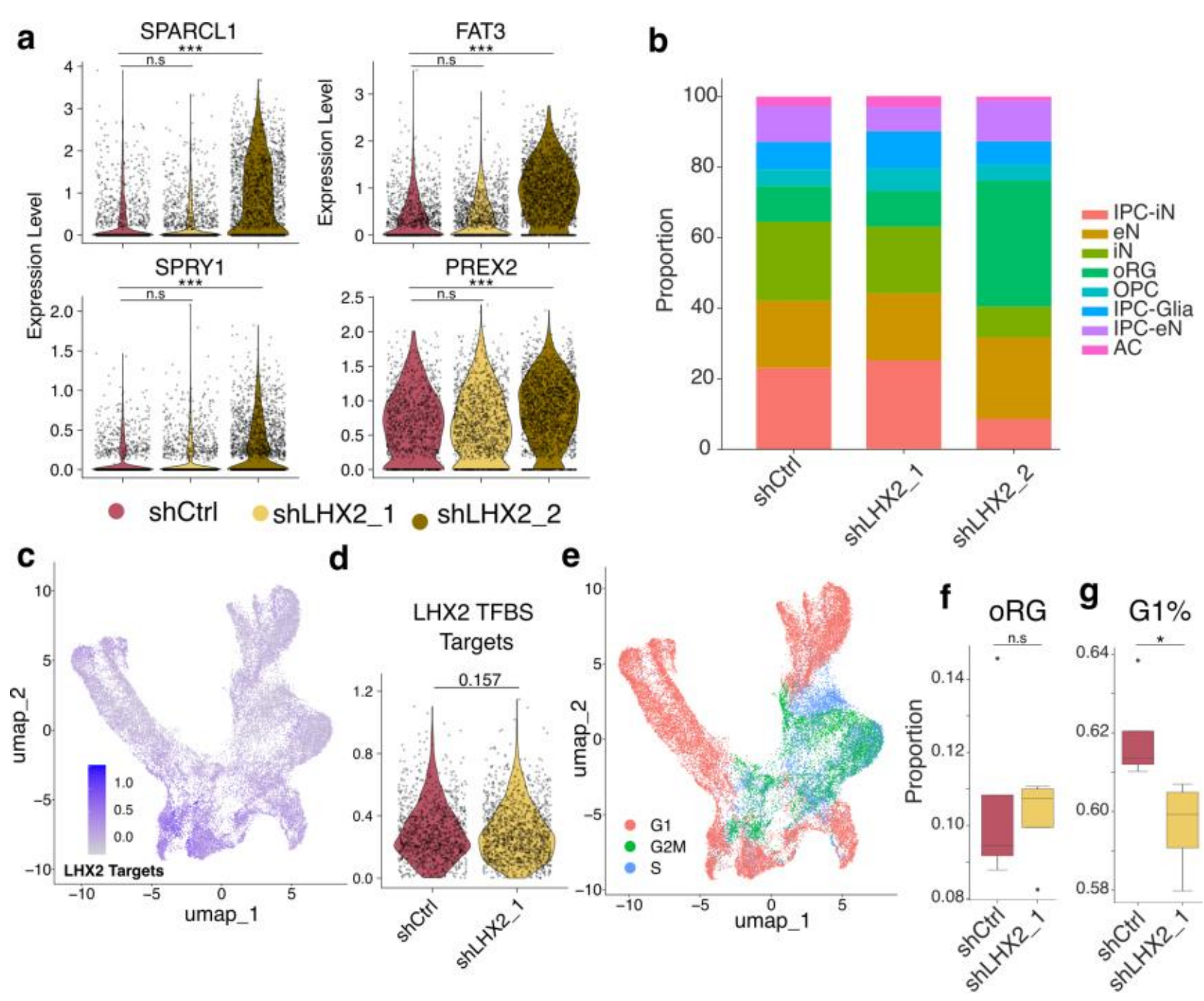

Ext. Fig. 9: a) Violin plots of the expression within the oRG cluster of genes perturbed by LHX2 knock down (Wilcoxon Rank Sum test, n.s: not significant, *: P< 0.05, **: P < 0.01, ***: P < 0.001). b) Barplots of the average proportion of cell types across four replicates. c) UMAP plots of oRGs after 7 days of in vitro differentiation based on scRNA-seq colors by module score of the 16 DEGs targets of the LHX2 motif within the oRG cluster d) Violin plot of the module score of the 16 DEGs targets of the LHX2 motif within the oRG cluster (Wilcoxon Rank Sum test). e) UMAP plots of oRGs after 7 days of in vitro differentiation based on scRNA-seq colors by cell cycle phase. f) Boxplot of the proportion of oRG (scCODA, n.s: not significant). g) Boxplot of the proportion of cells at the G1 state within all clusters (Paired T-test, *: P< 0.05).

Moreover, we further investigated the function of HARsv2_1313, for which the human-specific G allele at chr2:11,301,810 is predicted to reduce chromatin accessibility relative to the ancestral A allele in oRGs (Fig 5c). HARsv2_1313 interacts with the promoter of ROCK2 (Fig. 5d). CRISPR interference (CRISPRi) using paired guide RNAs (gRNAs; Table 10) in human iPSC-derived neuronal precursor cells (NPCs) demonstrated decreased ROCK2 expression, confirming HARsv2_1313 functions as an enhancer of ROCK2 (Fig. 5e). Consistent with prior evidence that ROCK inhibition promotes stem cell proliferation and viability (PMID 19235204), we observed a significant increase in Ki67-positive cells in EMX+ NPC population following knockdown of the ROCK2 promoter and HARsv2_1313 with one pair of gRNAs, whereas the second pair showed a similar trend toward increased proliferation (Ext. Fig. 14d). Moreover, the percentage of Ki67 was positively correlated with the gRNA-associated green fluorescent protein (GFP) signal in all conditions relative to control (slope difference = 3.48-5.86, P < 0.001) (Fig. 5f). Together, these results suggest that reduced HARsv2_1313 activity in humans decreases ROCK2 expression and increases proliferation, in agreement with prior CRISPR activation experiments in iPSC-derived neurons (PMID 36640767) and with higher ROCK2 expression in chimpanzee organoids compared to human organoids (PMID 30735633).

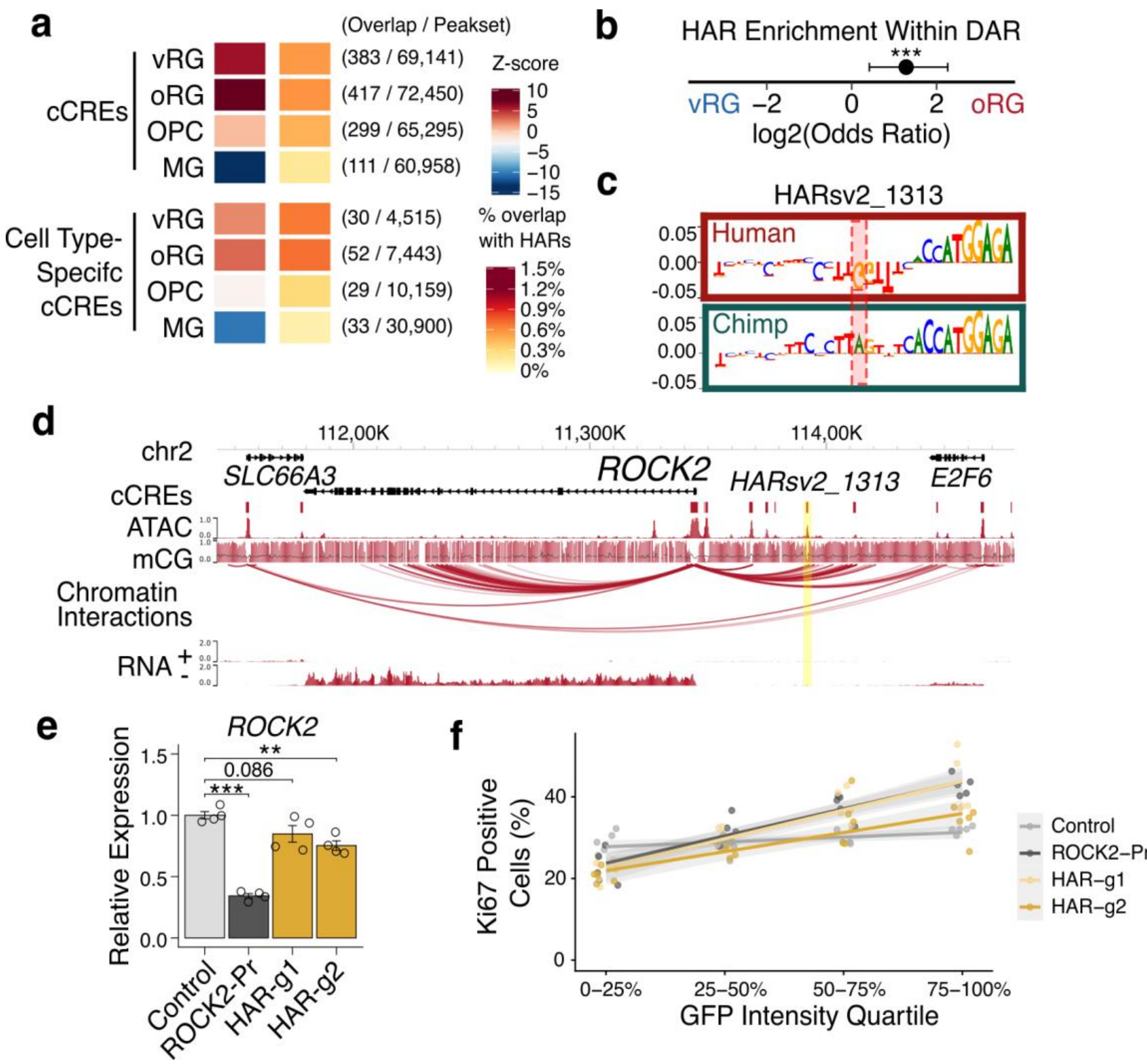

Figure 5: a) Heatmap displaying the z-score (left) for overlap of either cCREs or cell type-specific cCREs compared with a randomly sampled empirical null distribution as well as the percentage of HARs overlapping the data (right). b) Forest plot showing the association of vRG or oRG DARs with HARs. Data are shown as odds ratio with 95% confidence interval (Fisher's exact test, *: P < 0.05, **: P < 0.01, ***: P < 0.001). c) Overview of HARsv2_1313. Top GkmExplain importance scores for each base pair surrounding the human G allele. Bottom, GkmExplain importance scores for each base pair surrounding the chimpanzee ancestral A allele. d) WashU browser of the

ROCK2 locus. HARv2_1313 highlighted in yellow. e) Expression of ROCK2 in NPC based on RT-qPCR. Four independent differentiations per condition were used. (two-sided t-test, *: P < 0.05, **: P < 0.01, ***: P < 0.001). f) Percentage of cells positive for Ki67+ in NPC based on immunocytochemistry stratified into quartiles by GFP intensity. Linear regression lines are shown with 95% confidence intervals.

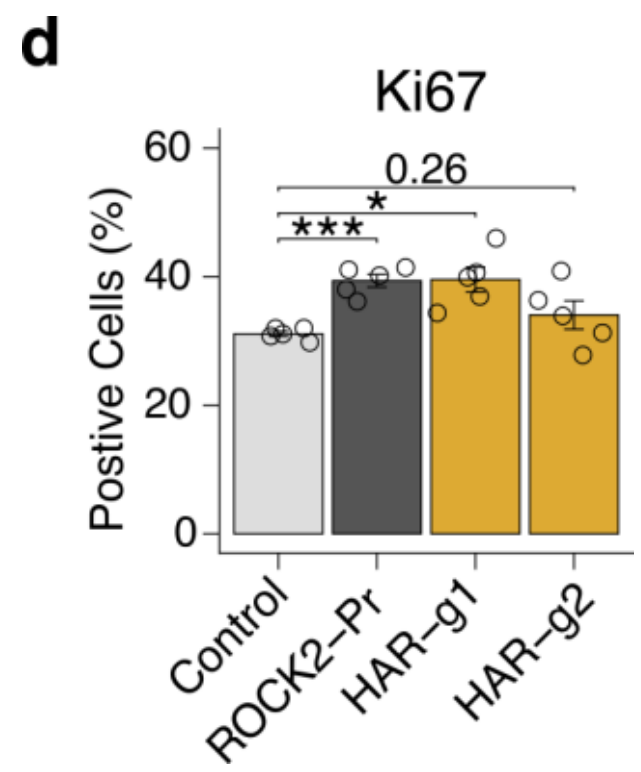

Figure 14: d) Percentage of cells positive for Ki67+ in NPC based on immunocytochemistry. Five independent differentiations per condition were used (two-sided t-test, *: P < 0.05, **: P < 0.01, ***: P < 0.001).

Together, these functional experiments provide validation of both a key oRG-associated TF and a human-specific enhancer, supporting the causal role of predicted regulatory elements in controlling transcriptional programs and cell type-specific differentiation during human neurogenesis.

2. The manuscript builds upon previous work from the same lab (Song et al., 2020) but could benefit from more detailed comparisons to highlight its novel contributions and extend our understanding of early human neurodevelopment. In particularly for examining intergenic risk factors associated with GWAS variants and all cell types in human tissue at this stage. This would provide a more understanding of how these regulatory elements interact with genetic variation and gene regulation during neurogenesis.

We appreciate this suggestion by Referee #4 and address a similar concern to Referee #2.

To enable direct comparison with the datasets used in Song et al., we repeated the heritability enrichment analysis using accessible chromatin peaks. We stratified peaks into those participating in significant 3D chromatin interactions and those that do not. We found that accessible peaks involved in 3D interactions are enriched for neuropsychiatric disease heritability in both glial and neuronal cell types. In contrast, non-interacting accessible chromatin shows enrichment for neuropsychiatric traits exclusively in glial cell types.

These results indicate that chromatin interactions refine disease-relevant regulatory elements, particularly in neuronal lineages, whereas a subset of non-interacting accessible regions in glial cells may still contribute to disease risk through interaction-independent regulatory mechanisms. We have incorporated these results into the revised manuscript.

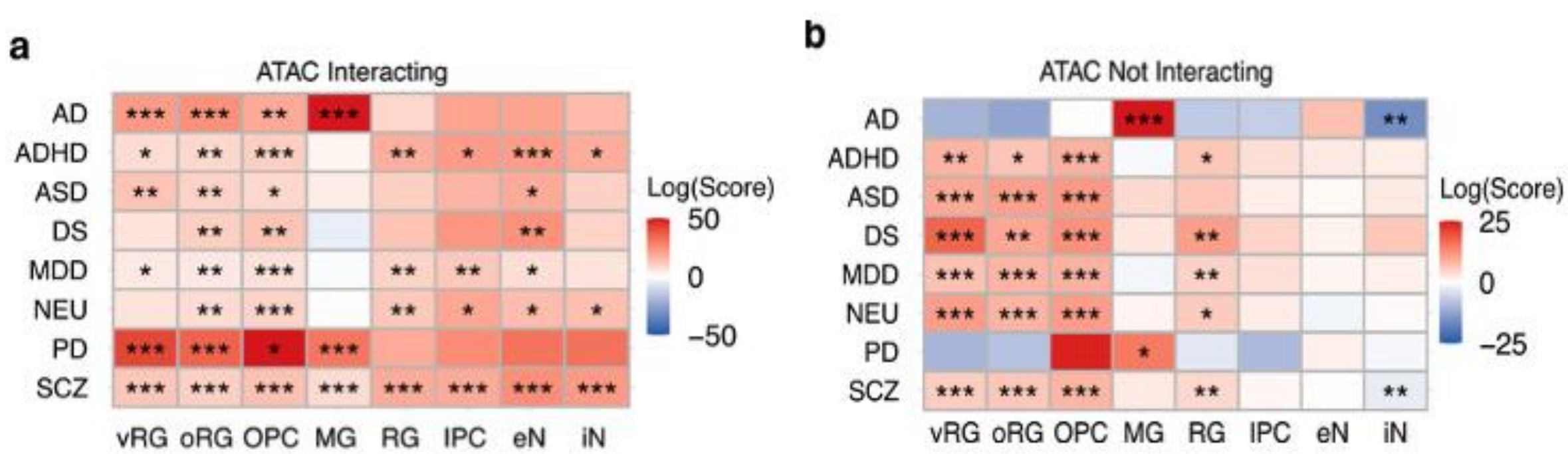

Ext. Fig. 10: Heatmap of LDSC score for (a) ATAC-seq peaks involved in 3D interaction (b) ATAC-seq peaks not involved in 3D interactions. Red, the disorder is positively enriched. Blue, the disorder is negatively enriched. (*: P < 0.05, **: P < 0.01, ***: P < 0.001).

3. The manuscript highlights the importance of transcription factor binding sites in CREs in regulating gene expression and driving cell fate specialization in oRGs and vRGs, the authors could delve deeper into how psychiatric GWAS hits interact with these regulatory elements, and their implications in various neuropsychiatric disorders. Importantly the authors should investigate whether these psychiatric GWAS hits affect the predicted regulatory elements, particularly for distal sequences, which would provide valuable insights into the relationship between genetic variation and gene regulation in the developing brain.

In Figure 4, Ext fig 8 the authors correlate schizophrenia and GWAS variant rs4449074 with RGs however their data indicates higher MG and OPC SNP accessibility changes, but these changes not investigated. It is unclear if the SATB2 variant is sufficient to cause changes in upper layer specification or neuron numbers during development? This could be also tested using in vitro organoid or ex vivo slice models.

We thank Referee #4 for this suggestion. We agree that predicted TF binding should be considered when prioritizing GWAS variants. Inspired by prior work (PMID 33106633), we provide a 'prominence score' in supplemental tables and P values, which identifies seqlets that could match TF motifs. Building on this work, we also provide a prominence score for negative or repressive contributions from seqlets. For example, in Supplemental Table 9_6 HARsv2_2157_2, which is predicted to bind repressor ZEB1, does not have a significant (P=0.199) prominence score for a positive seqlet relative to shuffle controls, but is significant (P=0.013) for a negative prominence score. Unlike motifBreakR, which relies solely on sequence, prominence scores provide cell type-specific predictions.

We chose to validate and focus on rs4449074 within vRG for three main reasons: (1) prior VISTA enhancer testing identified this locus as active in the cortex; (2) vRG cCREs are enriched for SCZ heritability based on LDSC analysis; and (3) vRG cCREs were shown to be well-suited for transgenic mouse experiments.

To directly address whether rs4449074 influences the expression of the nearest gene SATB2, we analyzed our recently published single-nucleus multiome dataset from matched developmental stages (PMID 39779846), extending our prior bulk accessibility analyses shown in Ext. Fig. 8g. Specifically, we examined SATB2 expression stratified by rs4449074 genotype. Among the nine individuals analyzed, three carried the risk allele. Within vRGs, we observed no statistically significant difference in SATB2 expression between individuals harboring the risk allele and those with the reference allele. There are three reasons that can explain this observation: First, although this analysis is limited by sample size, these results suggest that rs4449074 alone caused changes in accessibility and is insufficient to measurably alter SATB2 expression at this developmental time point in vRGs. Second, STAB2 may not be the target gene for the rs4449074 containing cCRE. Third, this may reflect a modest enhancer effect or compensatory activity from other cis-regulatory elements within the locus.

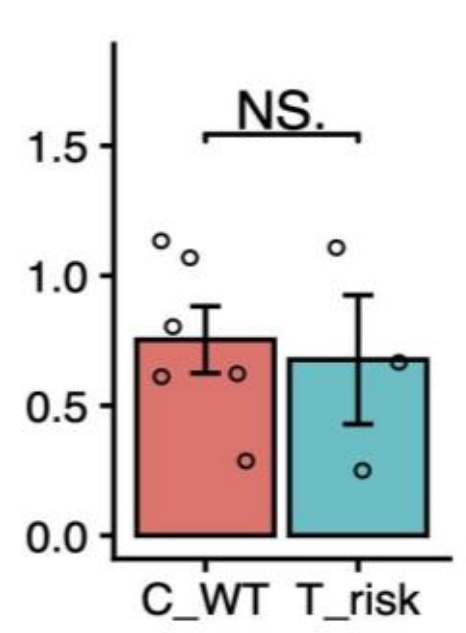

Boxplot of expression within vRG for individuals with reference allele (C_WT) or risk allele (T_risk).

Finally, rs4449074 is most strongly predicted to perturb chromatin accessibility in vRGs (explain score = −0.63, P = 0.017). In contrast, predicted effects in oRGs, OPCs, and microglia are substantially weaker (explain scores −0.36, −0.31, and −0.08, respectively) and not statistically significant (all P > 0.1). We agree with Referee #4 that psychiatric GWAS variants impacting MG and OPC regulatory programs are of considerable interest. Accordingly, we provide our full accessibility and explain-score predictions for these cell types in Table 8 to facilitate further exploration.

Referee #1 (Remarks to the Author):

The authors have done a comprehensive work in addressing the comments. I have one additional comment related to one of the responses.

With additional analyses, the authors showed that 75% PLAC-seq interactions do not involve significant signatures of regulatory elements, defined by VISTA-validated enhancers, LMRs, or accessibilities. Therefore, these regions are considered less interesting in the context of this study. This is a reasonable, although unexciting, conclusion. Are these regions more related to the structural elements of chromatin folding, such as those bound by CTCF or cohesin? The authors should discuss these results in the manuscript, including the negative finding that 75% of interactions do not engage regulatory activity, for completeness.

We appreciate Referee #1's suggestion to include our findings regarding the 75% of PLAC-seq interactions that do not show significant regulatory signatures. We agree this provides important context and have now added a corresponding figure (Ext. Fig. 3l) and the following description to the text: "Distal interacting bins lacking accessibility or low methylation, which account for ~75% of XOR interactions, showed no or weak enrichment for VISTA enhancers, indicating minimal enhancer-like activity in these regions."

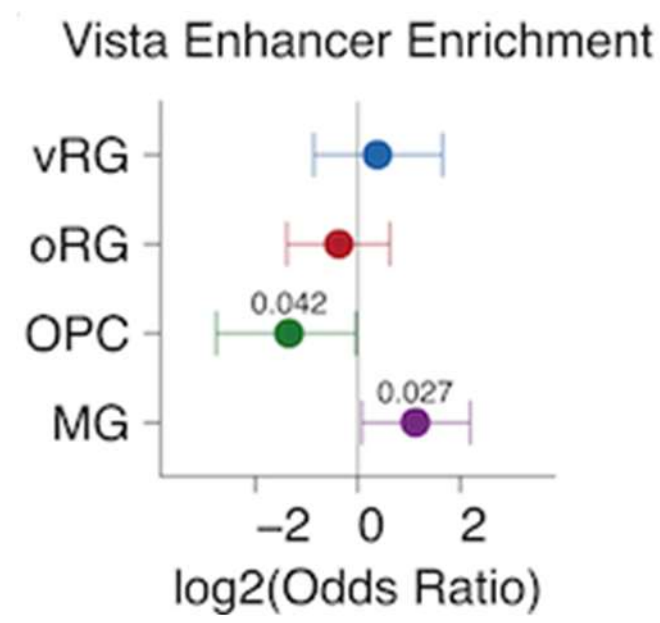

Extended Figure 3: l) Forest plot showing the association of distal interacting bins lacking accessibility or low methylation with positive neural VISTA elements or negative neuronal VISTA elements. Each point represents the log2 odds ratio, with horizontal lines indicating 95% confidence intervals. 44-156 total genomic regions tested.

The referee raises an interesting question about whether these regions might be related to structural chromatin elements such as CTCF or cohesin binding sites. To address this, we tested for enrichment of the CTCF motif using HOMER compared to background. The motif was present but not significantly enriched in these regions, 4.45%, 4.56%, 4.22%, and 4.21% in vRG, oRG, OPC, and MG 2kb interactions lacking accessibility or low methylation, respectively, compared with 5.12%, 5.20%, 5.32%, and 5.38% of background elements. Notably, CTCF is significantly enriched in cell-type specific cCREs (Fig 1f), suggesting that CTCF binding is not driving the interactions that lack regulatory signatures. One possibility is that a portion of these interactions may cluster near other cCREs and contribute to chromatin architecture through proximity effects rather than direct regulatory activity.

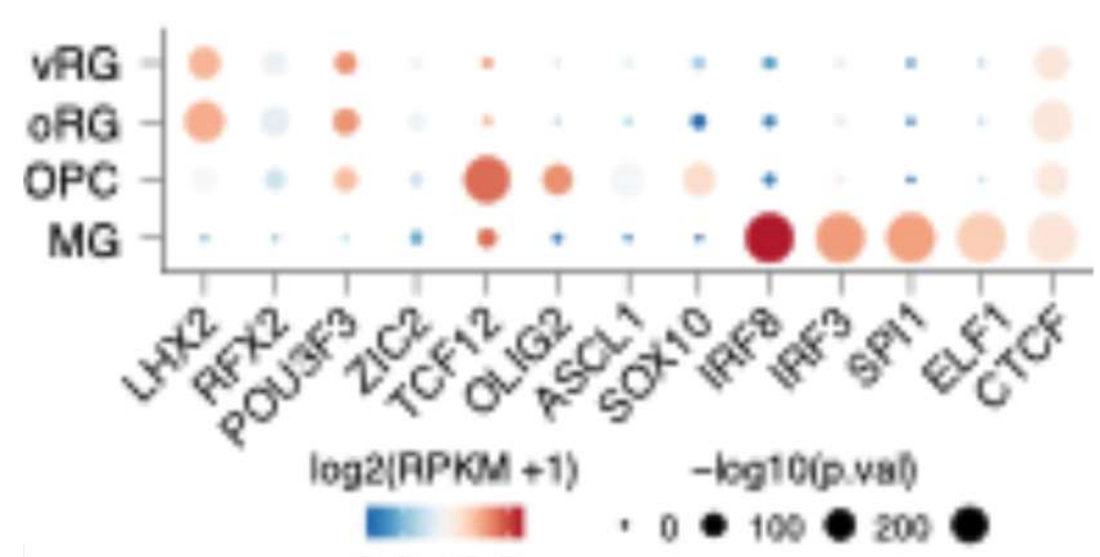

Figure 1: f) TF enrichment analysis for cell type-specific cCREs. 4,515, 7,443, 10,159, 30,900 peaks were used from vRG, oRG, OPC and MG respectively. Colors represent RPKM expression of the corresponding TFs, and dot sizes represent enrichment p-values from HOMER (one-sided binomial test).

Referee #2 (Remarks to the Author):

The authors addressed all of my comments properly and likewise, those raised by the other Reviewers. The paper is now much improved.

We really appreciate Referee #2's constructive comments, which have contributed significantly to the improvement of our manuscript.

Referee #3 (Remarks to the Author):

In their revised submission "3D Epigenome of Glial Cell Types in Developing Human Cortex," Jones, Wang et al provide a detailed response to feedback from all reviewers; in my opinion, they very well addressed all concerns! I am very satisfied with the improved clarity by which data is now presented by replicate in SI Fig. 1. Likewise, I commend the authors with their efforts towards functional validation, incorporating a published MPRA dataset to validate CREs and new shRNA- and CRISPR-mediated experiments to test a top transcription factor and HAR identified in their analyses. Finally, the authors did a thoughtful job integrating their analyses with their previous study of 3D genome structure in neurons as well as with cross-disorder GWAS. This manuscript is a pleasure to read and will be a valued resource to the field. I am happy to recommend it for publication at Nature!

- Kristen Brennand

We are truly grateful for Referee #3's constructive comments, which have contributed significantly to the improvement of our manuscript.

Referee #4 (Remarks to the Author):

The manuscript has improved substantially since the previous submission, and the authors have clearly made a serious effort to address the reviewers' concerns. The addition of functional data and further bioinformatic analyses has significantly strengthened the study. A small number of points remain where clarification would improve interpretability.

The main outstanding issue may reflect a misunderstanding on the reviewer's part; however, the current presentation makes it difficult to reconcile some conclusions with the data, particularly regarding the functional experiments on HARsv2_1313. The main outstanding issue may reflect a misunderstanding on the reviewer's part; however, the current presentation makes it difficult to reconcile some conclusions with the data, particularly in relation to the functional experiments on HARsv2_1313. Specifically,

- ROCK2 knockdown reduces ROCK2 mRNA and increases Ki67$^+$ cells;
- HARg1 perturbation shows little or no reduction in ROCK2 mRNA, yet also increases Ki67;
- HARg2 perturbation leads to a stronger reduction in ROCK2 mRNA, but does not increase Ki67.

This pattern does not support a consistent relationship between ROCK2 levels and proliferation. Therefore, statements such as "...these results suggest that reduced HARsv2_1313 activity in humans decreases ROCK2 expression and increases proliferation…" and "...CRISPR interference… established a causal link between this predicted regulatory element and a relevant cellular phenotype...", appear overstating the data.

It would therefore be helpful if the authors could clarify whether the proliferation effects are mediated via ROCK2, or whether HARsv2_1313 may have additional targets mediating these effects. Alternatively, a mechanistic explanation for the apparent decoupling between ROCK2 mRNA levels and proliferation in the HARg1 versus HARg2 perturbations would strengthen the interpretation.
Additional supporting data would also improve confidence in these conclusions, including evidence for ROCK2 protein levels or kinase activity, alternative measures of proliferation beyond Ki67, and representative images for the quantifications.

Minor points

1. Extended Figure 3f: please clearly indicate the cell type corresponding to each bar.
2. Minor typographical errors should be corrected throughout, such as "Isolation and in vivo culture of oRG" — "in vivo" should likely read "in vitro"; Figure 2b-"Count stength" should read "Count strength" etc.

We appreciate Referee #4's positive assessment of the revisions and their thoughtful comments regarding the functional experiments on HARsv2_1313. We appreciate the opportunity to clarify our interpretation of these data.

The reviewer raises an important point regarding the relationship between *ROCK2* expression and proliferation across the different perturbation conditions. We agree that, when considering the RT-qPCR measurements alone, the relationship is not immediately apparent. However, we believe that several technical and biological considerations help explain these observations and that, taken together, our data continue to support the proposed regulatory relationship while acknowledging that the underlying mechanism is likely more complex than can be fully resolved in the present study.

A key technical consideration is that the RT-qPCR measurements were performed on heterogeneous cell populations containing both GFP-positive (successfully transduced) and GFP-negative cells. Although puromycin selection substantially enriched for transduced cells, flow cytometry analysis indicated that approximately 80–90% of cells were GFP-positive. Consequently, the measured *ROCK2* mRNA levels represent an average across mixed populations and likely underestimate the magnitude of gene repression within successfully transduced cells. This consideration is particularly relevant for HARg1, where dilution of the transcriptional response may explain why a statistically significant reduction in *ROCK2* mRNA was not detected despite the observed effect on proliferation.

To address this limitation, we performed a within-well analysis of Ki67-positive cells, comparing cells according to GFP signal intensity as a proxy for perturbation strength rather than relying solely on well-level averages. This approach minimizes confounding arising from differences in transduction efficiency and provides a more sensitive assessment of perturbation effects. Using this analysis, all three perturbations (*ROCK2* promoter gRNA, HARg1, and HARg2) exhibited a significant positive correlation between GFP intensity and Ki67 positivity relative to control (slope difference = 3.48–5.86, $P < 0.001$). Thus, a significant dose-dependent relationship between perturbation strength and proliferation was observed within that condition.

Our chromatin interaction data further identify ROCK2 as the primary direct regulatory target of HARsv2_1313, with no detectable interactions involving neighboring genes. While these findings support ROCK2 as the most likely direct target of this regulatory element, we agree that the present data do not fully establish that the proliferation phenotype is mediated exclusively through ROCK2. Additional downstream signaling events or indirect effects may also contribute to the observed phenotype. We have therefore revised the manuscript to better reflect current evidence and to distinguish between the direct regulatory relationship and the downstream cellular consequences as follows: ***We note that technical considerations, including incomplete transduction efficiency and potential indirect effects on other pathways. may contribute to variability in the observed relationship between ROCK2 mRNA levels and proliferation. Nevertheless, our findings support HARsv2_1313 as a regulator of ROCK2 expression and suggest that perturbation of this regulatory element is associated with increased proliferation, although the precise mechanism linking these observations remains to be fully established. These observations are also consistent with previous findings that CRISPR activation of this HAR increased ROCK2 expression in iPSC-derived neurons[60] and that ROCK2 expression is higher in chimpanzee organoids compared to human organoids[7].***

We appreciate the reviewer's suggestions for additional supporting data. We agree that measurements of ROCK2 protein abundance and/or kinase activity, alternative proliferation assays, and representative microscopy images would further strengthen the mechanistic interpretation. However, these experiments are beyond the scope of the current study and would require substantial additional experimental work.

We have also addressed the minor comments by clarifying the cell identities in the original Extended Figure 3f (now Extended Figure 2f) and correcting the typographical errors identified by the reviewer.

We hope these clarifications and revisions provide additional context for interpreting our findings and adequately address the reviewer's concerns
